# Near‐Unity Selectivity Inversion Between CO_2_ Electroreduction and H_2_ Evolution via Atomic Coordination Editing

**DOI:** 10.1002/advs.75911

**Published:** 2026-06-01

**Authors:** Yukun Zhao, Yuanyuan He, Mengyu Duan, Peng Yin, Jinxing Chen, Xu Han, Jie Yang, Yulin Wang, Xingjie Fu, Shibo Xi, Chuncheng Chen, Jiong Lu

**Affiliations:** ^1^ Department of Chemistry National University of Singapore Singapore Singapore; ^2^ College of Material and Textile Engineering Jiaxing University Jiaxing Zhejiang China; ^3^ Key Laboratory of Photochemistry CAS Research/Education Center for Excellence in Molecular Sciences Institute of Chemistry Chinese Academy of Sciences Beijing China; ^4^ Institute of Sustainability for Chemicals Energy and Environment (ISCE2) Agency for Science, Technology and Research (A*STAR) Singapore Singapore; ^5^ National University of Singapore (Suzhou) Research Institute Suzhou Jiangsu China

**Keywords:** adsorption, carbon, catalysis, charge density, chemistry, faraday efficiency, partial charge, partial current, selectivity

## Abstract

Precise atomic coordination editing of single‐atom catalysts (SACs) provides an effective strategy to tune their electronic structures and catalytic selectivity. Yet, achieving near‐unity selectivity inversion between two competing reactions, allowing deliberate control over the preferred pathway, remains a significant challenge. Here, we demonstrate that single‐atom coordination editing of NiN_4_‐based SACs enables precise control over reaction selectivity, allowing a near‐complete switch between CO_2_ reduction (CO_2_RR) and H_2_ evolution (HER). While the symmetrically coordinated NiN_4_ motif preferentially stabilizes ^*^H over ^*^COOH, resulting in exclusive HER, replacing a nitrogen coordination atom in the NiN_4_ site with carbon (NiN_3_C) breaks the structural symmetry, upshifts the *d*‐band center, and polarizes the charge distribution, thus lowering the ^*^COOH activation barrier and favoring CO_2_‐to‐CO conversion. Guided by these theoretical insights, the corresponding catalysts were synthesized and verified by multiple characterization techniques. Unlike NiN_4,_ which exhibits exclusively HER‐dominated behavior, NiN_3_C achieves ∼99% CO Faradaic efficiency across a wide pH range, a partial current density of ∼840 mA cm^−2^, and a carbon energy efficiency of 77%. Notably, a turnover frequency of 6.03 × 10^5^ h^−1^ and > 100 h stability at industrial‐level currents surpass previously reported benchmarks. In situ ATR‐SEIRAS and charge‐density analysis revealed that the NiN_3_C structure weakens Ni‐centered σ interactions while enhancing C‐center π coupling with ^*^COOH, thereby shifting ^*^COOH adsorption from the Ni center to an adjacent C site, enabling CO_2_RR selectivity. This study establishes an atomic coordination‐editing strategy that provides mechanistic insight into catalytic pathway switching and enables high‐performance electrocatalysis toward desired products.

## Graphical abstract

Atomic coordination editing functions as an on/off switch that toggles single‐atom catalysts between CO_2_ electroreduction and hydrogen evolution.

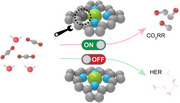

## 1 Introduction

The electrochemical CO_2_ reduction reaction (CO_2_RR) has emerged as a promising route to convert CO_2_ into value‐added fuels and chemicals by utilizing renewable electricity, thereby realizing the artificial carbon cycle and promoting the process toward carbon neutrality [1, 2, 3, 4]. However, CO_2_RR inevitably competes with the hydrogen evolution reaction (HER), because both reactions involve similar proton‐electron transfer steps and overlapping potential ranges. Achieving high reaction selectivity in electrochemical CO_2_RR, particularly toward a single CO_2_ reduction pathway while effectively suppressing HER, remains one of the grand challenges in electrocatalysis. Rather than focusing on the diversity of CO_2_ reduction products, increasing attention has therefore been directed toward pathway‐level selectivity and product exclusivity, which are essential for establishing well‐defined and controllable reaction outputs [5, 6]. In this regard, the CO_2_‐to‐CO pathway represents a prototypical model system, as it can proceed with near‐unity selectivity. Such selective CO_2_‐to‐CO conversion provides a streamlined molecular output that can be integrated into established syngas‐based chemical manufacturing routes such as Fischer–Tropsch synthesis, methanol production, and hydroformylation [7, 8]. Nevertheless, developing simple and efficient electrocatalysts that deliver near‐unity selectivity and Faradaic efficiency (FE) for CO_2_ reduction is highly desired.

Nickel‐based single‐atom catalysts (SACs), with a square‐planar NiN_4_ configuration recognized as the most representative active motif, have demonstrated high activity and selectivity in CO_2_RR at relatively low current densities [9, 10, 11, 12]. However, the saturated Ni^2+^ *d* ^8^ configuration and symmetric electron distribution in the NiN_4_ moiety lead to relatively weak interactions with the key ^*^COOH intermediate, which significantly hinders CO_2_ activation and reduces CO selectivity, particularly at high current densities [13, 14]. To overcome the intrinsic symmetry‐induced limitations, considerable efforts have been devoted to tuning the coordination and electronic structures of NiN_4_ sites through symmetry‐breaking strategies. Reducing coordination numbers to form unsaturated NiN_3_ or NiN_2_ configurations effectively breaks the local D_4h_ symmetry, introduces unoccupied 3*d* orbitals, and enhances ^*^COOH adsorption, thereby facilitating CO_2_ activation [14, 15, 16, 17, 18, 19]. Conversely, increasing coordination numbers or incorporating axial ligands, such as halogens, can modulate the out‐of‐plane electronic field and regulate interfacial charge redistribution for further promoting CO_2_‐to‐CO conversion [20, 21]. In addition, tailoring the outer coordination environment, through substrate engineering or long‐range π‐electron delocalization, has been shown to shift the Ni *d*‐band center and optimize intermediate binding, which enhances their catalytic performance [13, 22, 23]. Moreover, heteroatom doping (such as O, S, or P) can directly distort the planar symmetry of the MN_4_ coordination environment and alter the spin and charge distribution of the metal center, further improving CO_2_RR activity [18, 24, 25]. Although the aforementioned strategies have effectively enhanced CO_2_RR activity, they are often limited by structural instability and pH sensitivity. More importantly, they still lack a fundamental understanding of how the atomic‐level coordination environment determines catalytic behavior, highlighting the need for a more intrinsic and robust symmetry‐breaking design.

In this work, we demonstrated near‐unity electrocatalytic selectivity toggling between CO_2_RR and HER through atomic coordination editing of NiN_4_‐based SACs. We first employed density functional theory (DFT) calculations to construct a series of NiN_4_ and NiN_3_C coordination models, differing by a single N‐to‐C substitution, to elucidate how atomic coordination editing governs their catalytic activity and selectivity. In contrast to NiN_4_ sites, NiN_3_C sites with structural symmetry breaking possess a more optimized charge distribution and lower ^*^COOH energy barrier than NiN_4_ sites, thereby facilitating CO_2_ activation while suppressing HER. Compared with previous studies that mainly interpreted NiN_3_C coordination in terms of enhanced CO_2_RR performance [26, 27, 28], our work reveals a near‐complete switch between HER and CO_2_RR within the same Ni SAC platform, enabled by atomic coordination editing. Building upon these theoretical insights, a molecular‐to‐atomic strategy was employed, in which Ni(II) meso‐tetraphenyl‐porphyrin (TPPNi) was initially immobilized on conductive carbon nanotubes (denoted as Ni‐CNT), and then followed by controlled thermal treatment to alter the local coordination of Ni sites (Ni‐CNT‐600, where 600 refers to the heating temperature in °C). Multi‐technique characterizations, including aberration‐corrected high‐angle annular dark‐field scanning transmission electron microscopy (AC HAADF‐STEM) and X‐ray absorption fine structure (XAFS) spectroscopy, confirmed atomically dispersed Ni centers and a coordination evolution from well‐defined NiN_4_ to asymmetric, graphitized NiN_3_C structures. Electrochemical measurements further demonstrated a distinct catalytic switching behavior: Ni‐CNT‐600 exhibited nearly exclusive CO_2_‐to‐CO conversion, whereas Ni‐CNT showed HER‐dominated performance (>99.9% FE). As a result, Ni‐CNT‐600 achieved nearly 100% FE for CO production, a maximum partial current density of ∼840 mA cm^−2^, exceptional pH tolerance, and long‐term stability with 77% carbon energy efficiency (CEE). Notably, a superior turnover frequency (TOF) of 6.03 × 10^5^ h^−1^ at 600 mA cm^−2^ was obtained, outperforming state‐of‐the‐art Ni‐based SACs systems. In situ attenuated total reflection surface‐enhanced infrared absorption spectroscopy (ATR‐SEIRAS) combined with DFT analysis revealed that the C‐sites of NiN_3_C facilitated directional orbital coupling with ^*^COOH intermediates, accounting for the exceptional CO_2_RR performance. Extending this concept to other transition metal‐based (MN_4_) systems further verified the universality of atomic coordination editing, offering mechanistic insights and a general design principle for constructing asymmetric SACs.

## 2 Results and Discussion

**FIGURE 1:**
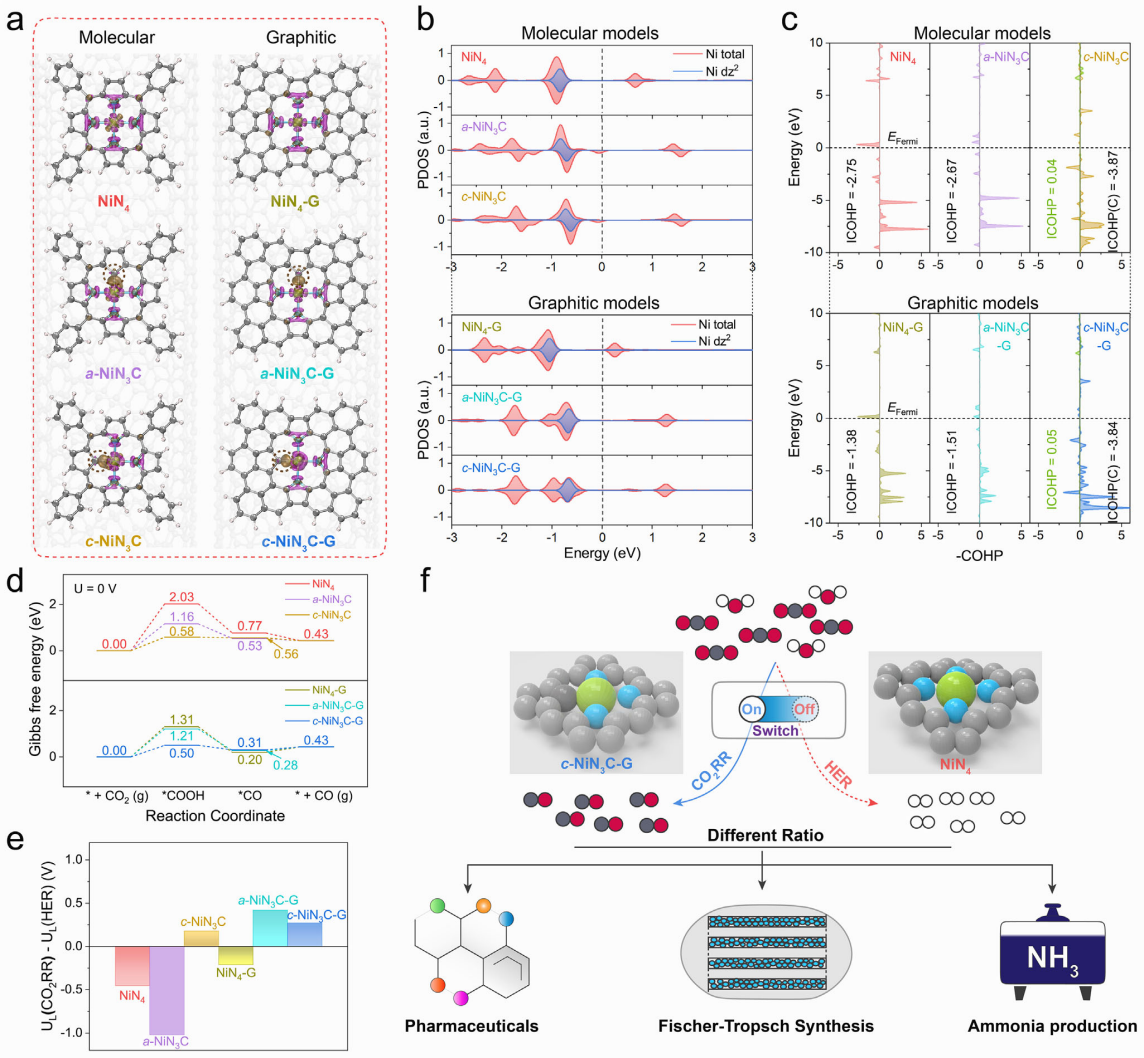
Computational evaluation of Ni‐based SACs containing both symmetric and asymmetric active sites. (a) Optimized structural models and corresponding charge density distributions of molecular (NiN_4_, *a*‐NiN_3_C and *c*‐NiN_3_C) and graphitic models (NiN_4_‐G, *a*‐NiN_3_C‐G and *c*‐NiN_3_C‐G) supported on CNTs; (b) PDOS diagrams for Ni *3d* orbitals of molecular and graphitic Ni single‐atom sites, with the dashed line indicating the Fermi level; (c) Crystal Orbital Hamilton Population (COHP) analysis for ^*^COOH adsorbed on molecular and graphitic Ni single‐atom sites; (d) Gibbs free energy diagram for CO_2_‐to‐CO conversion at U = 0 V on different Ni single‐atom configurations; (e) Comparison of limiting potential difference (U_L_(CO_2_RR)−U_L_(HER)) among various Ni–N–C structures, identifying HER‐ and CO_2_RR‐dominated regimes; (f) Schematic illustration of the “on‐off‐switch” applications for precisely regulating the ratios of CO and H_2_.

We first calculated the adsorption energies of ^*^CO_2_, ^*^COOH, and ^*^CO over a set of single‐atom Ni‐based coordination environments, including NiN_4_, NiN_3_, NiN_5_, and NiN_3_C (Figure S1). According to the Sabatier principle, optimal CO_2_ reduction requires balanced adsorption of key intermediates. Whereas altering the N‐coordination number alone introduces unavoidable adsorption trade‐offs among ^*^CO_2_, ^*^COOH, and ^*^CO, the NiN_3_C motif with symmetry breaking uniquely achieves a thermodynamically balanced adsorption energy profile, enabling favorable energetics for all intermediates. Inspired by this, we then designed a series of molecular and graphitic Ni single‐atom models with NiN_4_‐based (NiN_4_, NiN_4_‐G) and NiN_3_C‐based (*a*‐NiN_3_C, *c*‐NiN_3_C, *a*‐NiN_3_C‐G, *c*‐NiN_3_C‐G) motifs on carbon nanotubes, to establish how precise coordination editing of Ni‐based SACs governs catalytic performance (Figure S2). All Ni‐based SAC models were initially constructed and optimized on the CNT surface with an experimentally determined curvature of ∼20 nm diameter (Figure S3). To account for the support curvature effect, during the subsequent optimization of adsorption configurations of reactant and intermediates, the edge hydrogen atoms of the molecular phthalocyanine ligands were constrained [29]. Replacing a nitrogen coordination atom of NiN_4_ along the axial direction of the CNT creates two structures labeled as *a*‐NiN_3_C and *a*‐NiN_3_C‐G, whereas replacing a nitrogen atom along the circumferential direction produces two structures labeled as *c*‐NiN_3_C and *c*‐NiN_3_C‐G (Figure 1a). Such atomic substitution introduces both structural and electronic asymmetry at the Ni active sites, thus modulating their catalytic properties. Specifically, the NiN_4_ and NiN_4_‐G structures exhibit centrosymmetric Ni─N bonding and charge density distribution, which are unfavorable for the adsorption and activation of ^*^COOH intermediates at the Ni sites. In contrast, pronounced charge polarization develops along the Ni─C axis in all NiN_3_C configurations, driven by symmetry‐breaking‐induced electronic rearrangement. The corresponding graphitic structures (*a*‐NiN_3_C‐G and *c*‐NiN_3_C‐G) further enhance this polarization through π‐delocalization, thus promoting CO_2_RR activity.

To gain insight into the coordination‐regulation‐dependent electronic structures of different Ni‐N‐C motifs, the Ni *3d* partial density of states (PDOS) was analyzed to examine the variations near the Fermi level (*E_F_*). As depicted in Figure 1b and Figure S4, NiN_4_ and NiN_4_‐G models exhibit deeply located *d*‐band centers (−2.32 and −2.19 eV, respectively) with weak Ni 3dz^2^ contributions near *E_F_*. This is consistent with a nearly saturated electronic structure that limits interaction with the antibonding π^*^ orbitals of CO_2_ [30]. In contrast, the NiN_3_C‐based models display a pronounced upshift of the Ni 3dz^2^ states toward the *E_F_* (−0.80 eV for *c*‐NiN_3_C and −1.42 eV for *c*‐NiN_3_C‐G), accompanied by a broadening of the *3d* bands, indicative of enhanced orbital hybridization and charge delocalization. Such an electronic modulation strengthens CO_2_ adsorption and facilitates subsequent CO_2_ activation, accounting for the improved electrocatalytic activity.

Given that ^*^COOH is the key intermediate in CO_2_‐to‐CO conversion, understanding its interaction with the active site is essential for optimizing catalytic performance. Accordingly, crystal orbital Hamilton population (COHP) analysis was carried out to quantify the binding strength between ^*^COOH and active sites [31]. In Figure 1c, the integrated COHP (ICOHP) values for ^*^COOH adsorption followed the order: NiN_4_ (−2.75 eV) ≈ *a*‐NiN_3_C (−2.67 eV) > *c*‐NiN_3_C (−3.87 eV) in molecular systems, and NiN_4_‐G (−1.38 eV) > *a*‐NiN_3_C‐G (−1.51 eV) > *c*‐NiN_3_C‐G (−3.84 eV) in graphitic models. Among all structural models, *c*‐NiN_3_C and *c*‐NiN_3_C‐G exhibited the strongest ^*^COOH adsorption. This can be ascribed to the support‐induced strain at the NiN_3_C/CNT surface [29], which triggers local structural distortion and charge accumulation on the coordinated carbon atom, thereby reinforcing the C─^*^COOH bonding interaction [32].

To further clarify this effect from a thermodynamic perspective, Gibbs free‐energy profiles of the CO_2_RR pathway were calculated for NiN_4_ and NiN_3_C configurations. As shown in Figure 1d, the formation of ^*^COOH was identified as the rate‐determining step (RDS) in all systems. The corresponding free‐energy barriers decreased markedly with atomic coordination tuning: 2.03 eV (NiN_4_) > 1.16 eV (*a*‐NiN_3_C) > 0.58 eV (*c*‐NiN_3_C) in molecular systems, and 1.31 eV (NiN_4_‐G) > 1.21 eV (*a*‐NiN_3_C‐G) > 0.50 eV (*c*‐NiN_3_C‐G) in graphitic models, demonstrating more favorable ^*^COOH formation and enhanced CO_2_RR kinetics. Notably, *c*‐NiN_3_C and *c*‐NiN_3_C‐G structures showed the lowest energy barriers, consistent with the COHP‐predicted C─^*^COOH binding configuration. The limiting potential difference between CO_2_RR and HER (U_L_(CO_2_RR)−U_L_(HER)) was adopted as a descriptor for CO selectivity of these models (Figure 1e), where U_L_ = −ΔG_0_/e represents the limiting potential determined by the formation of intermediates ^*^COOH or ^*^H [33]. Since activation barriers scale linearly with binding energies, a more positive value of U_L_(CO_2_RR)−U_L_(HER) indicates a higher CO_2_RR catalytic selectivity [34]. Based on this analysis, *a*‐NiN_3_C‐G and *c*‐NiN_3_C‐G models exhibited more positive values compared to NiN_4_ and NiN_4_‐G catalysts, indicating enhanced CO selectivity. All these computational results demonstrated that atomic coordination editing modulated the Ni *3d* electronic states, upshifted the *d*‐band center, and optimized ^*^COOH adsorption, thereby switching the catalytic selectivity from HER to CO_2_RR (Figure 1f).

**FIGURE 2:**
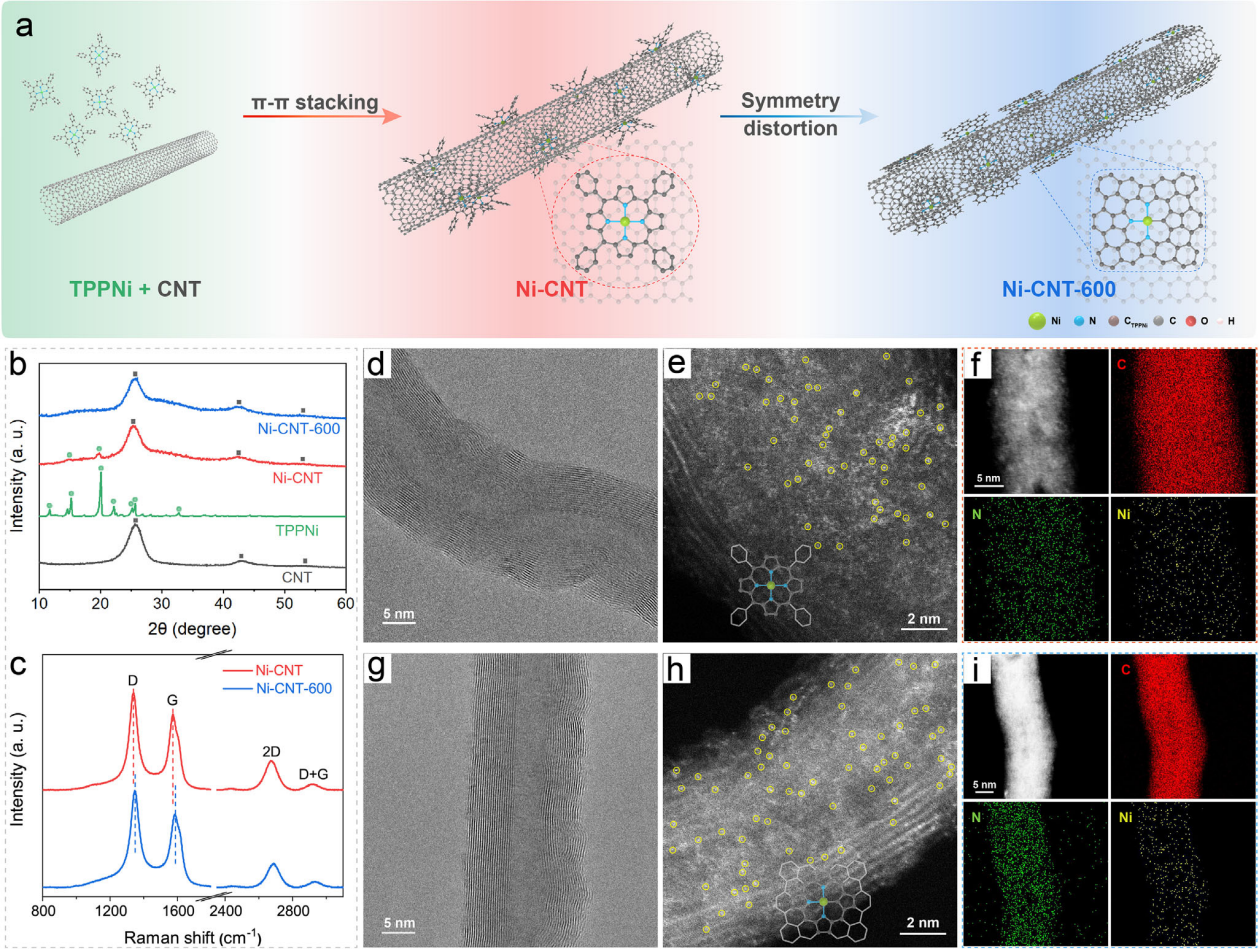
Synthesis and characterization of Ni‐CNT and Ni‐CNT‐600 samples. (a) Schematic diagram of synthesis for Ni‐CNT (symmetric NiN_4_ structure) and Ni‐CNT‐600 (asymmetric NiN_3_C structure) catalysts; (b) XRD patterns of the catalysts; (c) Raman spectra of Ni‐CNT and Ni‐CNT‐600; (d) TEM of Ni‐CNT; (e) HAADF‐STEM and (f) corresponding EDS mapping images of Ni‐CNT; (g) TEM of Ni‐CNT‐600; (h) HAADF‐STEM and (i) corresponding EDS mapping images of Ni‐CNT‐600.

Guided by these theoretical insights, a molecular‐to‐atomic evolution strategy was developed to construct the Ni–N–C sites. As illustrated in Figure 2a, TPPNi molecules were first immobilized onto CNTs through *π*–*π* stacking interactions [12], preserving a NiN_4_ coordination environment with high local symmetry (denoted as Ni‐CNT). The Ni‐CNT samples were then thermally treated under a controlled atmosphere, during which interfacial reactions occurred between the surface functional groups on CNTs (carbonyl/carboxyl/epoxy) and the porphyrin macrocycle [16, 35]. These reactions triggered partial carbonization and ligand rearrangement, resulting in local modification of the Ni coordination environment and the formation of graphitized NiN_3_C sites strongly anchored on the CNT surface (denoted as Ni‐CNT‐600). The XRD patterns (Figure 2b) revealed the disappearance of molecular diffraction peaks at 15.5° and 20.2° for Ni‐CNT‐600, indicating the loss of long‐range molecular order and structural distortion. The absence of any metallic or oxide peaks confirmed the atomic dispersion of Ni during thermally driven coordination conversion. The Raman spectra (Figure 2c) showed two peaks at 1346 and 1583 cm^−1^, which corresponded to *sp^3^* disordered carbon (D band) and *sp^2^* graphitic carbon (G band), respectively [36]. Ni‐CNT‐600 displayed an increased I_D_/I_G_ ratio from 1.247 to 1.318 and a suppressed 2D band, implying the generation of *sp^2^*‐lattice defects and reduced graphitic ordering. These defects likely stemmed from interfacial reactions between the porphyrinic macrocycle and carbon nanotubes [21], leading to the atomic coordination editing of NiN_4_ sites.

Scanning electron microscopy (SEM, Figures S5–S7) and transmission electron microscopy (TEM, Figure 2d,g) confirmed that both catalysts retained the multi‐walled nanotube morphology with an average diameter of approximately 20 nm. Ni‐CNT‐600 showed a roughened surface, which was attributed to defect formation and interfacial reconstruction. Aberration‐corrected high‐angle annular dark‐field scanning transmission electron microscopy (AC‐HAADF‐STEM) resolved isolated bright spots attributed to individual Ni atoms for both samples, with no observable Ni aggregation despite the different HAADF‐STEM contrast (Figure 2e,h). Moreover, Ni‐CNT‐600 demonstrated a more uniform dispersion of Ni atoms on the CNT surface, suggesting that the Ni–N–C framework was largely preserved during pyrolysis. The thermal treatment primarily induced local coordination rearrangement rather than aggregation or collapse of the atomic structure. Energy dispersive spectroscopy (EDS, Figure 2f,i) mapping revealed the uniform distribution of C, N, and Ni elements, while inductively coupled plasma‐optical emission Spectrometer (ICP‐OES, Table S1) yielded Ni loadings of 0.39 wt.% for Ni‐CNT and 0.42 wt.% for Ni‐CNT‐600, further validating atomic‐level preservation during the Ni single‐atom coordination tuning process.

**FIGURE 3:**
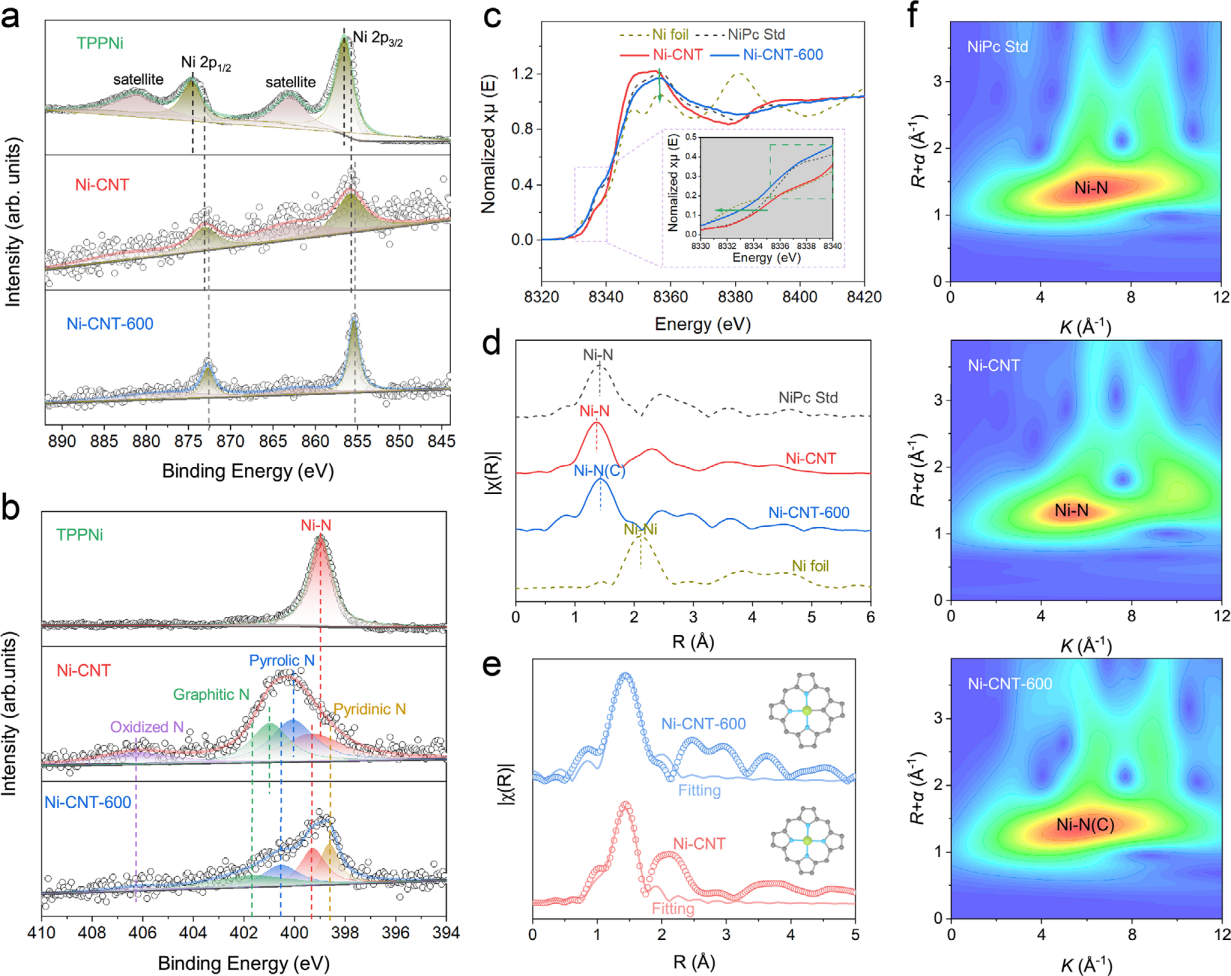
X‐ray photoelectron spectroscopy and X‐ray absorption spectroscopy analyses. (a) Ni 2p XPS spectra of TPPNi, Ni‐CNT, and Ni‐CNT‐600; (b) N 1s XPS spectra of CNT, TPPNi, Ni‐CNT, and Ni‐CNT‐600; (c) Ni K‐edge XANES spectra of Ni‐CNT and Ni‐CNT‐600 compared to standard NiPc and Ni foil. The inset shows the enlarged pre‐edge region; (d) The plotted Fourier transformation of the extended X‐ray absorption fine structure (FT‐EXAFS) spectra of Ni‐CNT, Ni‐CNT‐600, Ni foil, and NiPc; (e) Ni K‐edge EXAFS fitting results of Ni‐CNT and Ni‐CNT‐600, and the inset shows the proposed coordination configuration of the Ni center (Ni in green, N in blue, and C in gray); (f) Wavelet transform of the *k* ^3^‐weighted EXAFS data of NiPc, Ni‐CNT, and Ni‐CNT‐600.

To further verify the structural evolution, X‐ray photoelectron spectroscopy (XPS) and X‐ray absorption spectroscopy (XAS) were employed to probe the local electronic and coordination environment of the Ni sites (Figure 3). The Ni *2p* spectra displayed a progressive shift of the Ni *2p*_3/2_ peak from 856.3 eV in TPPNi to 855.8 eV in Ni‐CNT, and further to 855.4 eV in Ni‐CNT‐600, accompanied by attenuation of the satellite peak and a parallel shift of the Ni *2p*_1/2_ peak (874.0 to 873.0, to 872.7 eV, Figure 3a). The features at 856.3 and 874.0 eV indicate that Ni is in the +2 oxidation state [37]. The decrease in Ni *2p* binding energy suggests a gradual increase in electron density at the Ni centers during synthesis. Initially, TPPNi was anchored on CNTs through *π*–*π* interactions and was subsequently converted into an electron‐enriched Ni─N─C coordination environment by pyrolysis. The N *1s* spectra (Figure 3b) provide further evidence for this evolution. TPPNi exhibited a single Ni─N peak at 399.0 eV, characteristic of the well‐defined NiN_4_ coordination in its porphyrinic framework. After immobilization onto CNTs, four deconvoluted components appeared at 399.2 eV (Ni─N), 400.0 eV (pyrrolic N), 401.0 eV (graphitic N), and 406.3 eV (oxidized N) [38]. The slightly positive shift of the Ni‐N peak in Ni‐CNT arose from *π*–*π* interactions between the porphyrin macrocycle and the CNT support. Upon thermal treatment, Ni‐CNT‐600 exhibited a new pyridinic N signal at 398.5 eV and a reduced relative intensity of the Ni‐N component [39]. These changes suggested that the original NiN_4_ configuration underwent a partial N‐coordination dissociation, forming an asymmetric NiN_x_ (*x* < 4) environment. The corresponding C *1s* spectra (Figure S8) revealed a substantial decrease in oxygenated carbon species (C─O, O─C═O) and a slight upshift of the C–N component, confirming the conversion of the molecular porphyrin into robustly coupled Ni–N–C sites embedded in a conductive carbon matrix.

The Ni K‐edge X‐ray absorption near‐edge structure (XANES) spectra clearly demonstrated the electronic and coordination environment evolution of Ni centers in Ni‐CNT, Ni‐CNT‐600, Ni foil, and nickel phthalocyanine (NiPc) standard samples (Figure 3c). Ni‐CNT‐600 exhibited a distinct edge shift toward lower energy and a reduced white‐line intensity compared with Ni‐CNT, while TPPNi exhibited a higher‐energy absorption edge and a stronger white‐line intensity than Ni‐CNT (Figure S9). These results collectively indicate a gradual increase in Ni *3d* electron density from TPPNi to Ni‐CNT and then to Ni‐CNT‐600, consistent with the XPS results (Figure 3a). In the pre‐edge region (∼8334 eV, *1s*‐*3d* transition), Ni‐CNT overlapped with NiPc, consistent with a NiN_4_‐like coordination, whereas Ni‐CNT‐600 displayed a slightly broadened and shifted pre‐edge feature, suggesting enhanced *3d*‐*4p* hybridization and lowered coordination symmetry [15]. In the fingerprint region near 8337 eV (*1s*‐*4p_z_* transition), Ni‐CNT‐600 showed a similar feature to NiPc, indicative of a quasi‐planar coordination geometry of graphitic NiN_3_C sites (Figure 2a) [32]. Ni‐CNT displayed a weaker shoulder near 8336 eV, which might be assigned to NiN_4_ macrocycle out‐of‐plane distortion and *π*–*π* interactions with CNTs (Figure 2a).

In addition, the Ni K‐edge Fourier‐transformed *k* ^3^‐weighted extended X‐ray absorption fine structure (FT‐EXAFS) spectra of Ni‐CNT samples showed a main peak centered at 1.36 Å in R space (Figure 3d), corresponding to Ni–N scattering pathways in the first shell, indicating that a well‐defined NiN_4_ configuration is preserved. For Ni‐CNT‐600, the prominent peak shifted to 1.42 Å, suggesting pronounced coordination alteration and increased local distortion. In the second shell, Ni‐CNT‐600 also exhibited a longer Ni–C distance (2.47 Å relative to 2.30 Å for Ni‐CNT), coinciding with the NiPc reference, which implied weaker Ni–ligand covalency and enhanced electronic coupling after the thermal treatment. The absence of the Ni‐Ni fingerprint (∼2.1 Å) in both samples suggests that Ni atoms are atomically dispersed on the CNT framework. Furthermore, quantitative least‐squares EXAFS fitting was performed to elucidate the coordination of the Ni centers. As shown in Figure 3e, one Ni atom is coordinated with four first‐shell N atoms in Ni‐CNT, with an average bond length of 1.87 Å (Table S2). Ni‐CNT‐600 presented a different coordination structure, in which Ni bonded with approximately one first‐shell C atom and three first‐shell N atoms, with bond lengths of 1.87 and 1.93 Å (Table S2) [32]. To address the possible axial/circumferential assignment of NiN_3_C‐G, C‐substitution formation‐energy calculations were further performed, revealing that *c*‐NiN_3_C‐G has a markedly lower formation energy than *a*‐NiN_3_C‐G in the curved CNT model, thus supporting the circumferential configuration as the more plausible structure of Ni‐CNT‐600 (Figure S10). Moreover, computational analysis revealed that the Ni─C bond length (1.96 Å, Table S3) in the *c*‐NiN_3_C‐G configuration is comparable to the Ni─N bond length (1.96 Å) in the NiN_4_ motif, whereas the Ni─N bond distance (2.02 Å) in *c*‐NiN_3_C‐G increases. Additionally, as revealed in the wavelet‐transform (WT) of the Ni K‐edge EXAFS (Figure 3f), the intensity centroid in Ni‐CNT‐600 demonstrated a discernible positive shift compared to Ni‐CNT, suggesting an increase in the Ni─N(C) bond length owing to partial substitution of N atoms by C atoms. Combined with the XANES fitting analysis and additional electron energy‐loss spectrum (Figures S11 and S12), all these results support that thermal activation transformed the molecular out‐of‐plane NiN_4_ motif into a quasi‐planar asymmetric NiN_3_C structure, enabling stronger Ni‐C electronic coupling, higher 3*d* electron density, and optimized active‐site geometry for CO_2_ reduction.

**FIGURE 4:**
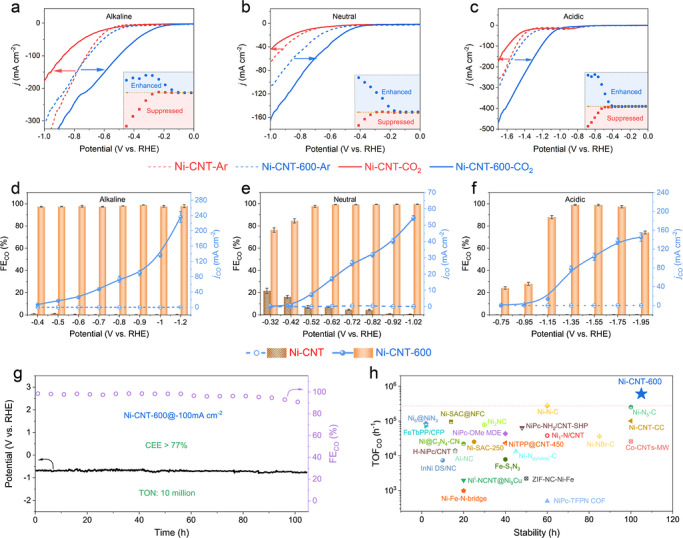
Electrochemical CO_2_ reduction performance in flow cells. (a–c) LSV curves of Ni‐CNT and Ni‐CNT‐600 in Ar or CO_2_ atmospheres with (a) alkaline solution (1 m KOH, pH = 13.8), (b) neutral solution (0.1 m KHCO_3_, pH = 8.2) and (c) acidic solution (0.05 m H_2_SO_4_ + 0.5 m K_2_SO_4_, pH = 1.0); (d–f) FE_CO_ and corresponding electrolysis currents for Ni‐CNT and Ni‐CNT‐600 at different applied potentials in (d) alkaline, (e) neutral and (f) acidic conditions; (g) Stability test at 100 mA cm^−2^ on Ni‐CNT‐600 in 1 m KOH; (h) TOF and stability of Ni‐CNT‐600 compared with those of other state‐of‐the‐art CO_2_‐to‐CO reduction catalysts (for detailed data see Table S4). The insets in (a–c) present the ratio of the current density under CO_2_ relative to that under Ar. The blue‐shaded region represents a CO_2_RR‐dominant regime, as exemplified by Ni‐CNT‐600, whereas the red‐shaded region represents a HER‐dominant regime, as observed for Ni‐CNT. Error bars in (d–f) represent the standard deviation of three independent measurements.

Next, we systematically investigated the electrochemical CO_2_ reduction performance of Ni‐CNT and Ni‐CNT‐600 in a flow cell system using a gas diffusion electrode (Figure 4). In addition, control experiments employing molecular TPPNi, pristine CNT, and their thermally treated analogues (TPPNi‐600 and CNT‐600) exhibited negligible CO_2_RR activity within the tested potential range (Figures S13–S16). The catalytic activity of a series of Ni‐CNT‐based samples pyrolyzed from room temperature up to 900°C (Figures 4; Figures S17–S25) revealed a distinct non‐monotonic dependence on temperature. Pristine Ni‐CNT with a NiN_4_ configuration resulted in negligible CO_2_RR activity (FE_CO_ < 0.1%) and HER‐dominated behavior (99.9% FE). Moderate pyrolysis at 300°C and 450°C induced partial coordination structural distortion, in which Ni‐CNT‐300 remained mostly HER‐active (FE_CO_ = 13%), and Ni‐CNT‐450 yielded a limited partial current density of ∼128 mA cm^−2^ with 95% FE_CO_. Further heating to 600°C, Ni‐CNT‐600 achieved nearly 100% FE_CO_ with a *J*_CO_ of ∼248 mA cm^−2^, implying a complete transformation to NiN_3_C coordination. At 750°C, however, pronounced Ni(I) signals emerged in ESR spectra (Figure S26) [11, 40], indicating over‐denitrogenation and excessive coordination distortion. The resulting electron‐rich Ni centers limited ^*^COOH adsorption, lowering CO_2_RR activity (FE_CO_ ∼94% and *J*_CO_ ∼119 mA cm^−2^). Upon heating to 900°C, the splitting of the ESR peak at *g* ≈ 2.38 indicated the collapse of the Ni‐N‐C framework to form aggregated Ni clusters, which is confirmed by the XANES and EXAFS results (Figure S27), with HER once again dominating.

Electrochemical measurements under CO_2_ and Ar atmospheres further revealed the coordination‐regulated catalytic pathways (Figure 4a–c). Ni‐CNT‐600 exhibited much lower onset potentials and higher currents in CO_2_ than in Ar across all pH values, indicating that NiN_3_C sites effectively activated CO_2_ while suppressing HER. In sharp contrast, Ni‐CNT displayed higher currents under Ar, suggesting that CO_2_ adsorption blocked proton‐reduction sites on the NiN_4_ active centers. The ratios of linear sweep voltammetry (LSV) currents between CO_2_ and Ar (Figure 4a–c inset) highlighted this contrast: CO_2_ acted as a reactive promoter on NiN_3_C sites but as a site blocker on NiN_4_ ones. Correspondingly, Ni‐CNT‐600 achieved superior CO_2_RR activity with a maximum FE_CO_ of 99% across a wide pH range (Figure 4d–f), whereas Ni‐CNT remained HER‐dominated. The slightly lower FE_CO_ of Ni‐CNT‐600 in acidic media (Figure 4f) mainly originated from kinetically favored proton reduction, consistent with its reduced current under CO_2_ atmosphere (Figure 4c). In addition, Ni‐CNT‐600 displayed much lower onset potentials than Ni‐CNT under a CO_2_ atmosphere, meaning superior CO_2_RR kinetics. The inverse electrochemical behaviors clearly manifested that the atomic coordination editing of NiN_4_ active sites switched the reaction pathway from proton‐dominated HER to ^*^COOH‐mediated CO_2_RR.

To explore whether the atomic coordination editing strategy could be extended beyond the Ni system, analogous M‐CNT and M‐CNT‐600 catalysts (M = Mn, Fe, Co, Cu, and Zn) were synthesized and tested (Figures S28–S49). All samples preserved the characteristic nanotube morphology with a uniform diameter of ∼20 nm, without detectable aggregation. After pyrolysis, the core level binding energies of other metals in M‐CNT‐600 samples shifted to lower values (Figures S38–S42), which can be attributed to enhanced electronic delocalization and disruption of symmetric MN_4_ configurations. XAS results (Figure S43) of representative Co‐ and Cu‐based analogues further support similar pyrolysis‐induced coordination/electronic evolution while retaining atomically dispersed M–N(C) sites without obvious M–M scattering. Each M‐CNT‐600 yielded higher FE_CO_ than its uncalcined counterpart (M‐CNT, Figures S44–S48), suggesting that coordination editing may contribute to enhancing CO_2_RR activity. Among these systems (Figure S49), Ni‐CNT‐600 displayed the most pronounced enhancement in both FE_CO_ and partial CO current density, highlighting the unique advantage of the optimized NiN_3_C coordination in driving superior CO_2_RR performance.

The practical applicability of Ni‐CNT‐600 was further examined under industrial‐level current densities in 1 m KOH electrolyte (Figure S50). The FE_CO_ remained above 90% from 100 to 800 mA cm^−2^ and reached 84 ± 1% even at 1 A cm^−2^, with the slight decline attributed to mass‐transport limitations at extremely high currents. Long‐term electrolysis maintained a stable FE_CO_ above 90% for over 105 h at 100 mA cm^−2^ (Figure 4g), in which more than 10 million turnover numbers and approximate 77% CEE were obtained. Post‐electrolysis HAADF‐STEM and XAS characterizations (Figures S51 and S52) further confirm that Ni‐CNT‐600 retains atomically dispersed Ni sites without detectable aggregation after CO_2_RR, indicating the high structural robustness of the asymmetric NiN_3_C configuration. Notably, an exceptional TOF of 6.03 × 10^5^ h^−1^ was achieved at 600 mA cm^−2^, surpassing state‐of‐the‐art Ni‐based SAC systems (Figure 4h; Table S4). When the turnover frequency (TOF_SAS_, h^−1^) was evaluated by the concentration of surface‐active sites, the corresponding TOF_SAS_ can reach 4.76 × 10^5^ h^−1^.

**FIGURE 5:**
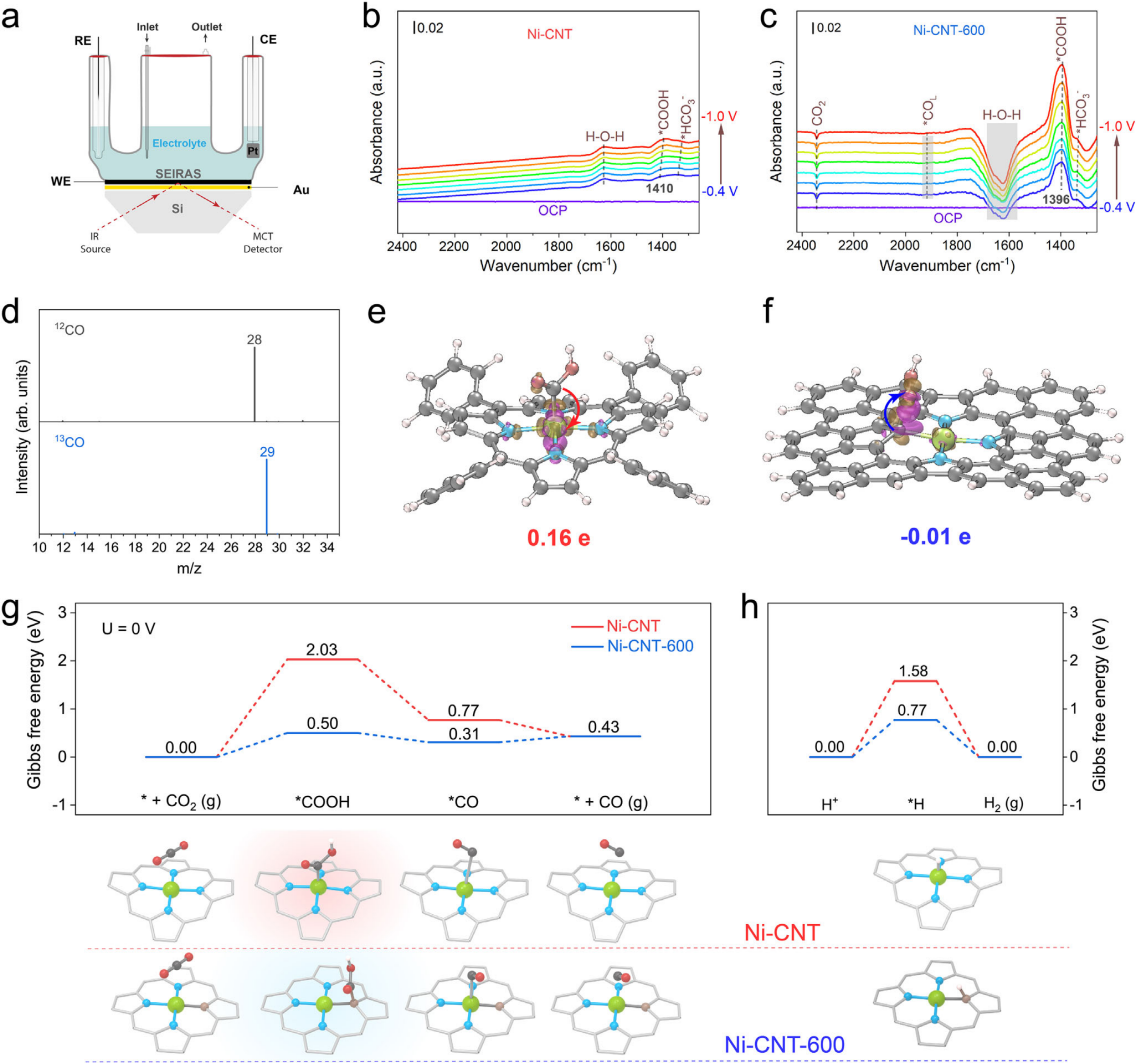
Probing mechanistic insights into selectivity inversion between CO_2_RR and HER. (a) Schematic diagram of in situ ATR‐SEIRAS setup; In situ ATR‐SEIRAS spectra of (b) Ni‐CNT and (c) Ni‐CNT‐600 acquired at potentials ranging from OCP to −1.0 V vs. RHE; (d) Mass spectra of CO product from ^12^C‐ and ^13^C‐labeled CO_2_ reduction processes for Ni‐CNT‐600; The charge density difference analysis between catalytic active sites and ^*^COOH intermediates for (e) Ni‐CNT and (f) Ni‐CNT‐600 catalysts (purple: electron density accumulation; brown: electron density depletion); The reaction pathways of (g) CO_2_RR and (h) HER over Ni‐CNT and Ni‐CNT‐600 (top: symmetric NiN_4_ sites with different intermediates; bottom: asymmetric NiN_3_C sites with different intermediates).

To clarify the intrinsic impact of coordination environment on CO_2_RR performance, temperature‐programmed desorption (TPD), in situ ATR‐SEIRAS, ^13^CO_2_ isotope labeling, and charge‐density analyses were employed (Figure 5; Figures S53–S62). CO_2_‐TPD and CO‐TPD results (Figure S53) revealed that Ni‐CNT‐600 displayed higher CO_2_ desorption and lower CO desorption temperatures than Ni‐CNT, indicating that the NiN_3_C sites indeed enhanced CO_2_ adsorption while facilitating CO release [41, 42, 43]. In situ ATR‐SEIRAS spectra revealed potential‐dependent evolution of surface intermediates on both Ni‐CNT and Ni‐CNT‐600 (Figure 5a–c; Figures S54 and S55). Distinct vibrational features appeared at ∼1240, ∼1398–1410, ∼1624, ∼1920 and 2343 cm^−1^, corresponding to HCO_3_^−^ stretching, C═O stretching of ^*^COOH, H─O─H bending, ^*^CO stretching, and ^*^CO_2_ stretching, respectively [7, 44, 45]. Upon applying the potential from −0.4 to −1.0 V, Ni‐CNT‐600 showed a clear potential‐dependent enhancement of the ^*^COOH signal (∼1398 cm^−1^), whereas Ni‐CNT displayed only weak ^*^COOH features (∼1410 cm^−1^). The linearly adsorbed ^*^CO feature at ∼1920 cm^−1^ and the CO_2_‐related signal at ∼2343 cm^−1^ showed relatively weak but resolvable potential‐dependent changes, as clarified by the enlarged spectra in Figure S54. These weak ^*^CO_L_ and CO_2_ signals are consistent with facile CO desorption and continuous CO_2_ feeding during the ATR‐SEIRAS measurement. These observations suggested that Ni‐CNT‐600 with NiN_3_C configuration effectively stabilized ^*^COOH intermediates and facilitated ^*^CO formation. The red‐shift of ^*^COOH vibration from 1410 cm^−1^ (Ni‐CNT) to 1398 cm^−1^ (Ni‐CNT‐600) is likely due to a transition of adsorption sites between ^*^Ni–COOH and ^*^C–COOH, consistent with ICOHP analysis (Figure 1c). The weak ^*^CO signals on Ni‐CNT‐600 represented a facile CO desorption behavior, in agreement with its lower CO‐TPD temperature. Broader O─H stretching bands (3200–3600 cm^−1^, Figure S55) suggested enhanced interfacial water activation that promotes proton‐coupled CO_2_ reduction. ^13^CO_2_‐labeling experiments (Figure 5d) further confirmed that CO (*m/z* = 29) originated exclusively from CO_2_ electroreduction, ruling out carbon contamination and verifying the inherent CO_2_‐to‐CO conversion on NiN_3_C sites.

Double‐layer capacitance (C*_dl_*) measurements (Figures S56 and S57) showed comparable electrochemically active surface areas (ECSA) for Ni‐CNT (20.2 mF cm^−2^) and Ni‐CNT‐600 (22.1 mF cm^−2^), but the latter exhibited three orders of magnitude higher CO_2_RR activity. This verified that their activity difference stems from site‐specific coordination editing rather than surface area. The analysis of charge density difference (Figure 5e,f) clarified their different ^*^COOH‐binding characteristics. In symmetric NiN_4_ models, ^*^COOH transferred excessive charge (∼0.16 e) to the Ni center, leading to over‐binding and sluggish reduction kinetics. The NiN_3_C configuration of Ni‐CNT‐600 displayed negligible charge transfer (∼0.01 e, reversed toward ^*^COOH), together with reduced Ni *3d* states and the emergence of adjacent C *2p* states near the Fermi level (Figure S4). These features indicate weakened Ni‐centered σ‐type interaction and enhanced π‐type coupling, shifting ^*^COOH adsorption to the adjacent asymmetric C‐site [32]. This optimized electronic coupling facilitated ^*^COOH activation and subsequent CO generation, in agreement with additional simulated models (Figures S58 and S59).

Our DFT calculations also revealed that the NiN_3_C configuration weakened Ni *d*‐orbital participation in ^*^COOH bonding, whereas the adjacent C atom dominated the ^*^COOH *p*‐orbital overlap, leading to stronger covalent interaction (Figures S60 and S61). Consequently, ^*^COOH preferentially bonded to the neighboring C‐site rather than the Ni center, forming a more stable C–^*^COOH configuration (*E_ad_* = −2.16 eV, Figure S62), which promotes CO_2_ activation. Free‐energy profiles and proposed mechanisms (Figure 5g; Figure S63) further confirmed that ^*^COOH formation remained the RDS for both catalysts, but the barriers decreased dramatically from 2.03 eV for Ni‐CNT to 0.50 eV for Ni‐CNT‐600. Although Ni‐CNT‐600 showed a lower HER barrier (0.77 eV, Figure 5h) than Ni‐CNT, it was still higher than that of ^*^COOH formation, resulting in CO_2_RR‐dominated activity. Conversely, Ni‐CNT demonstrated a lower HER barrier (1.58 eV) than ^*^COOH formation (2.03 eV), leading to HER‐preferred behavior. At U = −0.7 V (Figure S64), Ni‐CNT‐600 maintained lower barriers for ^*^COOH formation and ^*^CO desorption, accounting for its superior CO_2_RR activity. Overall, the transformation from NiN_4_ to NiN_3_C reorganized the electronic configuration and reaction energetics, optimized ^*^COOH binding, and switched the catalytic pathway from HER to CO_2_RR. Furthermore, by modulating the ratio of Ni‐CNT and Ni‐CNT‐600 catalysts (Figure S65), the H_2_/CO product ratio was precisely tuned from 1:2 to 4:1, enabling the design of tailored syngas compositions for applications ranging from CO‐rich chemical synthesis to H_2_‐rich energy production.

## 3 Conclusions

In summary, we demonstrated that atomic coordination editing of NiN_4_ active sites acts as an atomic‐scale switch to precisely regulate the selectivity between HER and CO_2_RR on Ni‐based SACs. Guided by DFT predictions and confirmed experimentally, converting symmetric NiN_4_ into asymmetric NiN_3_C breaks the structural symmetry, upshifts the *d*‐band center, and polarizes the charge distribution. Consequently, this reorients ^*^COOH adsorption from the Ni‐site to an adjacent C‐site and lowers the ^*^COOH formation barrier below that of HER over NiN_3_C sites, thereby reversing the catalytic selectivity toward CO_2_RR. The optimized catalysts (Ni‐CNT‐600) with asymmetric NiN_3_C sites achieved ∼99% FE_CO_ across a wide pH range, ampere‐level current densities, > 100 h stability, and 6.03 × 10^5^ h^−1^ TOF, far exceeding their symmetric counterpart. The generality of this coordination‐editing strategy was further validated across multiple transition‐metal SAC systems. These findings establish a clear mechanistic link among atomic coordination editing, electronic configurations, and reaction energetics, providing a rational design principle for SACs that enables deliberate control over catalytic pathways toward desired products.

## Author Contributions

Y.Z., Y.H., and M.D. contributed equally. The manuscript was written through the contributions of all authors. All authors have given approval to the final version of the manuscript.

## Conflicts of Interest

The authors declare no conflicts of interest.

## Supporting information




**Supporting File**: advs75911‐sup‐0001‐SuppMat.docx.

## Data Availability

The data that support the findings of this study are available from the corresponding author upon reasonable request.
