# Supplementary material for: Near‐Unity Selectivity Inversion Between CO2 Electroreduction and H2 Evolution via Atomic Coordination Editing
Source: Adv Sci (Weinh). 2026 Jun 1:e75911. Online ahead of print. doi: 10.1002/advs.75911 (PMC13336911; doi:10.1002/advs.75911)
Supplement: Supplementary file 1 — Supporting File: advs75911‐sup‐0001‐SuppMat.docx. [file ADVS-9999-e75911-s001.docx]

Near-Unity Selectivity Inversion Between CO_2_ Electroreduction and H_2_ Evolution *via* Atomic Coordination Editing

*Yukun Zhao,^‡^ Yuanyuan He,^‡^ Mengyu Duan,^‡^ Peng Yin, Jinxing Chen, Xu Han, Jie Yang, Yulin Wang, Xingjie Fu, Shibo Xi, Chuncheng Chen,* and Jiong Lu**

**Experiment Section:**

**Chemicals:** TPPNi, TPPFe, TPPCo, TPPCu, TPPZn and TPP ligands were obtained from Aladdin. Multi-walled carbon nanotubes (8-15 nm) were purchased from Tanfeng. Nitric acid (HNO_3_, 69 wt.%) and sulfuric acid (H_2_SO_4_, 98 wt.%) were purchased from Honeywell Specialty Chemicals Seelze GmbH. Dimethylformamide (DMF) was purchased from VWR Chemicals. Potassium hydroxide (KOH), potassium bicarbonate (KHCO_3_, 99.7%), potassium sulfate (K_2_SO_4_, 99%) and Nafion perfluorinated resin solution (5 wt.%) were purchased from Sigma-Aldrich. All chemicals were used as received without further purification. Deionized (DI) water was used throughout this study.

**Preparation of Ni-CNT and Ni-CNT-600:** Carbon nanotubes (CNTs, 1g) were treated with 100 mL HNO_3_ at 100 ^o^C, under stirring and refluxing for 4 hours. Then samples were washed with DI water (neutral) and dried at 80 ^o^C for overnight in a vacuum oven. 40 mg obtained CNTs and 4 mg TPPNi were dispersed into 40 mL and 10 mL DMF solutions, respectively. Both suspensions were sonicated for 1 h and then combined and further sonicated for an additional 1 h. The obtained solution was stirred overnight. Ni-CNT samples were collected by rotary evaporation and dried at 80 ^o^C. Ni-CNT samples were further carbonized at 600 ^o^C for 2 h under a 5% H_2_/Ar atmosphere (progress temperature rate: 2 ^o^C min^-1^). The obtained sample was denoted as Ni-CNT-600. Other Ni-CNT-X samples were prepared following the same procedure, except that the calcination temperatures were varied (300, 450, 750, and 900 ^o^C).

**Preparation of M-CNT and M-CNT-600:** M-CNT and M-CNT-600 were synthesized using the same procedure as that for Ni-CNT and Ni-CNT-600, except that TPPM (M = Mn, Fe, Co, Cu or Zn) was used instead of TPPNi.

**Characterization.** The powder XRD patterns were recorded using a Brucker D8 advance X-ray diffractometer (Cu Kα radiation, λ = 1.54 Å). Raman spectra were recorded on a Laser Micro-Raman Spectrometer (Renishaw) with 532 nm excitation wavelengths. SEM and TEM images were collected with an SU8010 (Hitachi, Japan) and HT770 (Hitachi, Japan), respectively. AC-HAADF STEM images were acquired using a JEOL ARM200F microscope equipped with a probe-forming spherical aberration corrector. XPS spectra were collected on an ESCALAB 250Xi spectrometer (300 W Al-Kα radiation), and all binding energies were calibrated against the C 1s peak at 284.8 eV. XANES and EXAFS measurements were performed at the XAFCA beamline of the Singapore Synchrotron Light Source and analyzed using standard procedures implemented in the IFEFFIT package. The metal loading of all samples was determined by ICP-OES. CO_2_-TPD and CO-TPD measurements were conducted using a chemisorption analyzer. ESR signals were recorded on an ESR spectrometer (ELEXSYS E500, Brucker).

**Electrochemical measurements.** 1 mg of the catalyst was dispersed in a solution of ethanol (1.95 mL) and 5 *wt.*% Nafion solution (50 μL), followed by sonication for 1 h. The obtained catalyst dispersion (100 μL) was drop-cost onto a GDL (Sinero YLS-30T, 0.5 × 2.0 cm^2^) and used as the cathode. The CO_2_RR measurement (CHI 760E) was performed in a flow cell separated by a Nafion 117 membrane (activation for 24 h before use), with a regularly checked Ag/AgCl reference electrode (replaced when necessary) and Pt foil as the counter electrode. The electrolytes (1M KOH, 0.1 M KHCO_3_, or 0.05M H_2_SO_4_ + 0.5 M K_2_SO_4_) were circulated at a flow rate of 10 mL min^-1^, while CO_2_ gas was supplied at a flow rate of 40 sccm. All potentials were converted to the RHE according to the following equation: *E (vs. RHE) = E (vs. Ag/AgCl) + 0.197 V + 0.0591× pH*. The gaseous products were detected online using Shimadzu GC-2020 gas chromatograph equipped with a BID detector. The liquid products were quantified by *^1^H* NMR (Bruker ADVANCE III, 400 MHz) using DMSO as an internal standard.

The Faradaic efficiency (FE) of H_2_ and CO were calculated by the following equation:

$FE=\frac{n\times m\times F}{I\times t}\times100$*(1)*

The turnover frequency (TOF, h^-1^) was calculated by the following equation:

$TOF=\frac{\frac{j_{CO}}{nF}}{m_{cat}\times\frac{\omega}{M_{metal}}}\times3600$ *(2)*

The turnover frequency (TOF_SAS_, h^-1^) based on the concentration of surface-active sites was evaluated according to the following equation:

${TOF}_{SAS}=\frac{n_{CO}}{n_{Ni}}=\frac{j_{CO}}{2F\times\tau_{0}}\times3600$ *(3)*

$\tau_{0}$(surface concentration, mol cm^-2^) can be evaluated by following equation:

$Slope=\frac{n^{2}F^{2}\tau_{0}}{4RT}$*(4)*

where n = 1, F is Faraday constant, R=8.3145 J mol^-1^ K^-1^, T is the temperature (298.15 K), and the slope of Ni-CNT-600 is 22.1 mF cm^-2^.

The carbon energy efficiency (CEE) was calculated by the following equation:

$CEE=\frac{{(1.23-E}_{CO})\times{FE}_{CO}}{{-E}_{Cell}}$ *(5)*

*I* is the electrolysis current (A);

*t* is the electrolysis time (s);

*n* is the number of electrons transferred for CO (or H_2_), *n* = 2;

*m* is the molar amount of the products (mol);

*F* is the Faradaic constant (96485 C mol^-1^);

*j_CO_* is the partial current for CO (A);

*m_cat_* is the catalyst mass in the electrode (0.25 mg);

*ω* is the Ni content in the catalyst determined by ICP-OES;

*M_metal_* is the atomic mass of Ni (58.69 g·mol^-1^);

*E_CO_* is the thermodynamic potential for CO formation;

*E_Cell_* is the full-cell voltage measured in the slim flow cell.

**In situ ATR-SEIRAS measurements.** Electrochemical in situ ATR-SEIRAS spectra were collected on a Bruker Vertex 70V spectrometer equipped with a narrow-band HgCdTe detector, in which the whole process was protected by liquid nitrogen. As shown in Figure 5a, the gold-plated Si prism coated with the catalyst (employed as the working electrode) was integrated into a homemade ATR reflection cell, equipped with a platinum wire counter electrode and an Ag/AgCl reference electrode. All infrared spectra acquisitions were recorded in a CO_2_-saturated electrolyte containing 0.1M KHCO_3_. The background spectrum was recorded under open-circuit conditions prior to each electrochemical reduction measurement. The applied voltage was then scanned from 0 to -1.0 V vs. RHE at a rate of 5 mV s^-1^, with spectra collected at a resolution of 4 cm^-1^ using a spectrometer operating at 160 kHz.

**Computational Methods.** Spin-polarized geometric optimizations and thermodynamic corrections were performed using density functional theory (DFT) as implemented in the *Vienna Ab initio Simulation Package* (VASP). The generalized gradient approximation (GGA) in the form of the Perdew-Burke-Ernzerhof functional (PBE) was adopted to describe the exchange-correlation interactions. A cutoff energy is 450 eV for plain-wave basis sets, and the convergence threshold are 1×10^-5^ eV for energy and 0.01 eV/Å for force, respectively. The van der Waals interaction was calculated by the DFT+D3 method using empirical correction in Grimme’s scheme57. The atomic coordinates of optimized Nickel-based single atom catalyst models (NiSACs; Figures S1-S3) are provided in Supplementary Information. The models were bent to match the curvature of CNTs with a diameter of approximately 20 nm. To prevent the imposed curvature, the edge hydrogen atoms of the phthalocyanine ligand were constrained in geometry optimization. All VASP optimizations were in a 29×29×29 Å^3^ box with a 1×1×1 *k*-point Monkhorst–Pack grid.

The adsorption energy (*E*_ad_) of COOH^*^ and CO^*^ on NiSAC surface was calculated as:

$E_{ad}\left( {COOH}^{*} \right)=E_{{COOH}^{*}}-E_{NiSAC}-E_{COOH}$ (4)

$E_{ad}\left( {CO}^{*} \right)=E_{{CO}^{*}}-E_{NiSAC}-E_{CO}$ (5)

where $E_{{COOH}^{*}}$, $E_{{CO}^{*}}$, and $E_{NiSAC}$ are the DFT total energies of the catalyst with and without COOH and CO adsorption, respectively. $E_{COOH}$ and $E_{CO}$ are the isolated DFT energies of COOH and CO.

The *d*-band center (*ε_d_*) of the Ni was calculated as:

$\varepsilon_{d}=\frac{\int_{-\infty}^{E_{f}} E\times f(E)dE}{\int_{-\infty}^{E_{f}} f(E)dE}$ (6)

where *f(E)* is the density of states and *E_f_* is the energy of the Fermi level.

The Gibbs free energy (*G*) for each species can be given by:

$G=E_{DFT}+E_{ZPE}-TS.$ (7)

where $E_{DFT}$, $E_{ZPE}$, and *S* represent the DFT total energy, the zero-point energy, and the entropy at 298.15 K, respectively.

The relative free energy profiles for the CO_2_RR were calculated by:

$\Delta G_{COOH}=G_{COOH}-G_{{CO}_{2}}-\mu_{H}$ (8)

$\Delta G_{{CO}^{*}}=G_{{CO}^{*}}-G_{{CO}_{2}}+\mu_{H_{2}O}-{2\mu}_{H}$ (9)

$\Delta G_{CO}=G_{CO}-G_{{CO}^{*}}$ (10)

where $G_{COOH}$, $G_{CO2}$, and $G_{{CO}^{*}}$ represent the free energy of COOH, CO_2_, and CO adsorbed on NiSACs, respectively. $G_{CO}$ is the free energy of desorbed CO. $\mu_{H}$ and $\mu_{H_{2}O}$ are the free energies of one hydrogen atom in H_2_ and H_2_O under standard conditions, respectively. The above analyses were conducted using the post-processing program VASPKIT. The additional crystal orbital Hamilton populations (COHPs), projected density of states (PDOS), and natural adaptive orbitals (NAdOs) analyses were implemented in the CP2K simulation package.

The C-substitution formation energies (*E*_f_) of the axial and circumferential NiN_3_C-G configurations were calculated according to the following equation:

$E_{f}=E_{NiN_{3}C-G}-E_{NiN_{4}-G}+\mu_{N}-\mu_{C}$ (11)

where $E_{NiN_{3}C-G}$ and $E_{NiN_{4}-G}$ are the total energies of the curved NiN_3_C-G and NiN_4_-G models, respectively. $\mu_{N}$ and $\mu_{C}$ denote the chemical potentials of N and C atoms, referenced to gaseous N_2_ and solid graphite.


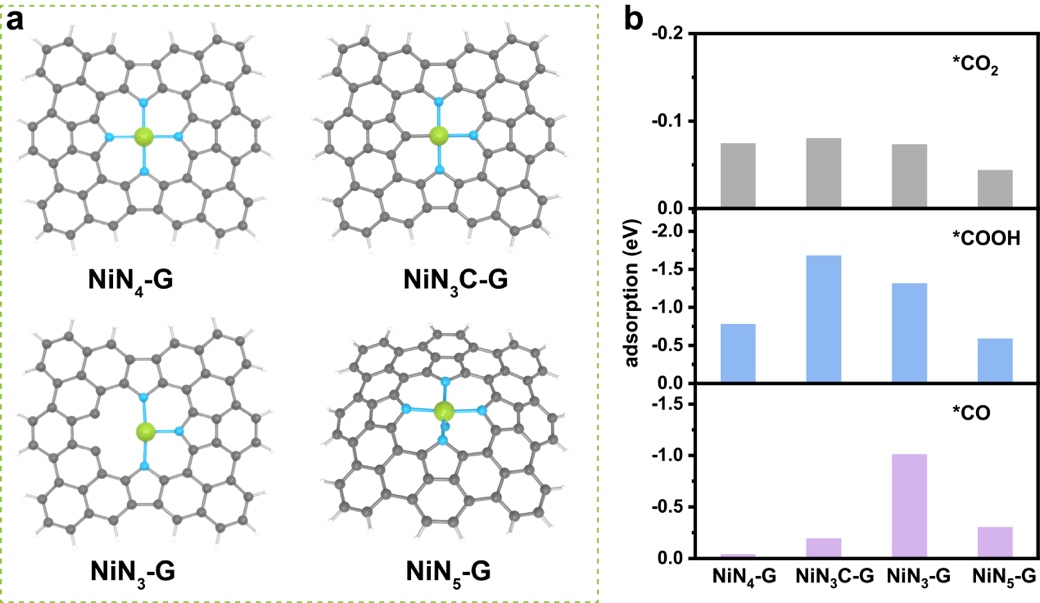


**Figure S1.** (a) Optimized geometric structures of Ni-centered coordination motifs including NiN_4_-G, NiN_3_C-G, NiN_3_-G, and NiN_5_-G. Ni atoms are shown in green, coordinated N atoms in blue, and C atoms in gray. (b) Calculated adsorption energies of *CO_2_ (top), *COOH (middle), and *CO (bottom) on the four Ni–N–C coordination environments.

A systematic comparison of intermediate adsorption energies reveals that simply altering the N coordination number can modulate binding strengths but also introduces intrinsic trade-offs. The NiN_3_ site, for instance, bind *CO too strongly, impeding product desorption, whereas NiN_5_ fails to sufficiently stabilize *COOH. In contrast, the NiN_3_C configuration achieves a more favorable balance between *COOH activation and *CO release, resulting in the most optimal theoretical adsorption profile. These results indicate that targeted N-to-C substitution to break the symmetry of NiN_4_ offers a rational strategy for promoting CO_2_RR performance.


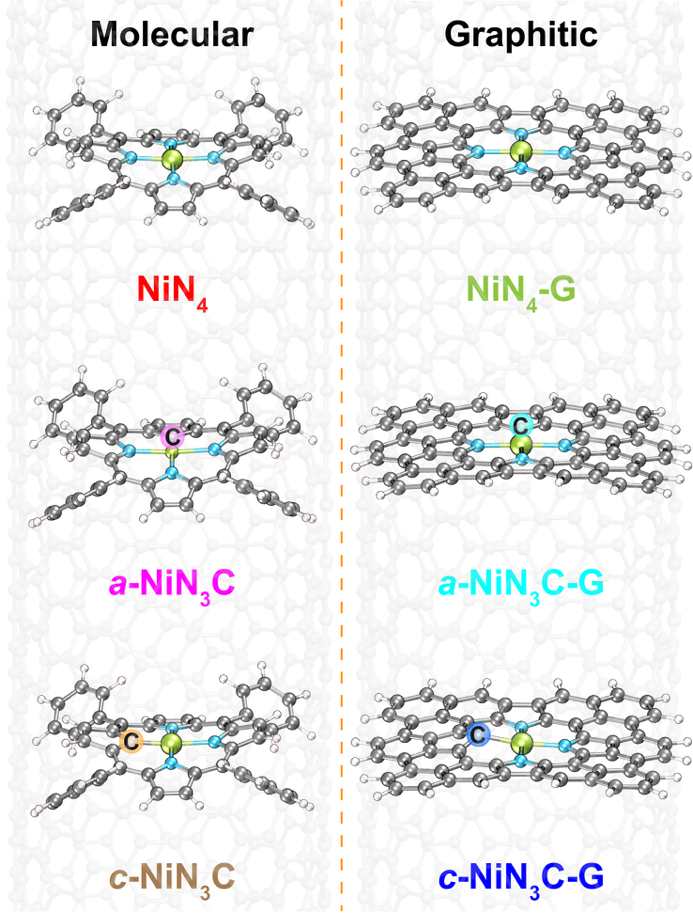


**Figure S2.** Optimized structural models of molecular (NiN_4_, *a*-NiN_3_C and *c*-NiN_3_C) and graphitic (NiN_4_-G, *a*-NiN_3_C-G and *c*-NiN_3_C-G) Ni single-atom sites with symmetric and asymmetric configurations on CNTs.

The axial C-substitution site of the NiN_4_ motif on CNTs is denoted as ‘*a*’, whereas the transverse substitution site is denoted as ‘*c’* in the asymmetric NiN_3_C structures.


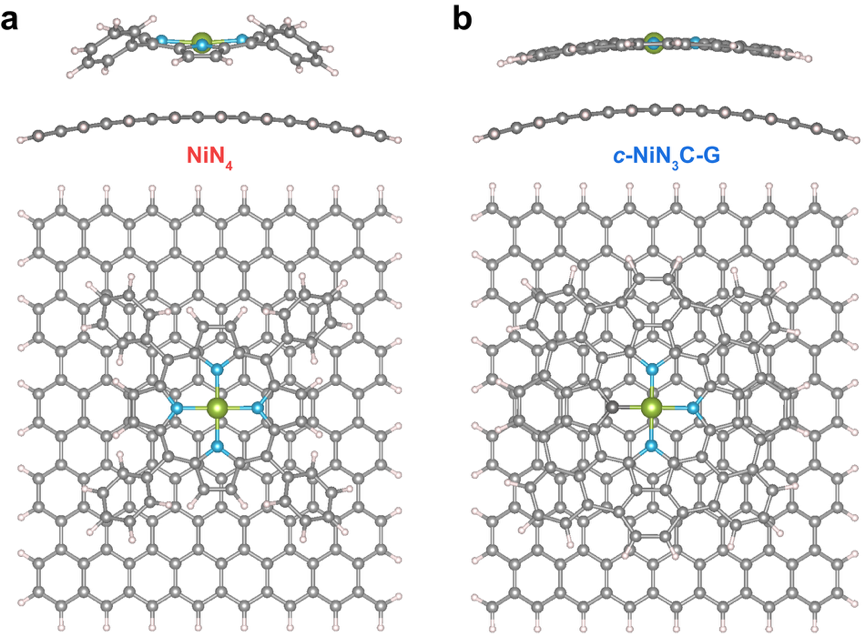


**Figure S3.** DFT-optimized structural models of Ni-based SACs. (a) NiN_4_ and (b) *c*-NiN_3_C-G on a curved graphitic carbon matrix.

The curved graphitic fragment was deliberately constructed to mimic the intrinsic curvature of experimental CNTs (~20 nm in diameter). Top and bottom views correspond to different perspectives of the same optimized structure used for catalyst geometry optimization.


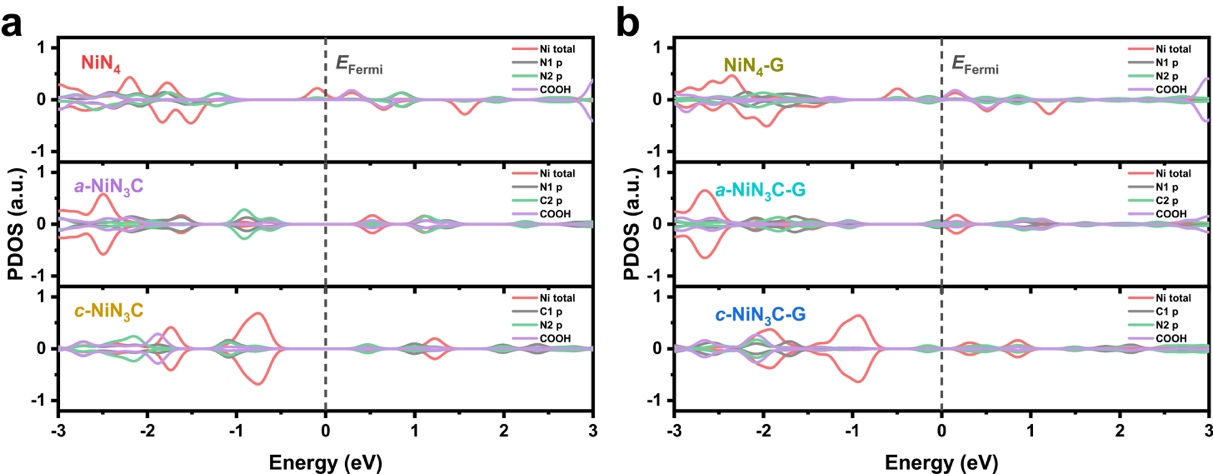


**Figure S4.** PDOS diagrams for Ni *3d*, Ni-N *2p*, Ni-C *2p*, and *COOH-C *2p* orbitals of (a) molecular and (b) graphitic Ni single-atom sites.

PDOS analysis reveals that NiN_4_ and *a*-NiN_3_C retain Ni-centered *COOH adsorption through dominant hybridization between Ni *3d* states and *COOH *p* orbitals, whereas *c*-NiN_3_C suppresses Ni *3d* contributions near the Fermi level (*E*_Fermi_) and instead enhances the interaction between substituted coordinated C *p* and *COOH *p* orbitals, thereby shifting the adsorption site to the adjacent carbon atom. This Ni-to-C anchoring transition is preserved in the graphitic models, confirming that coordination symmetry breaking fundamentally redistributes frontier electronic density and reconfigures the *COOH binding mode.


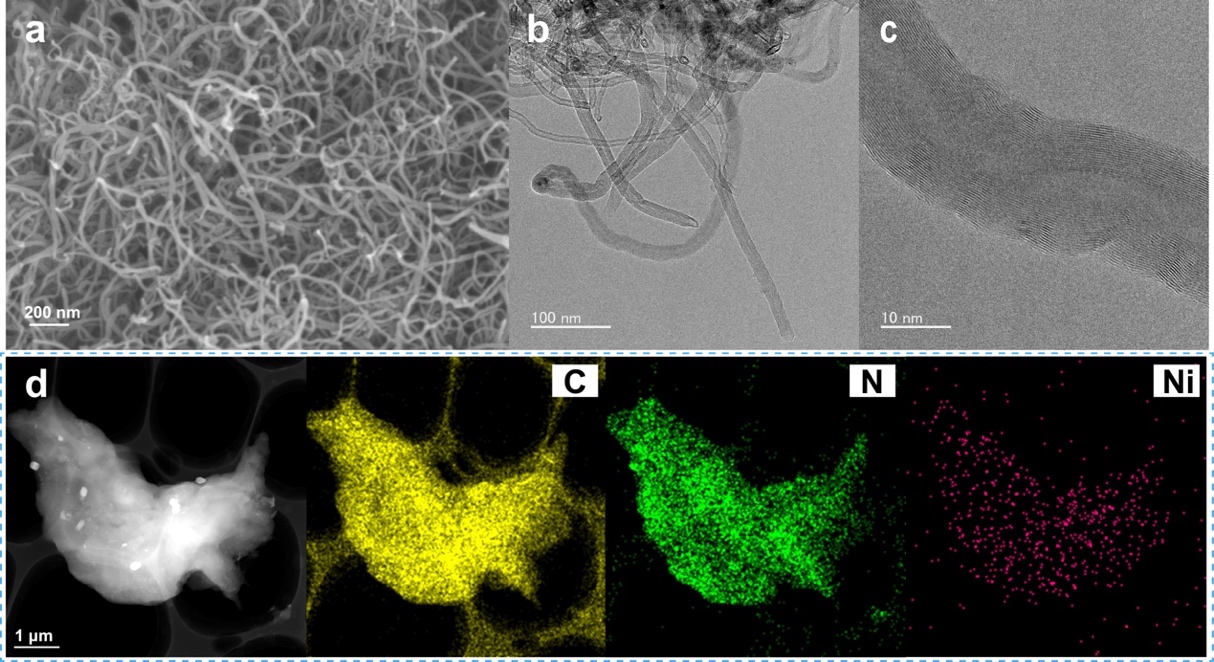


**Figure S5.** Characterization of Ni-CNT. (a) SEM image of Ni-CNT; (b, c) TEM images of Ni-CNT; (d) corresponding TEM-EDS elemental mapping images of Ni-CNT.


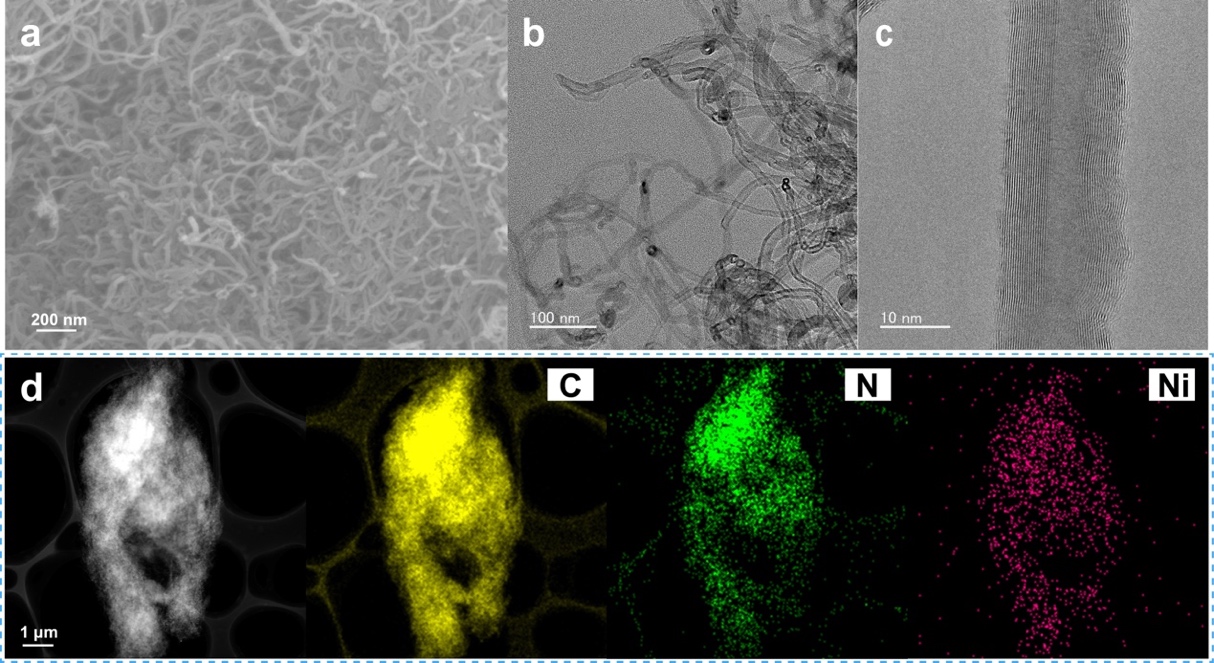


**Figure S6.** Characterization of Ni-CNT-600. (a) SEM image of Ni-CNT-600; (b, c) TEM images of Ni-CNT-600; (d) corresponding TEM-EDS elemental mapping images of Ni-CNT-600.


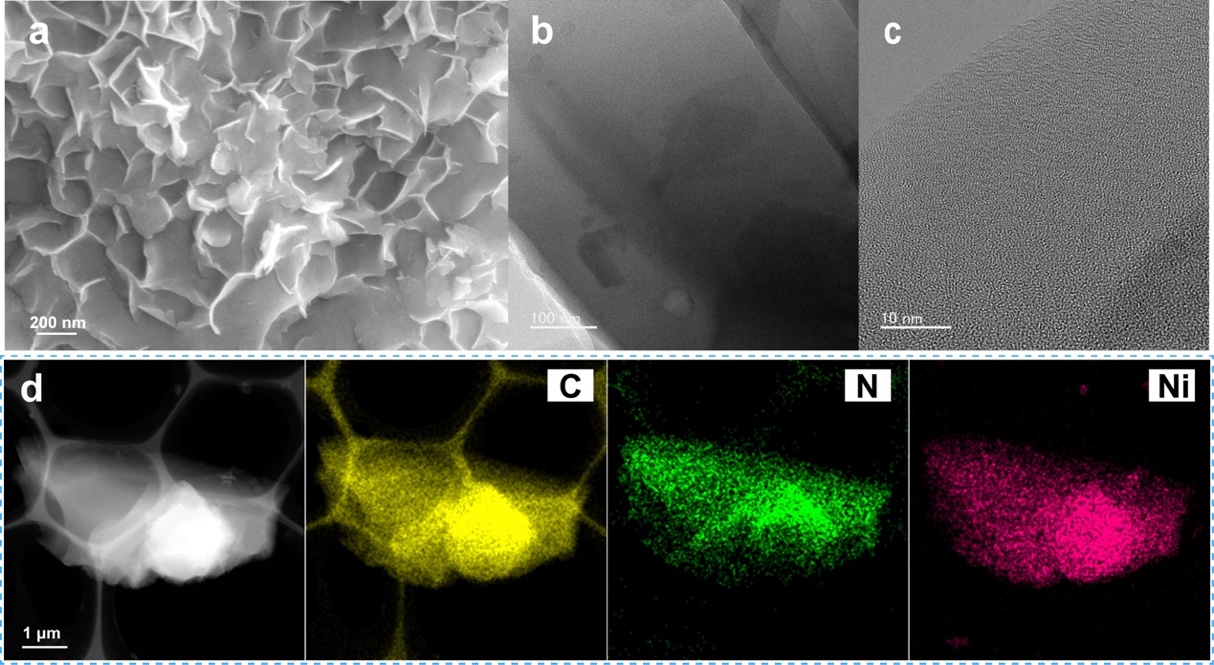


**Figure S7.** Characterization of TPPNi. (a) SEM image of TPPNi; (b, c) TEM images of TPPNi; (d) corresponding TEM-EDS elemental mapping images of TPPNi.


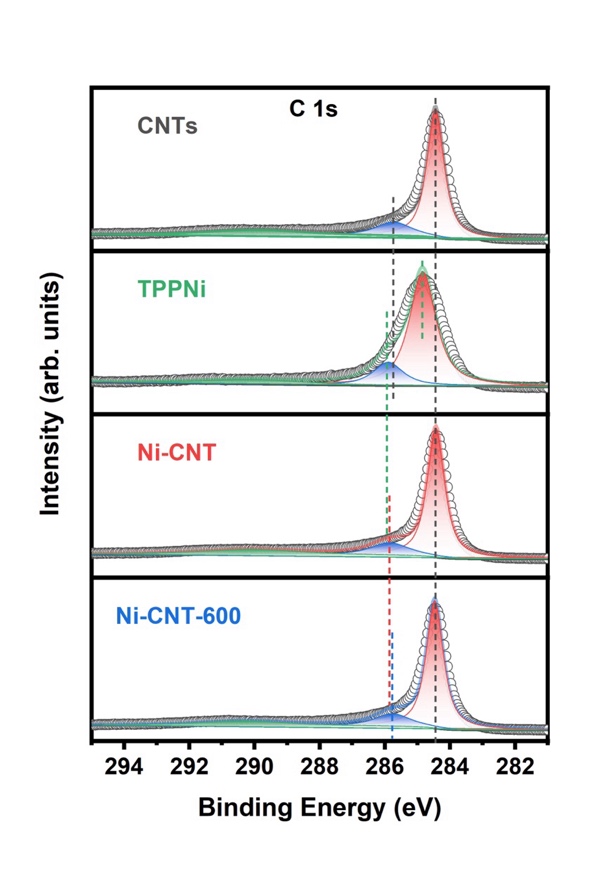


**Figure S8.** C 1s XPS spectra of CNT, TPPNi, Ni-CNT and Ni-CNT-600.

The C 1s spectra illustrate the evolution of carbon chemical environments from pristine CNTs to porphyrin loading and subsequent thermal transformation. CNTs display a dominant *sp^2^* C–C peak at 284.4 eV, accompanied by contributions from C–O (285.7 eV) and O–C=O (289.9 eV), consistent with surface oxygen functionalities. TPPNi shows characteristic peaks at 284.8 eV and 286.0 eV, corresponding to *sp^2^* carbon and C–N bonds within the porphyrin macrocycle. Upon forming Ni-CNT, the main *sp^2^* peak is preserved at 284.4 eV, while a higher-binding-energy feature at 285.9 eV is observed, indicating the coexistence of graphitic carbon and C–N interactions originating from the immobilized porphyrin. After thermal treatment (Ni-CNT-600), the main peak remains at 284.4 eV, but the higher-energy component slightly shifts to 285.8 eV, suggesting the transformation of molecular porphyrin into more stable Ni–N–C coordination (NiN_3_C-like) accompanied by a decrease in oxygenated carbon species. Overall, these results confirm the structural evolution from molecular TPPNi to strongly coupled Ni–N–C active sites on CNTs, which is consistent with the enhanced catalytic activity.


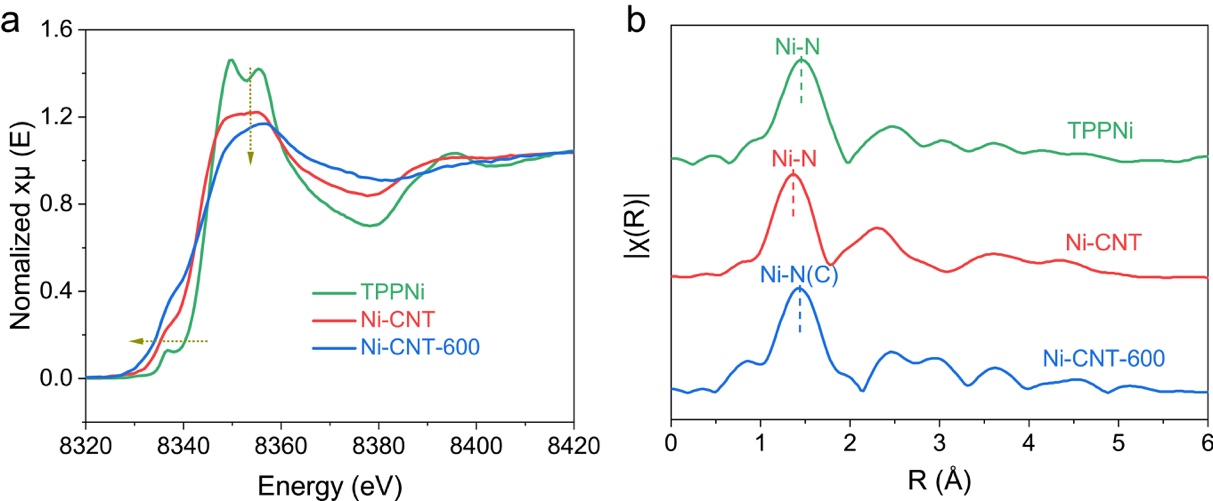


**Figure S9.** (a) Ni K-edge XANES spectra of TPPNi, Ni-CNT and Ni-CNT-600; (b) The plotted Fourier transformation of the EXAFS spectra of TPPNi, Ni-CNT, and Ni-CNT-600.


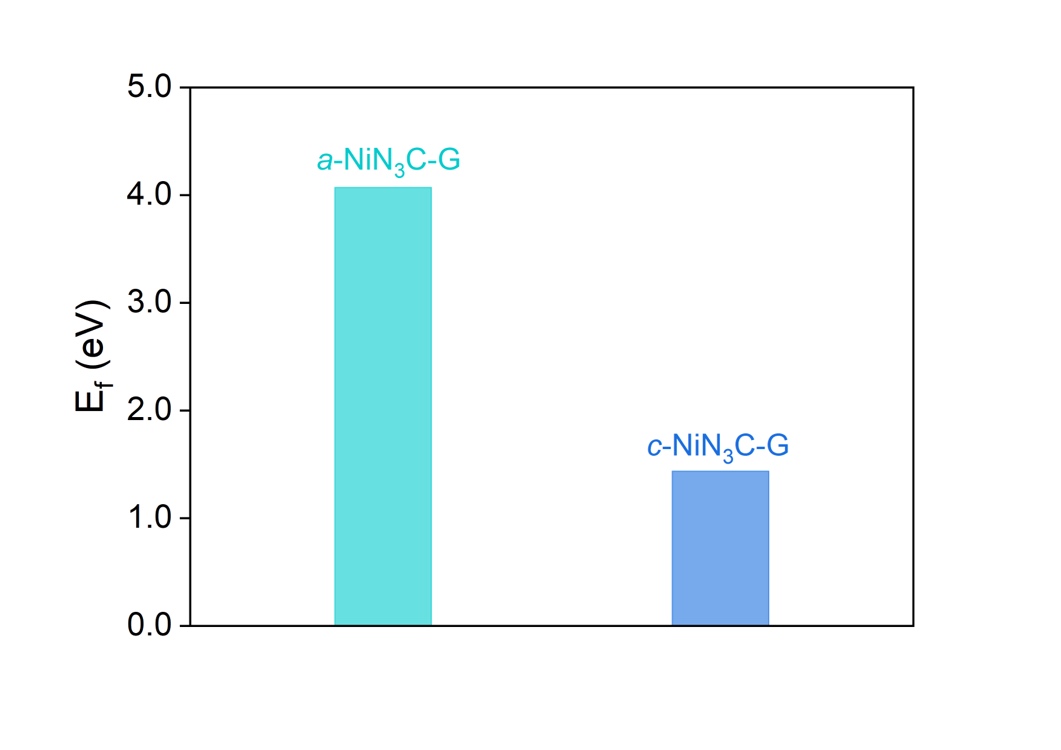


**Figure S10.** C-substitution formation energy of *a*-NiN_3_C-G and *c*-NiN_3_C-G configurations, respectively.

As show in Figure S10, the calculated C-substitution formation energy of *c*-NiN_3_C-G is significantly lower than that of *a*-NiN_3_C-G. This result indicates that the circumferential configuration is energetically more favorable for C substitution in the curved CNT model.


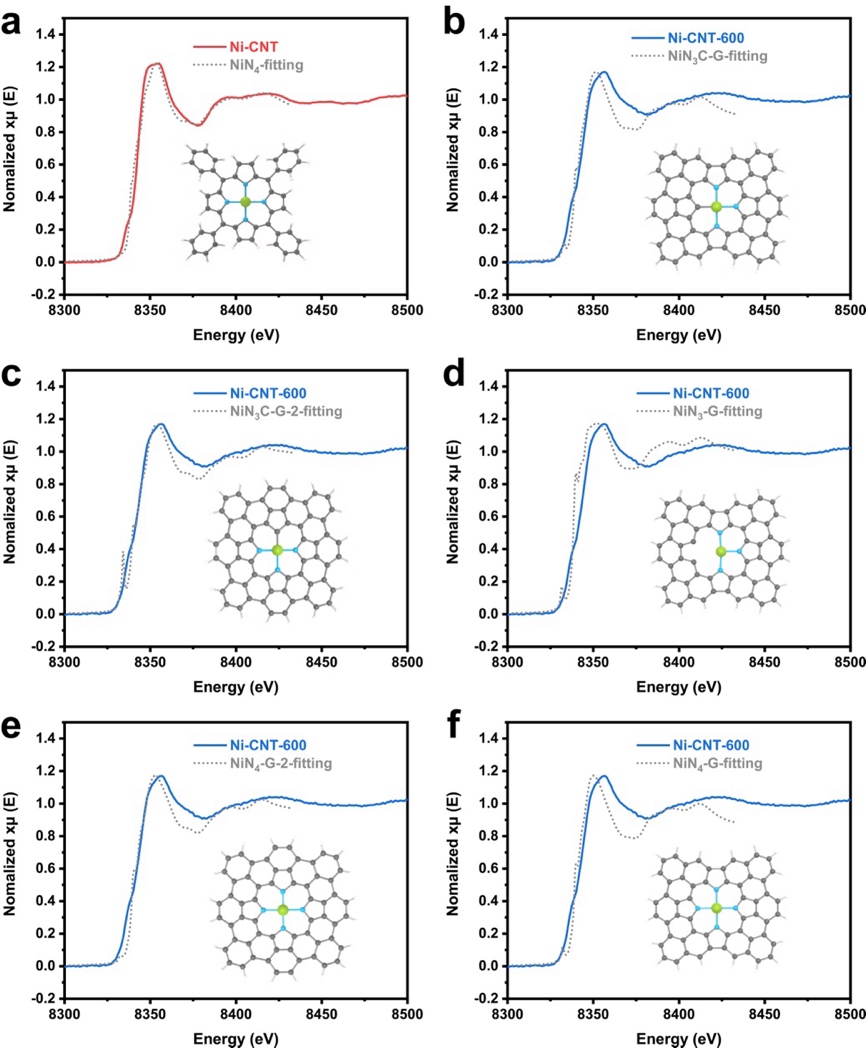


**Figure S11.** Ni K-edge XANES and theoretical fitting of Ni-CNT and Ni-CNT-600 with different Ni-N-C structural models: (a) Ni-CNT XANES spectrum and the corresponding NiN_4_ fitting curves; (b-f) Ni-CNT-600 XANES data and fitting curves based on different Ni–N–C coordination models.

The XANES fitting results demonstrate that Ni-CNT retains a symmetric NiN_4_ coordination environment, consistent with the coordination structure of the porphyrin nickel precursor. After high-temperature treatment, Ni-CNT-600 is better described by an asymmetric NiN_3_C structure rather than NiN_3_ or NiN_4_, confirming a structural transformation from molecular NiN_4_ to defect-coupled NiN_3_C active sites. This coordination transition rationalizes the enhanced electronic coupling and improved catalytic activity observed experimentally.


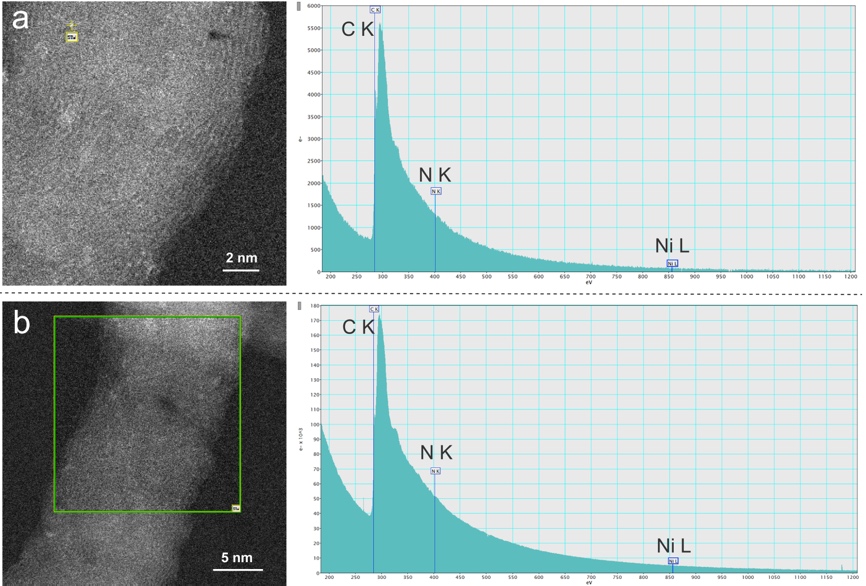


**Figure S12.** Electron energy-loss spectrum (EELS) characterization of Ni-CNT-600. (a) HAADF-STEM image and the corresponding point EELS of Ni-CNT-600 collected from a selected atomic site. (b) HAADF-STEM image of a selected area and the corresponding area EELS obtained from the boxed region on Ni-CNT-600.

Additional EELS measurements were attempted to probe the local coordination environment of Ni-CNT-600. Due to the low Ni loading and weak signal intensity, the EELS spectra collected from selected atomic sites and mapped regions do not allow direct discrimination between axial and circumferential NiN_3_C configurations. Therefore, the structural assignment of *c*-NiN_3_C is mainly based on the combined DFT and XAFS analyses.


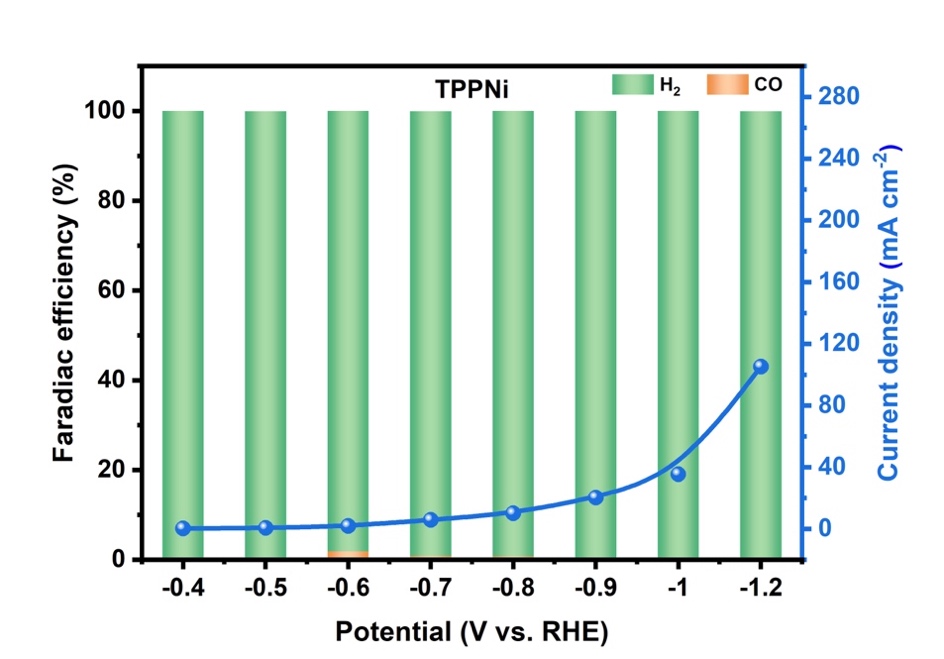


**Figure S13.** Faradaic efficiency and current densities for TPPNi in 1M KOH.


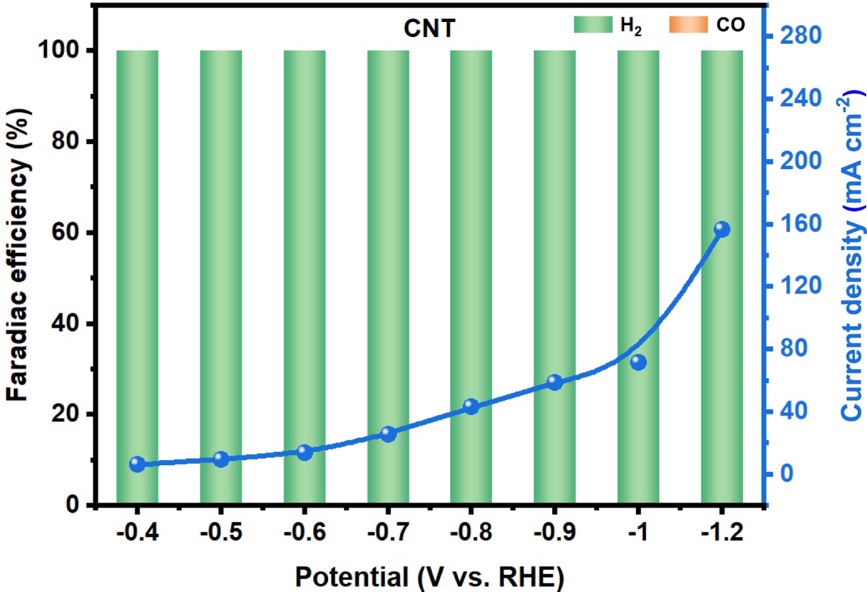


**Figure S14.** Faradaic efficiency and current densities for CNT in 1M KOH.

As shown in Figures S13-S14, TPPNi and CNT exhibit no detectable CO_2_RR activity within the measured potentials range and exclusively catalyze the HER.


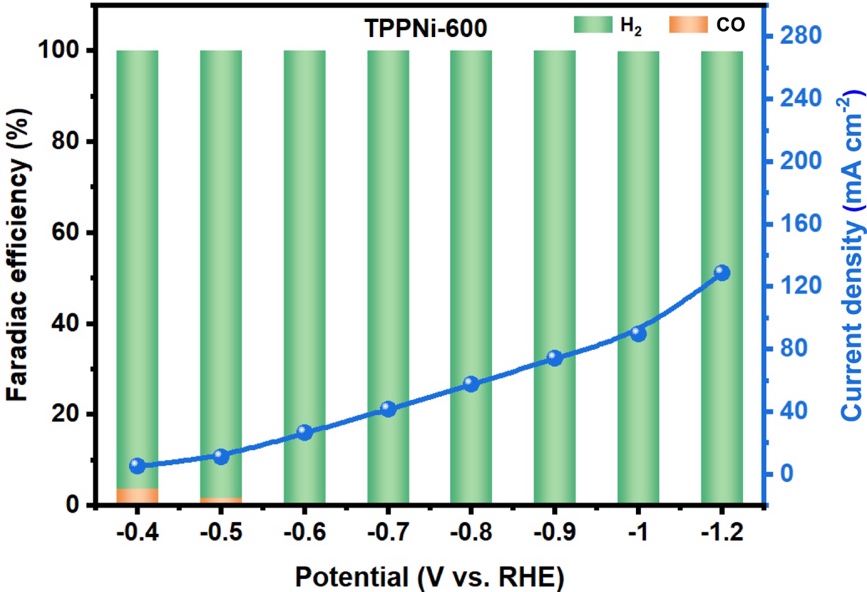


**Figure S15.** Faradaic efficiency and current densities for TPPNi-600 in 1M KOH.


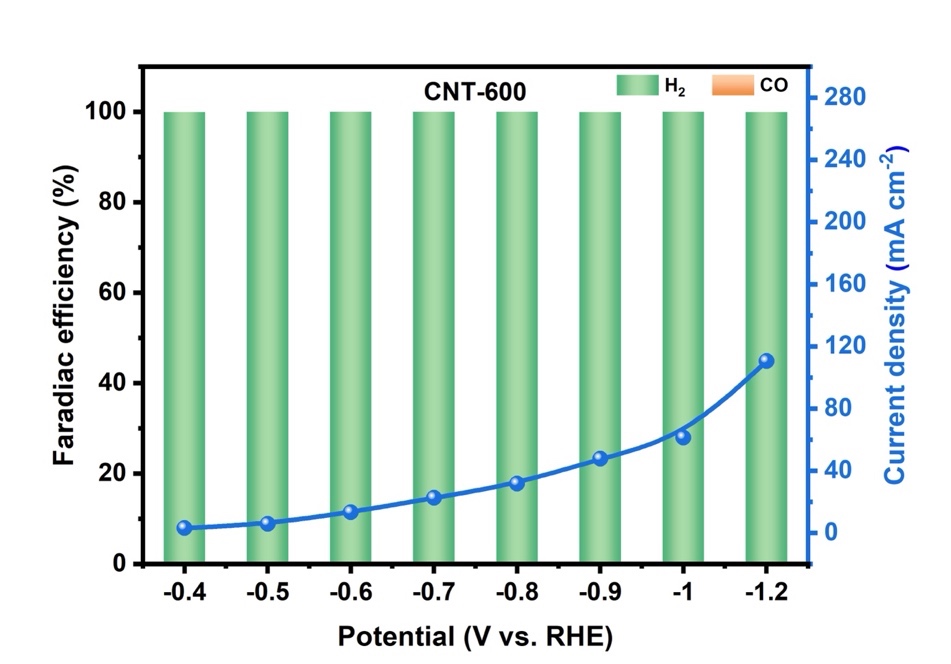


**Figure S16.** Faradaic efficiency and current densities for CNT-600 in 1M KOH.

As shown in Figures S15-S16, both TPPNi-600 and CNT-600 samples exhibit exclusively HER activity, highlighting the pivotal role of the interaction between TPPNi and CNT.


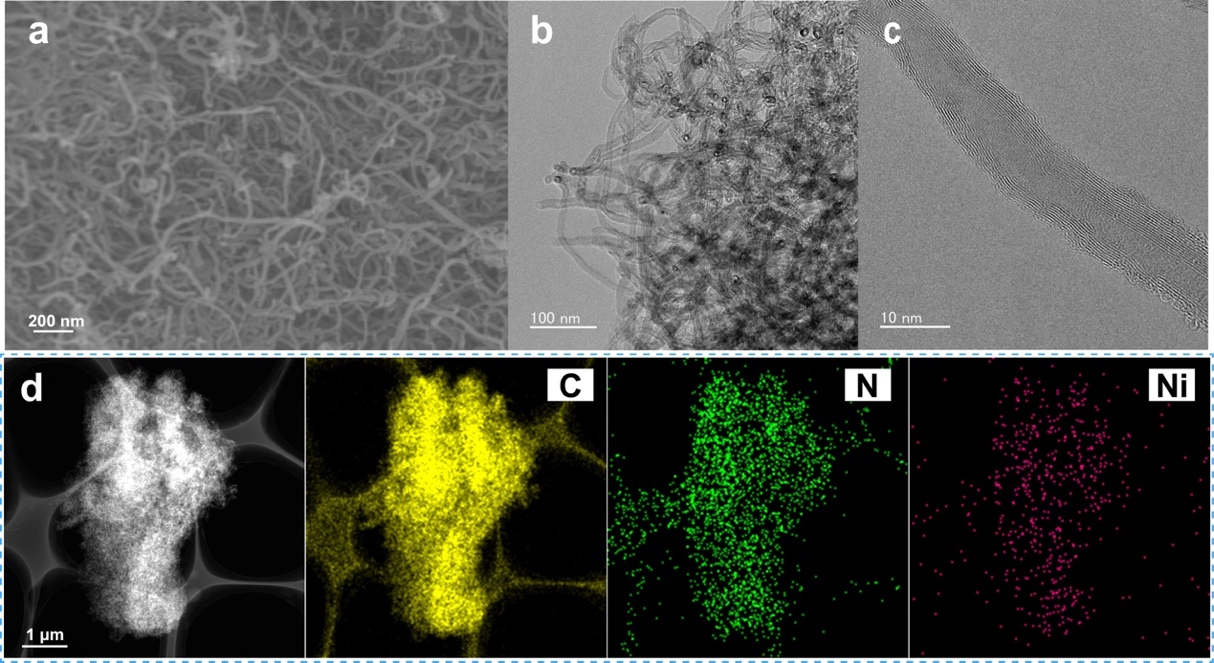


**Figure S17.** Characterization of Ni-CNT-300. (a) SEM image of Ni-CNT-300; (b, c) TEM images of Ni-CNT-300; (d) corresponding TEM-EDS elemental mapping images of Ni-CNT-300.


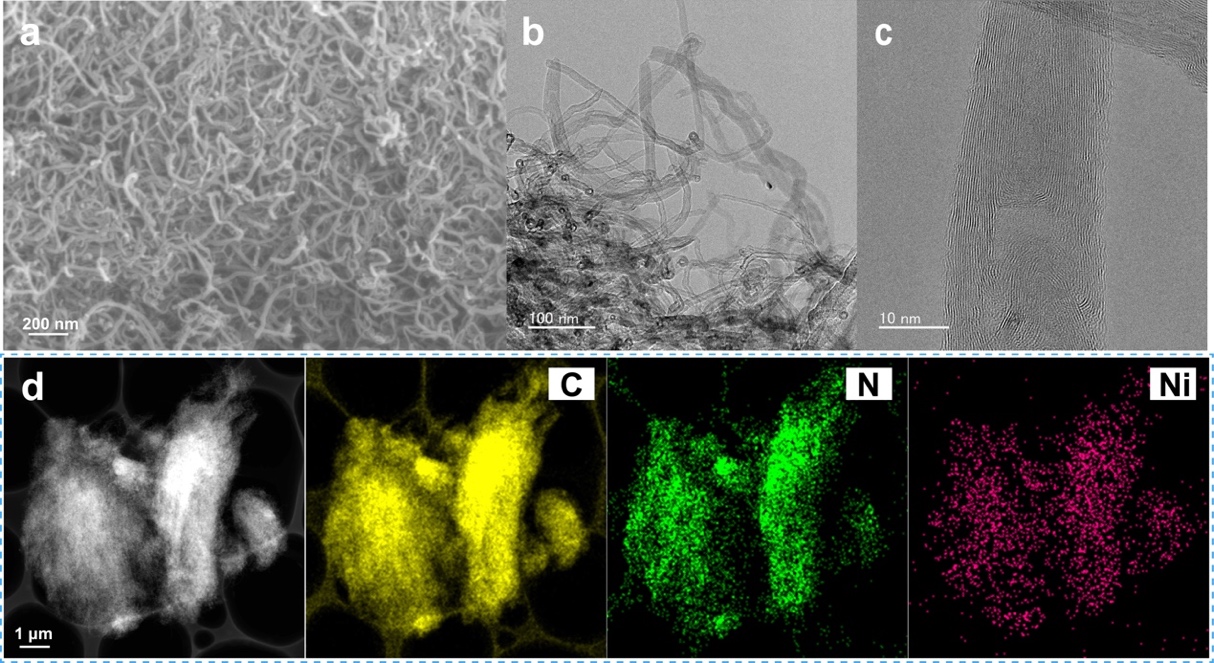


**Figure S18.** Characterization of Ni-CNT-450. (a) SEM image of Ni-CNT-450; (b, c) TEM images of Ni-CNT-450; (d) corresponding TEM-EDS elemental mapping images of Ni-CNT-450.


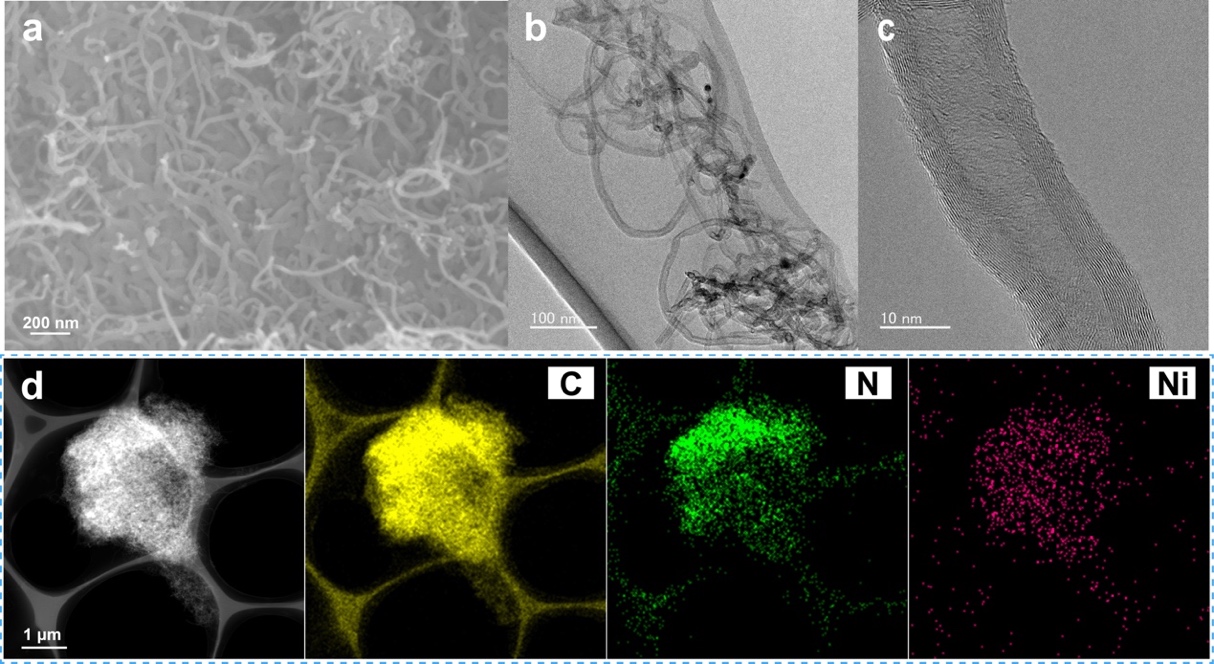


**Figure S19.** Characterization of Ni-CNT-750. (a) SEM image of Ni-CNT-750; (b, c) TEM images of Ni-CNT-750; (d) corresponding TEM-EDS elemental mapping images of Ni-CNT-750.


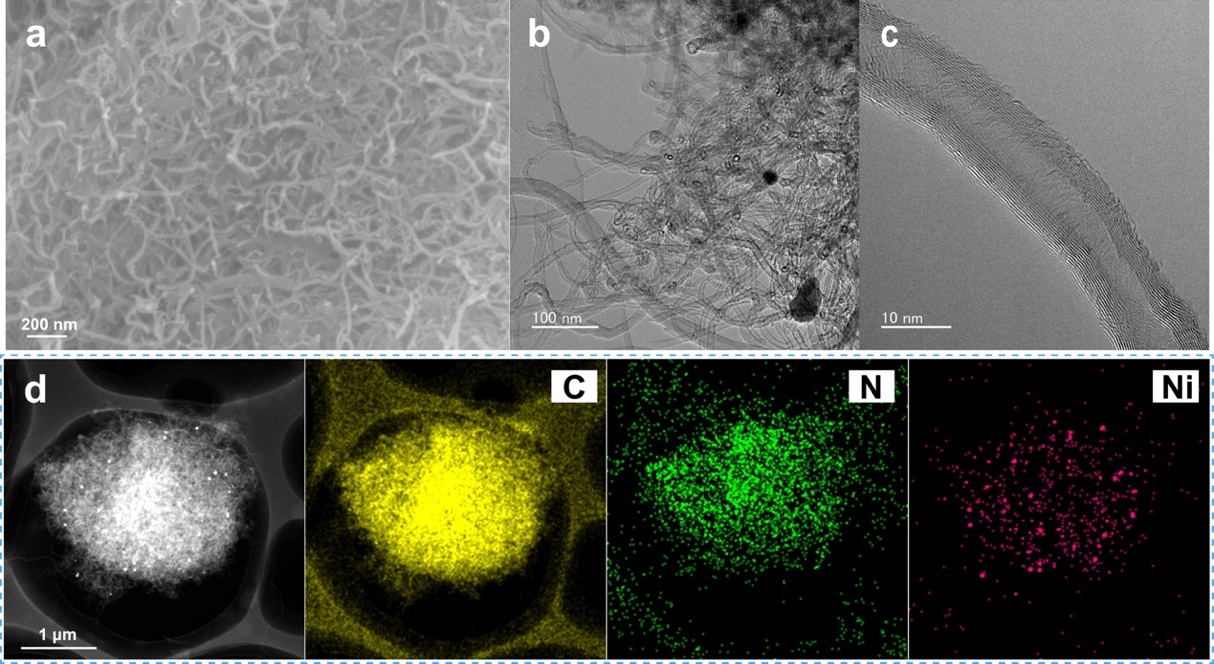


**Figure S20.** Characterization of Ni-CNT-900. (a) SEM image of Ni-CNT-900; (b, c) TEM images of Ni-CNT-900; (d) corresponding TEM-EDS elemental mapping images of Ni-CNT-900.


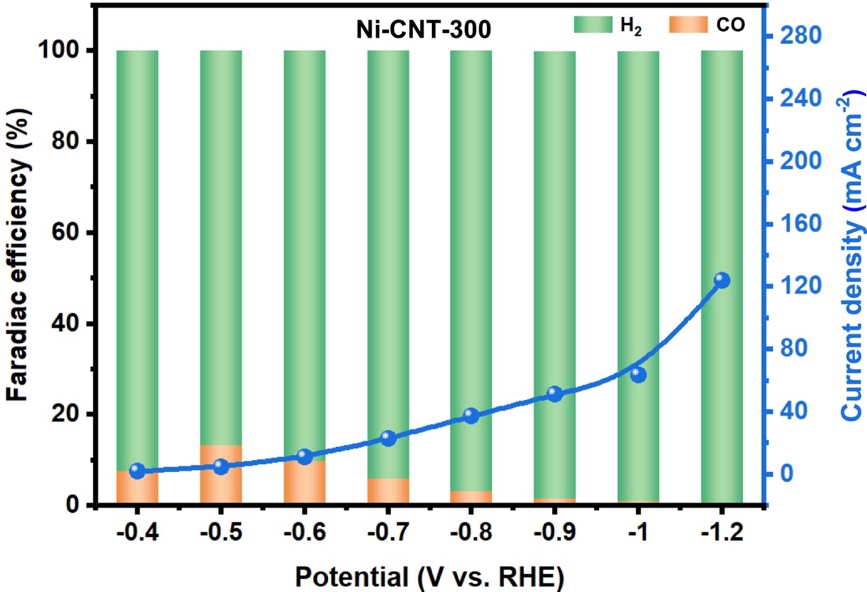


**Figure S21.** Faradaic efficiency and current densities for Ni-CNT-300 in 1M KOH.

Ni-CNT-300 predominantly drives the HER across the tested potential range, with only minor CO_2_RR activity. The maximum FE of CO reaches 13.2% at -0.5 V vs RHE, indicating poor CO_2_RR selectivity relative to HER.


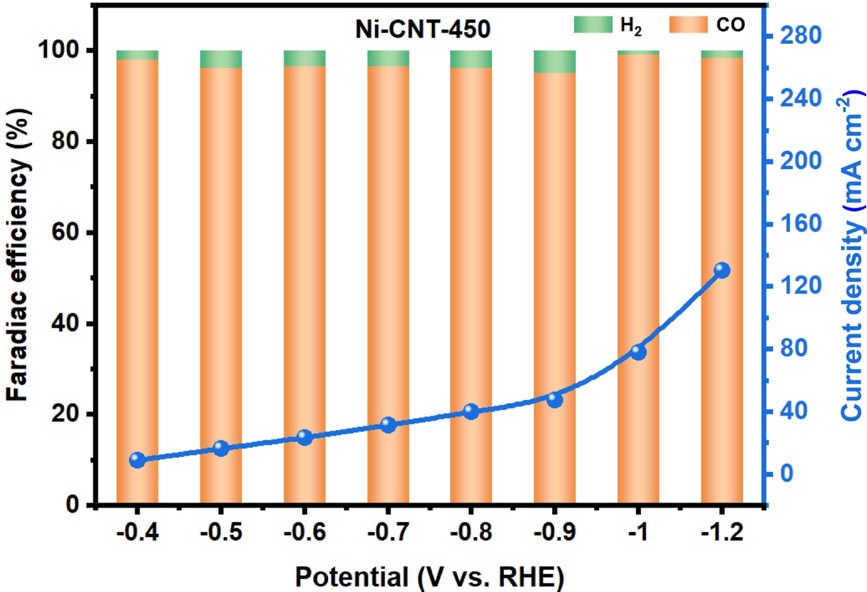


**Figure S22.** Faradaic efficiency and current densities for Ni-CNT-450 in 1M KOH.

Ni-CNT-450 shows near-exclusive CO selectivity (FE_CO_ > 95%, reaching ~99% FE at -1.0 V vs. RHE) but a limited *J*_CO_ of 128 mA cm^-2^, indicating high selectivity with moderate activity, likely due to partial formation of asymmetric Ni–N–C sites and/or residual organics.


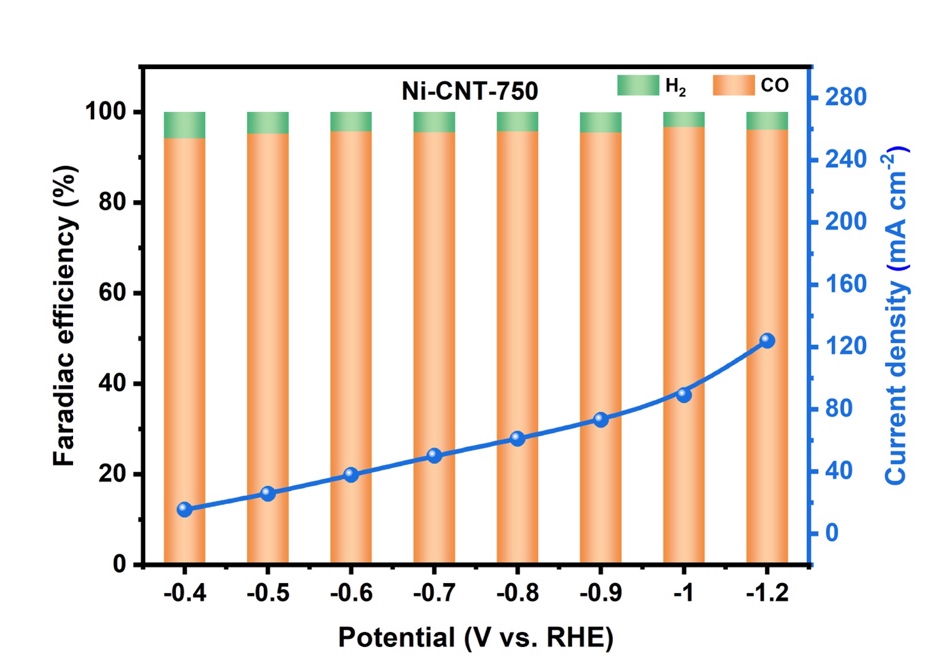


**Figure S23.** Faradaic efficiency and current densities for Ni-CNT-750 in 1M KOH.

Ni-CNT-750 maintains high CO selectivity (FE_CO_ > 94%) but exhibits only 119 mA cm^-2^ of *J*_CO_ at -1.2 V vs. RHE, which may be attributed to partial collapse of Ni–N–C structure associated with the formation of Ni(I) species.


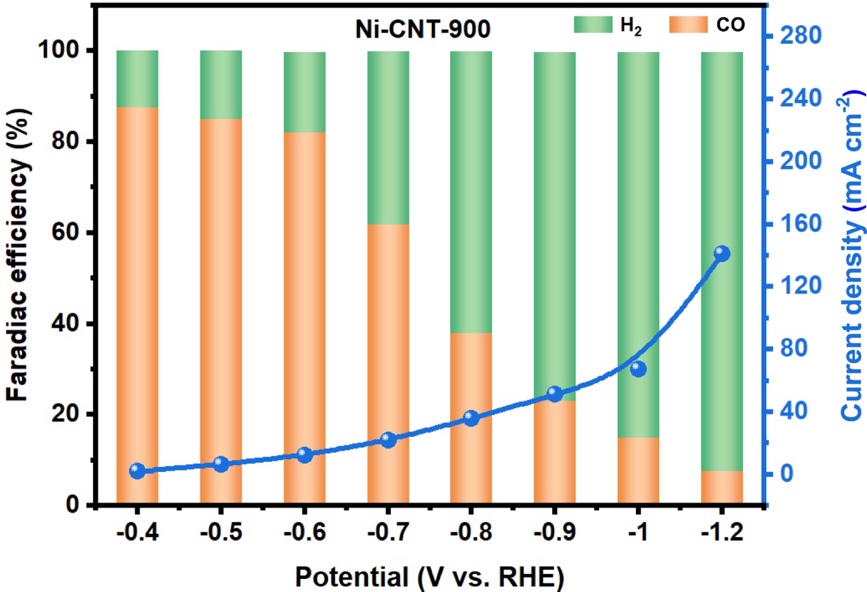


**Figure S24.** Faradaic efficiency and current densities for Ni-CNT-900 in 1M KOH.

Ni-CNT-900 shows severely suppressed CO_2_RR activity (FE_CO_ dropping below 10% at -1.2 V vs. RHE), consistent with EPR-indicated Ni–N–C structural collapse and HER dominance.


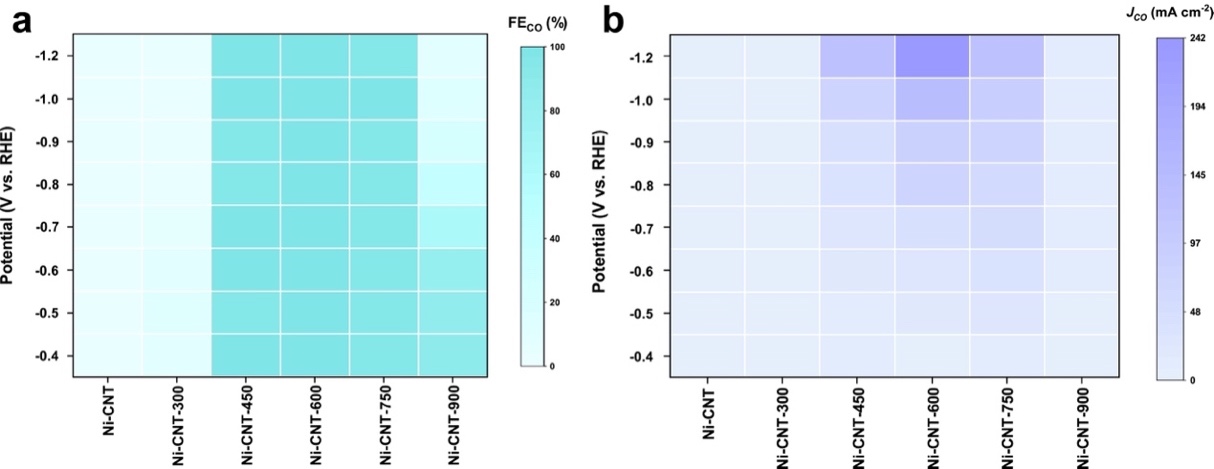


**Figure S25.** Comparison of FE_CO_ (a) and *J*_CO_ (b) for Ni-based catalysts treated at different temperatures under various applied potentials.

As shown in Figure S25, Ni-CNT-450, Ni-CNT-600, and Ni-CNT-750 all exhibit high CO Faradaic efficiencies (FE_CO_ > 94 %), whereas Ni-CNT-600 delivers the highest *J*_CO_, highlighting its superior intrinsic activity. On this basis, Ni-CNT and Ni-CNT-600 were selected as representative catalysts to probe the intrinsic origins of HER- versus CO_2_RR-dominiated reaction pathways.


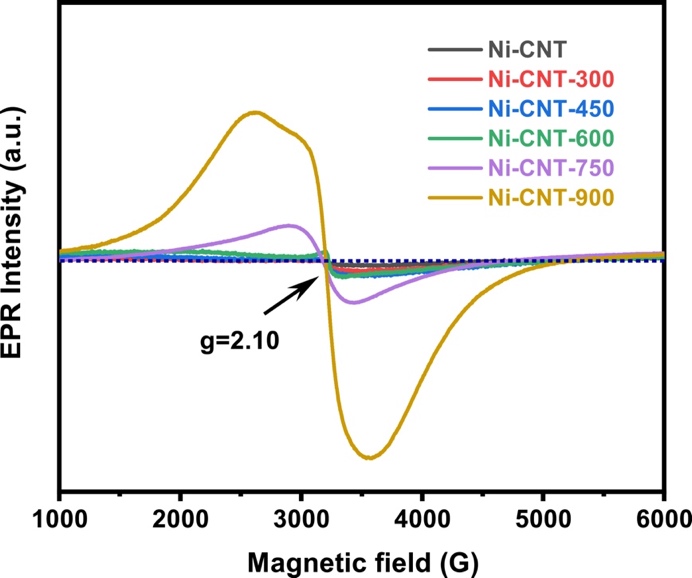


**Figure S26.** Electron spin resonance (ESR) spectra of Ni-CNT and Ni-CNT-based catalysts treated at different calcinated temperatures, measured at room temperature.

Weak but slightly enhanced ESR signals are observed for Ni-CNT-based samples calcined below 600 ^o^C (Ni-CNT, Ni-CNT-300, Ni-CNT-450 and Ni-CNT-600), indicative of partial reduction of Ni(II) species while the Ni–N–C coordination environment remains largely preserved. For higher-temperature-treated samples (Ni-CNT-750 and Ni-CNT-900), pronounced ESR signals characteristic of Ni(I) species emerge, with evident peak splitting (g ≈ ~2.38) observed for Ni-CNT-900, signifying processive disruption of the Ni–N–C coordination environment and further reduction of the Ni center. The obtained *g*-value of ~2.10 can be assigned to an unpaired electron predominantly residing in the *3d*_x2-y2_ orbital, consistent with a Ni(I)-related paramagnetic state.


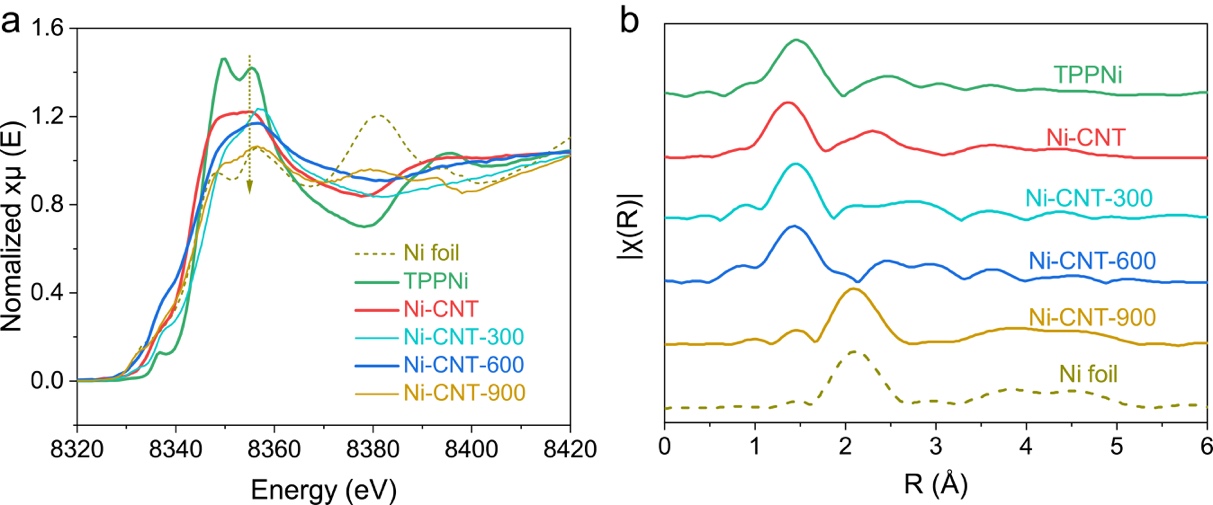


**Figure S27.** (a) Ni K-edge XANES spectra and (b) The plotted Fourier transformation of the EXAFS spectra of Ni foil, TPPNi, Ni-CNT, Ni-CNT-300, Ni-CNT-600 and Ni-CNT-900.


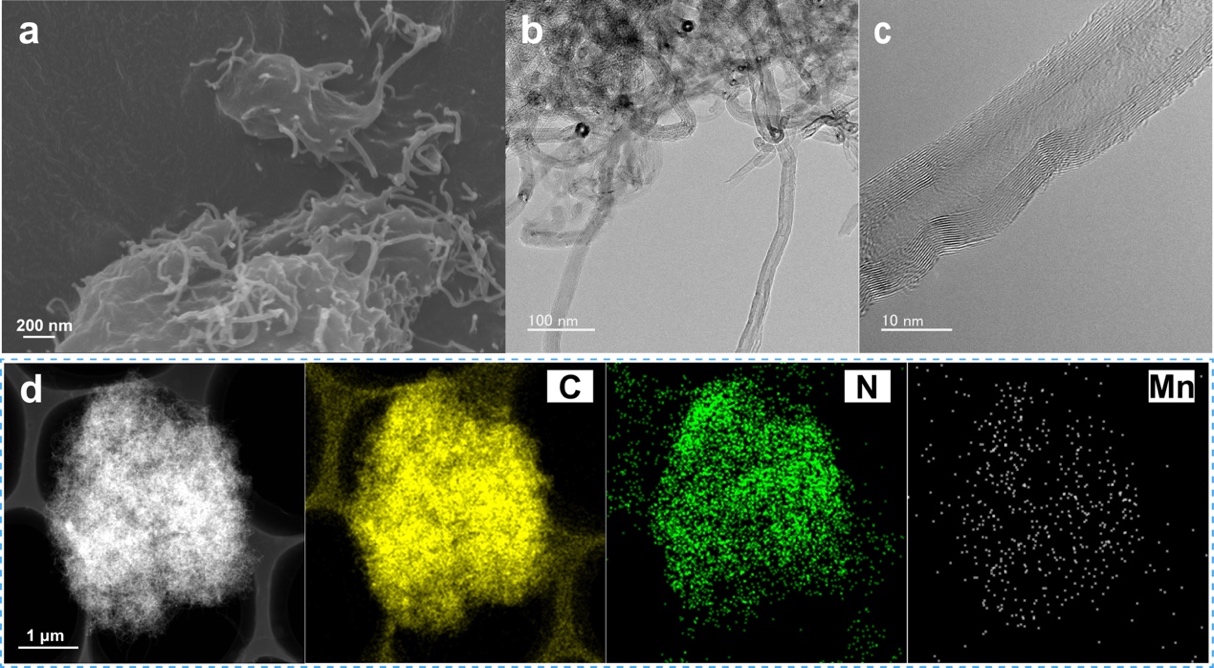


**Figure S28.** Characterization of Mn-CNT. (a) SEM image of Mn-CNT; (b, c) TEM images of Mn-CNT; (d) corresponding TEM-EDS elemental mapping images of Mn-CNT.


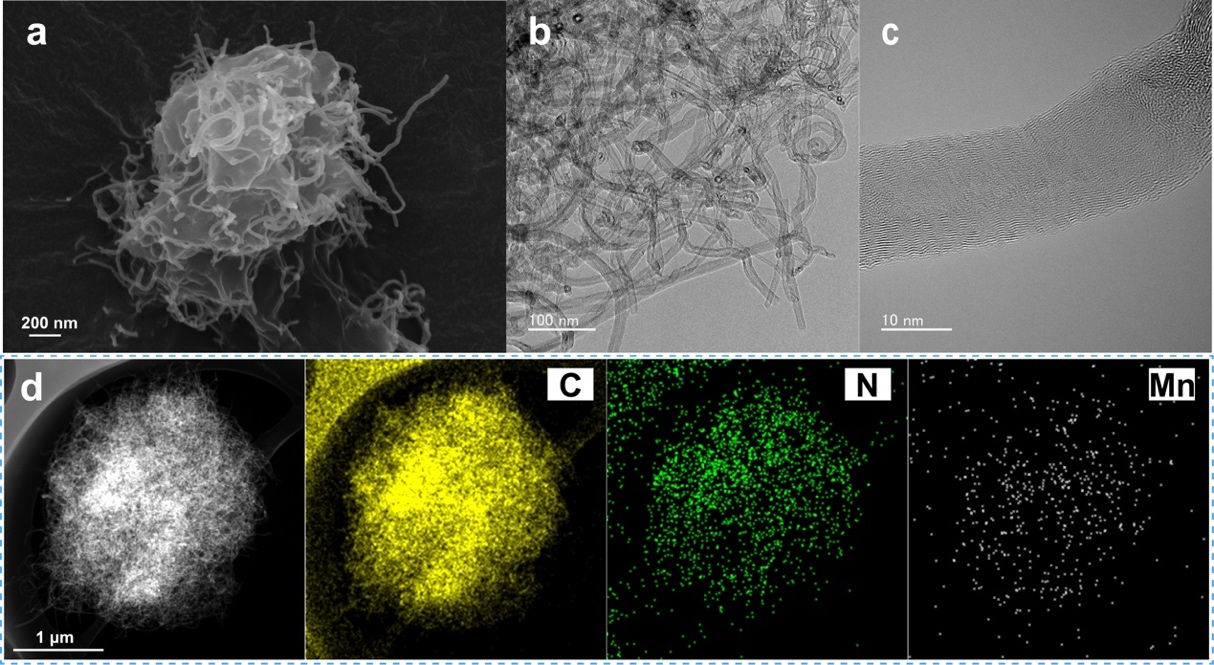


**Figure S29.** Characterization of Mn-CNT-600. (a) SEM image of Mn-CNT-600; (b, c) TEM images of Mn-CNT-600; (d) corresponding TEM-EDS elemental mapping images of Mn-CNT-600.


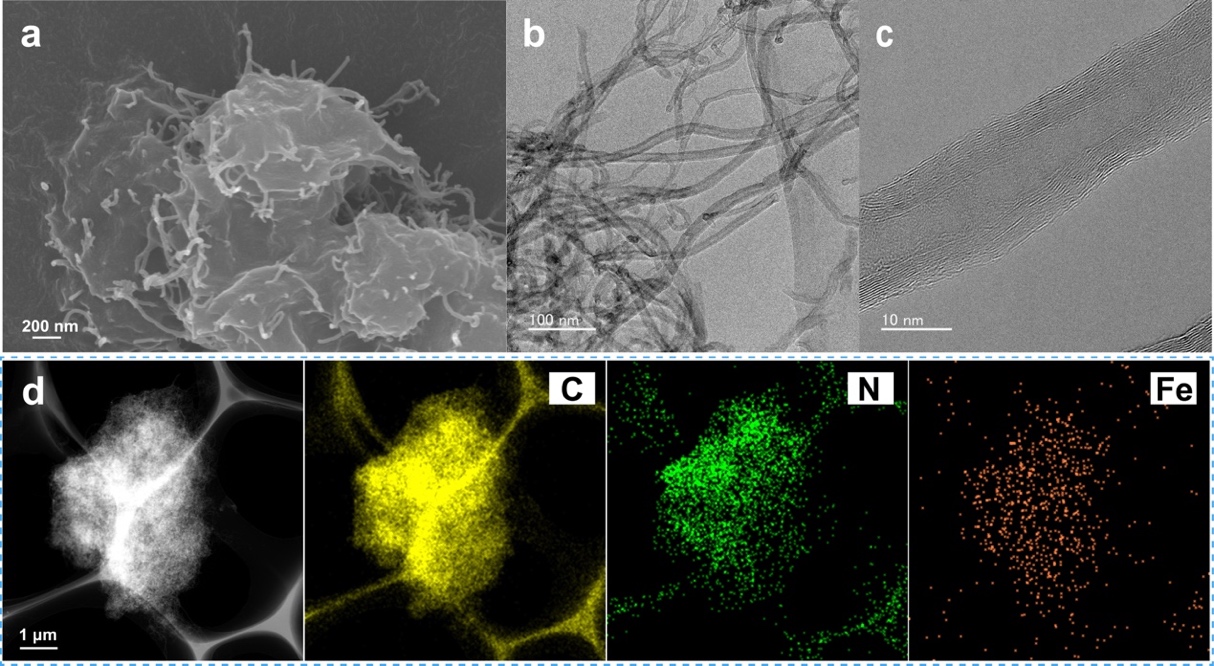


**Figure S30.** Characterization of Fe-CNT. (a) SEM image of Fe-CNT; (b, c) TEM images of Fe-CNT; (d) corresponding TEM-EDS elemental mapping images of Fe-CNT.


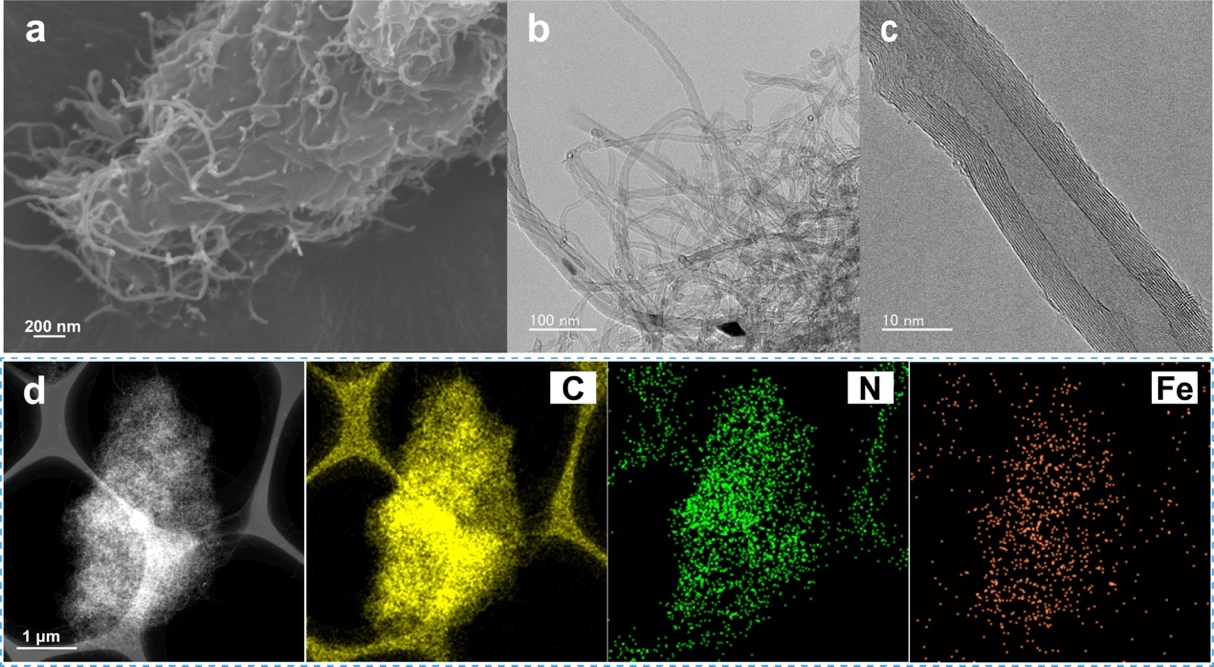


**Figure S31.** Characterization of Fe-CNT-600. (a) SEM image of Fe-CNT-600; (b, c) TEM images of Fe-CNT-600; (d) corresponding TEM-EDS elemental mapping images of Fe-CNT-600.


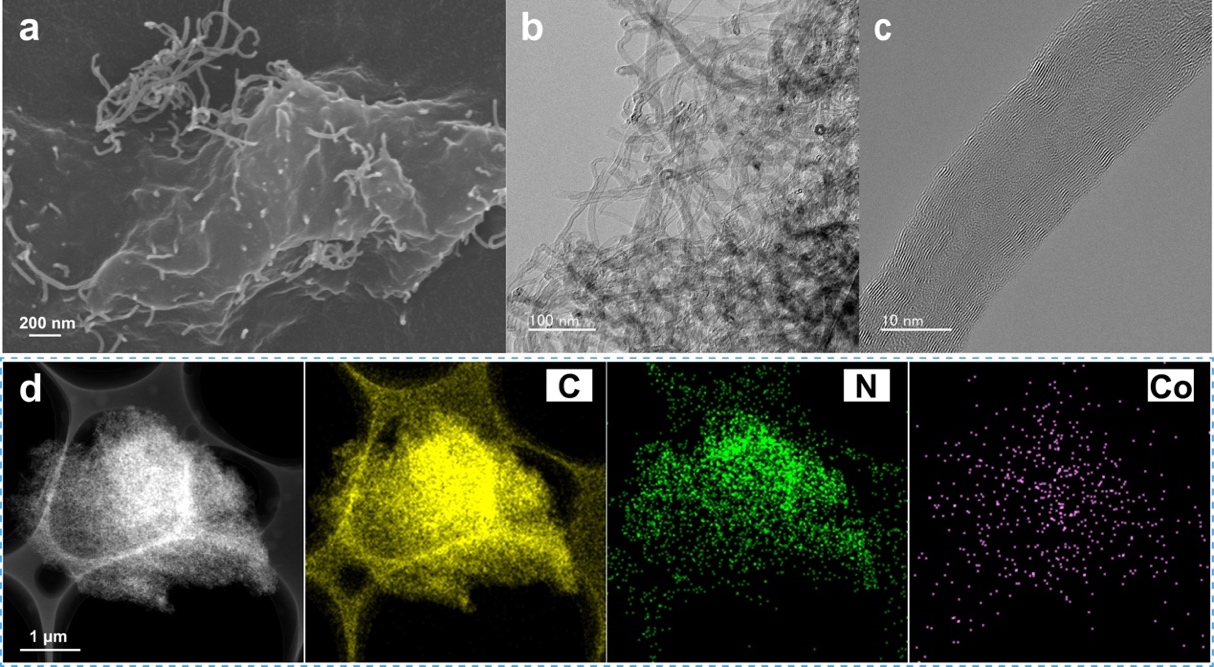


**Figure S32.** Characterization of Co-CNT. (a) SEM image of Co-CNT; (b, c) TEM images of Co-CNT; (d) corresponding TEM-EDS elemental mapping images of Co-CNT.


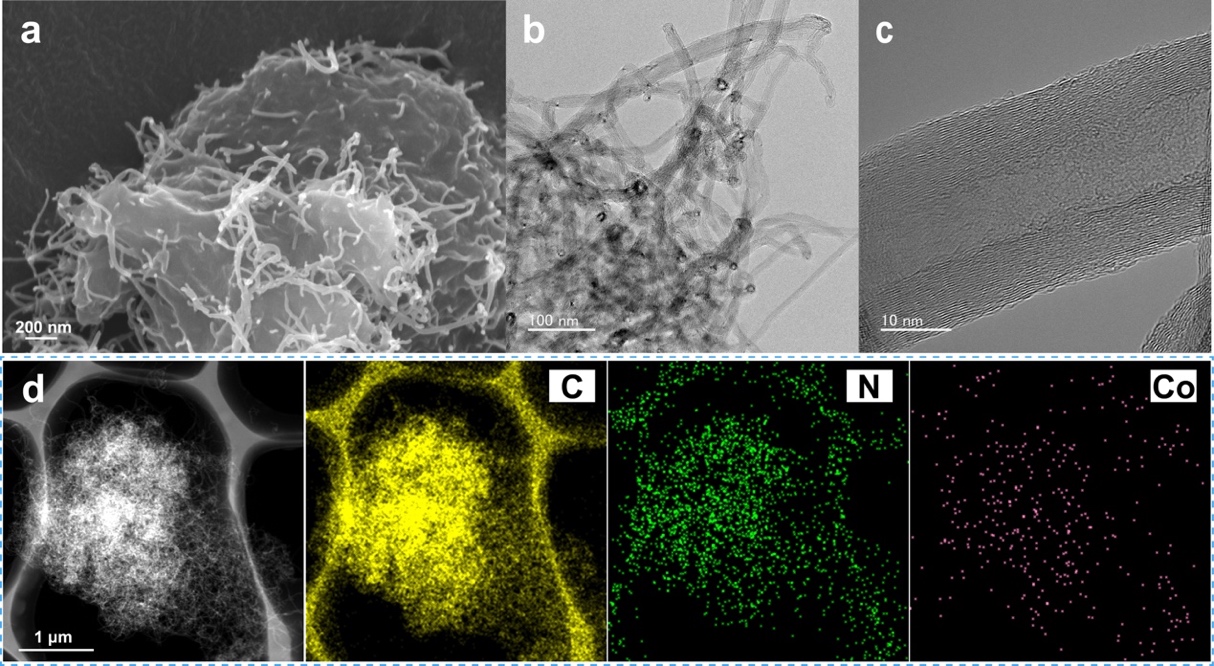


**Figure S33.** Characterization of Co-CNT-600. (a) SEM image of Co-CNT-600; (b, c) TEM images of Co-CNT-600; (d) corresponding TEM-EDS elemental mapping images of Co-CNT-600.


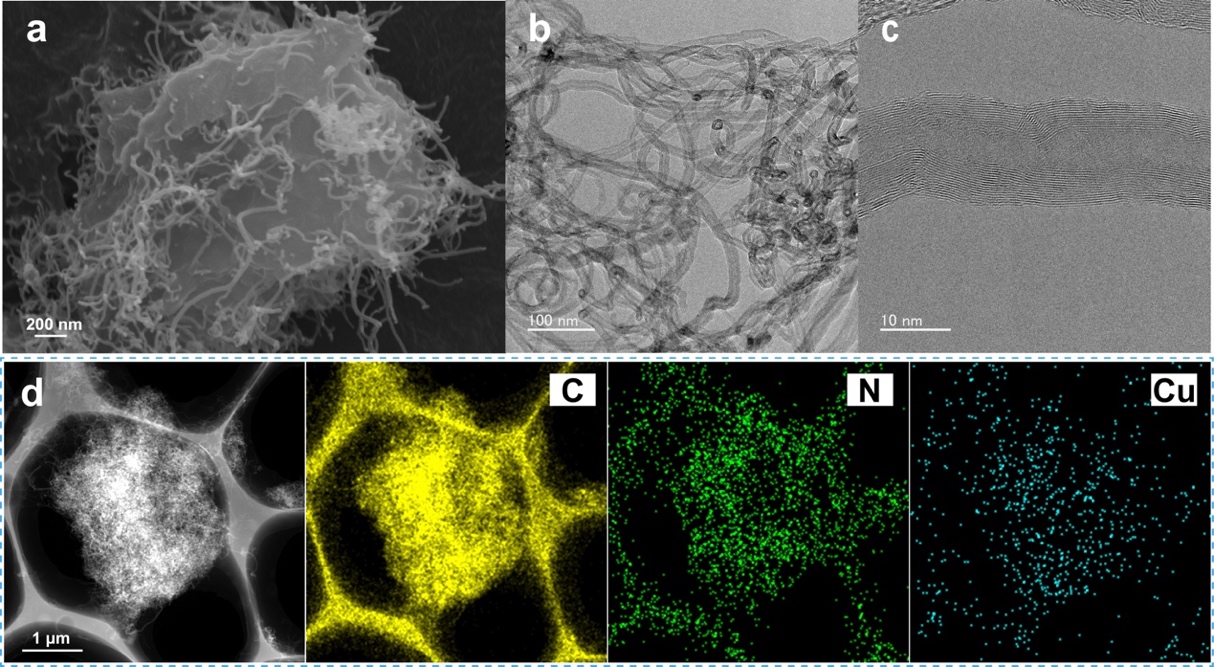


**Figure S34.** Characterizations for Cu-CNT. (a) SEM image of Cu-CNT; (b, c) TEM images of Cu-CNT; (d) corresponding TEM-EDS elemental mapping images of Cu-CNT.


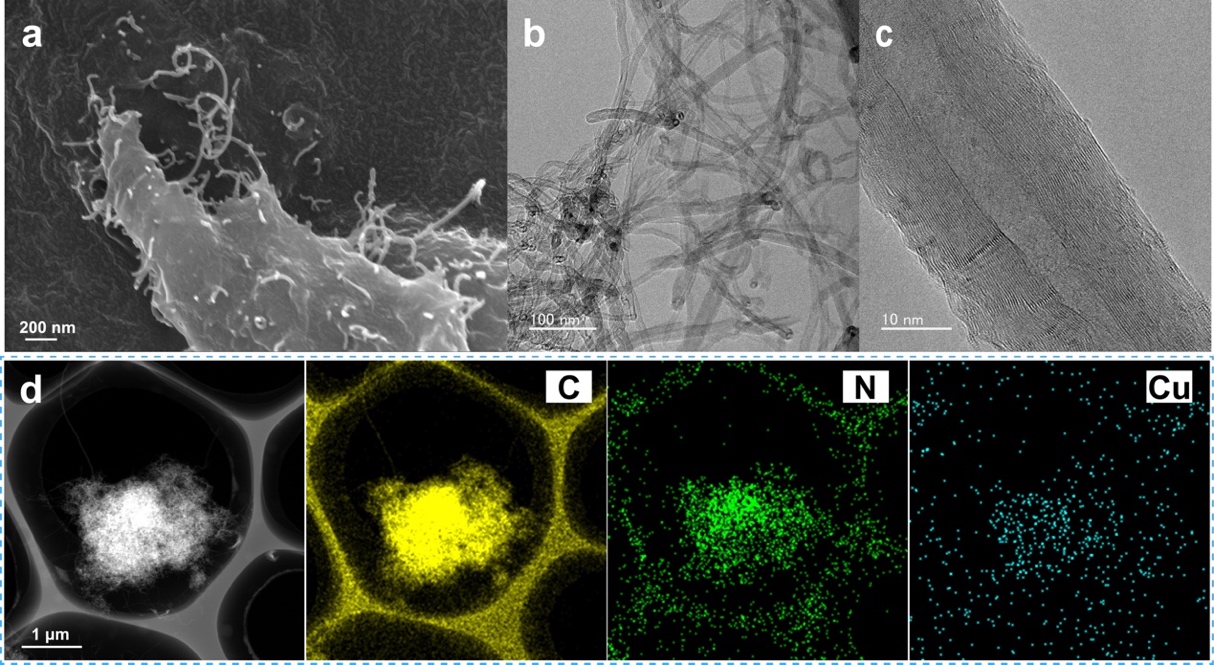


**Figure S35.** Characterization of Cu-CNT-600. (a) SEM image of Cu-CNT-600; (b, c) TEM images of Cu-CNT-600; (d) corresponding TEM-EDS elemental mapping images of Cu-CNT-600.


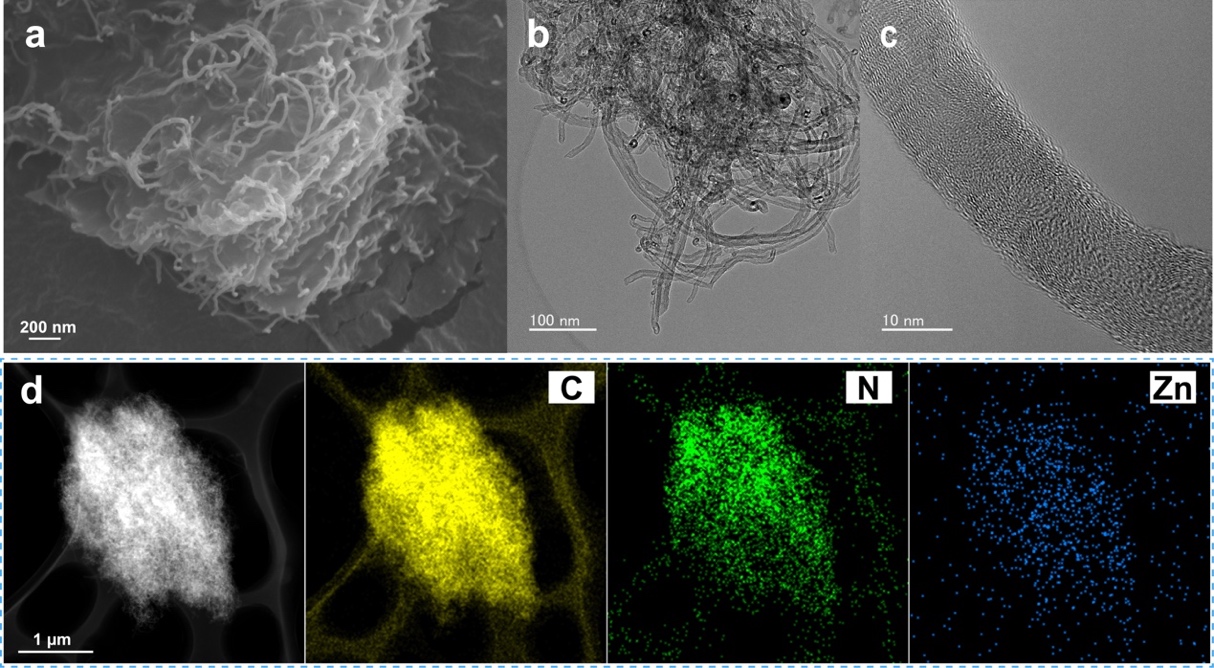


**Figure S36.** Characterization of Zn-CNT. (a) SEM image of Zn-CNT; (b, c) TEM images of Zn-CNT; (d) corresponding TEM-EDS elemental mapping images of Zn-CNT.


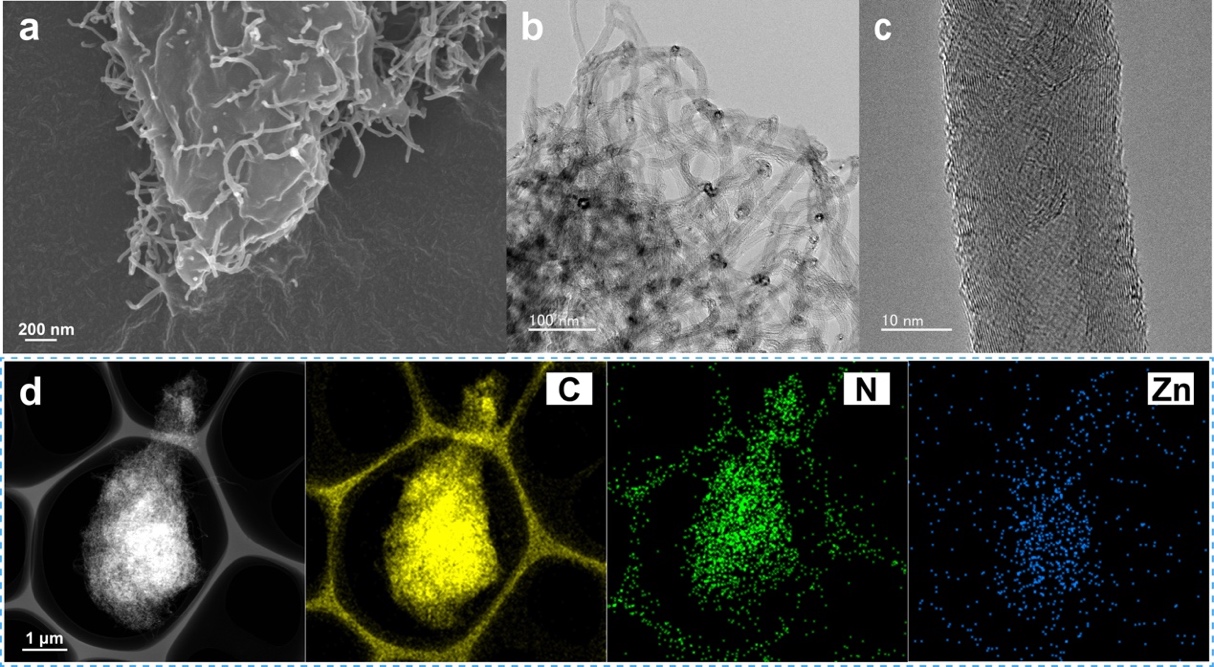


**Figure S37.** Characterization of Zn-CNT-600. (a) SEM image of Zn-CNT-600; (b) and (c) TEM images of Zn-CNT-600; (d) corresponding TEM-EDS elemental mapping images of Zn-CNT-600.


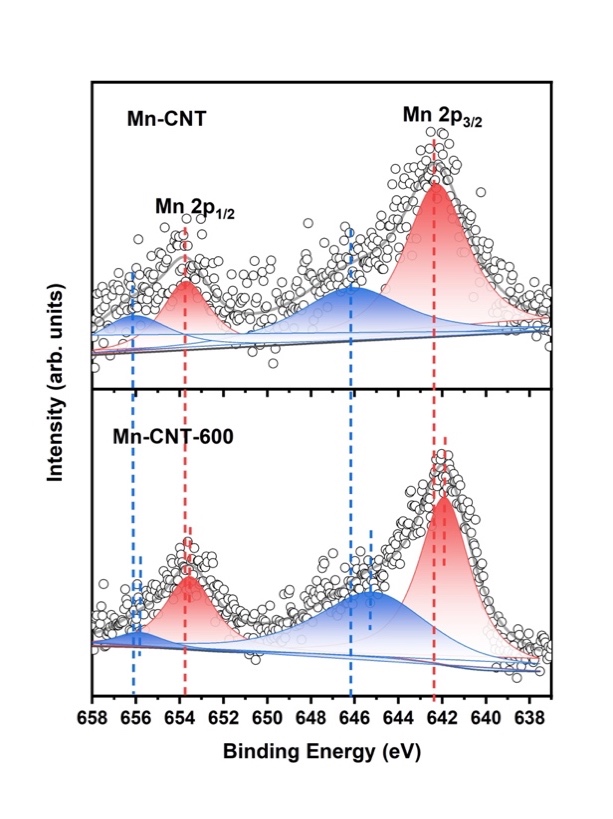


**Figure S38.** Mn 2p XPS spectra of Mn-CNT and Mn-CNT-600.


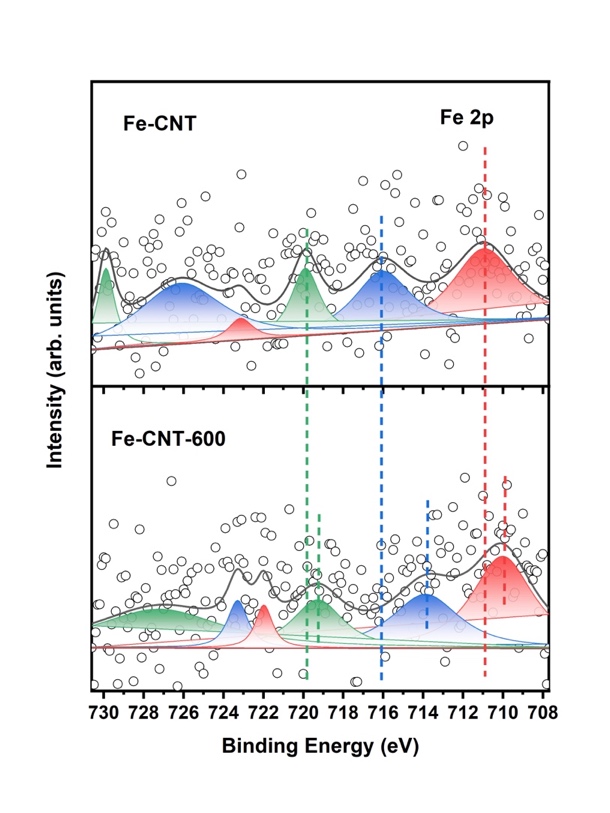


**Figure S39.** Fe 2p XPS spectra of Fe-CNT and Fe-CNT-600.


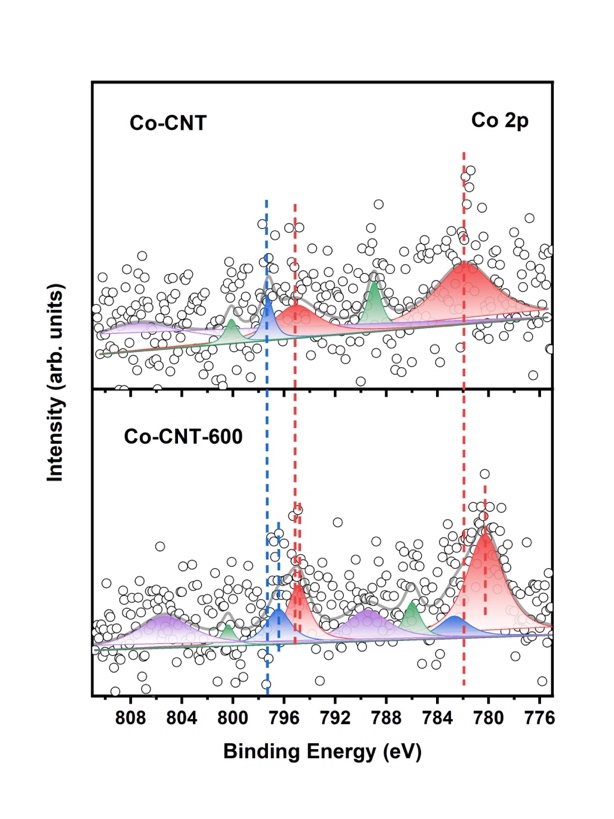


**Figure S40.** Co 2p XPS spectra of Co-CNT and Co-CNT-600.

**
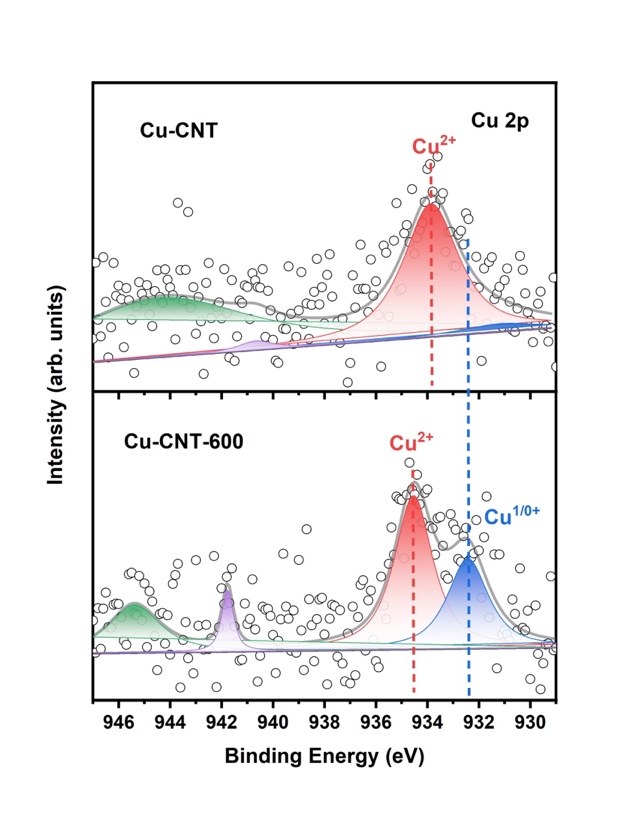
**

**Figure S41.** Cu 2p XPS spectra of Cu-CNT and Cu-CNT-600.


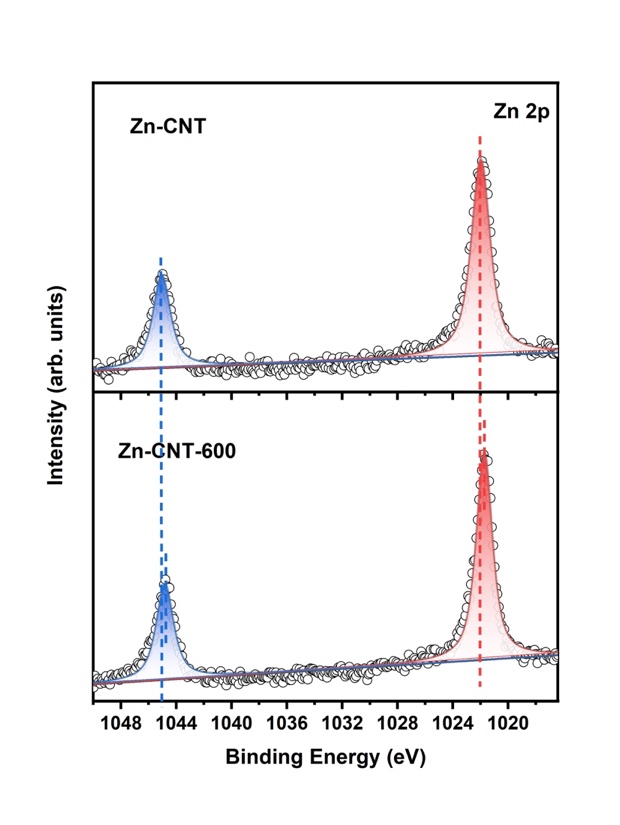


**Figure S42.** Zn 2p XPS spectra of Zn-CNT and Zn-CNT-600.


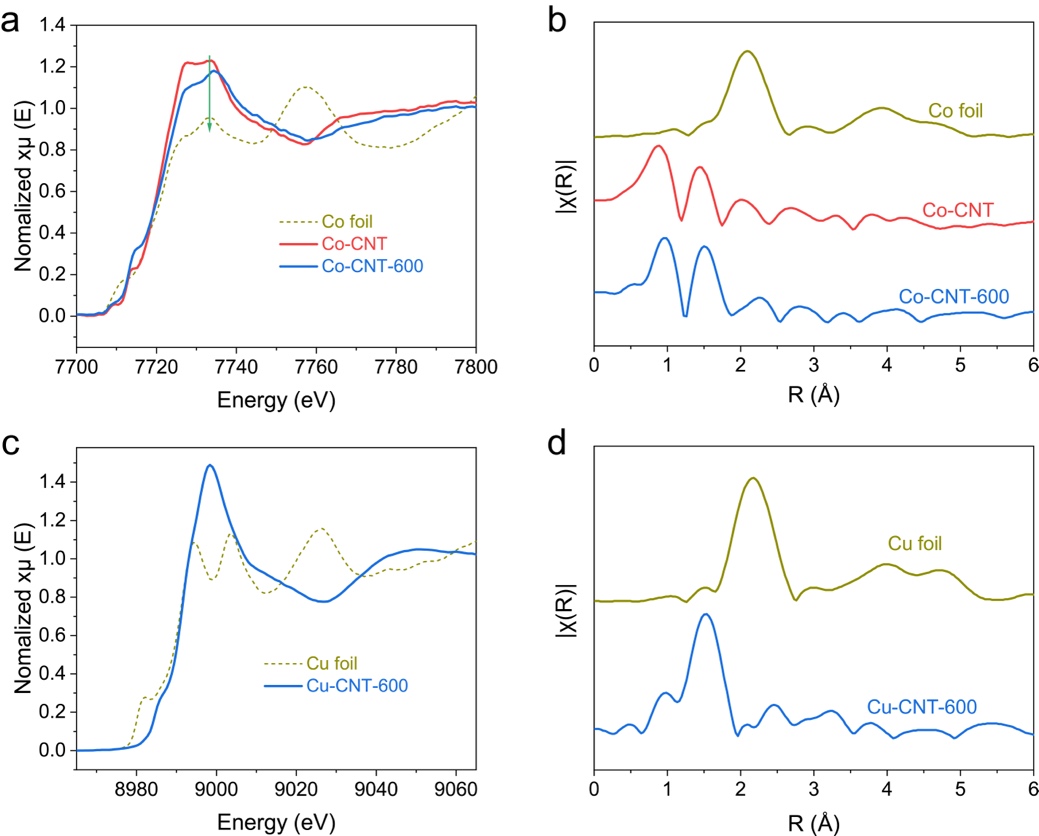


**Figure S43.** (a) Co K-edge XANES spectra and (b) The plotted Fourier transformation of the EXAFS spectra of Co foil, Co-CNT, and Co-CNT-600; (c) Cu K-edge XANES spectra and (d) The plotted Fourier transformation of the EXAFS spectra of Cu foil, and Cu-CNT-600.


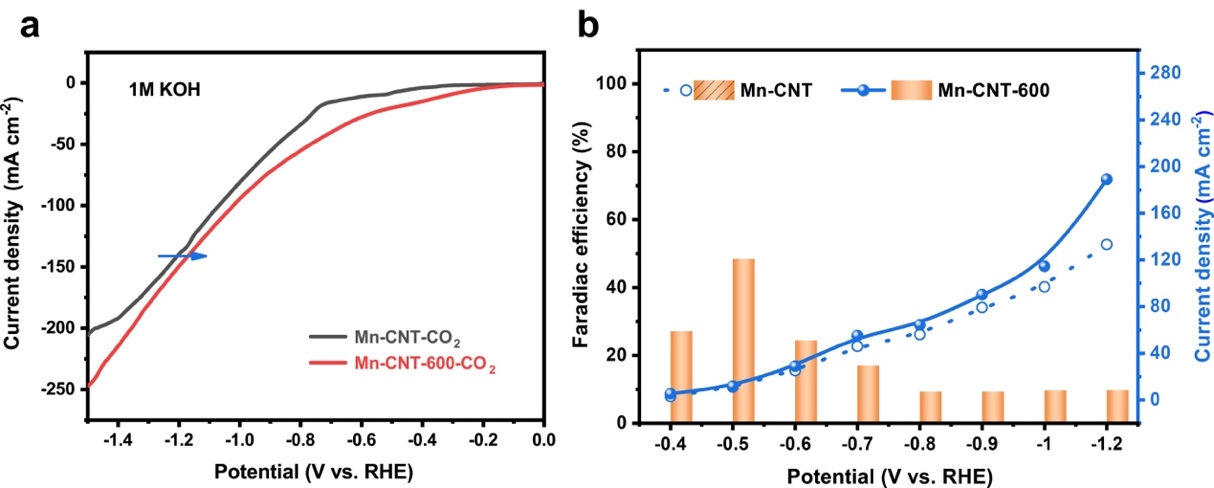


**Figure S44.** Electrochemical CO_2_ reduction performance in 1M KOH. (a) LSV curves of Mn-CNT and Mn-CNT-600; (b) Faradaic efficiency of CO and electrolysis current densities at various applied potentials for Mn-CNT and Mn-CNT-600.


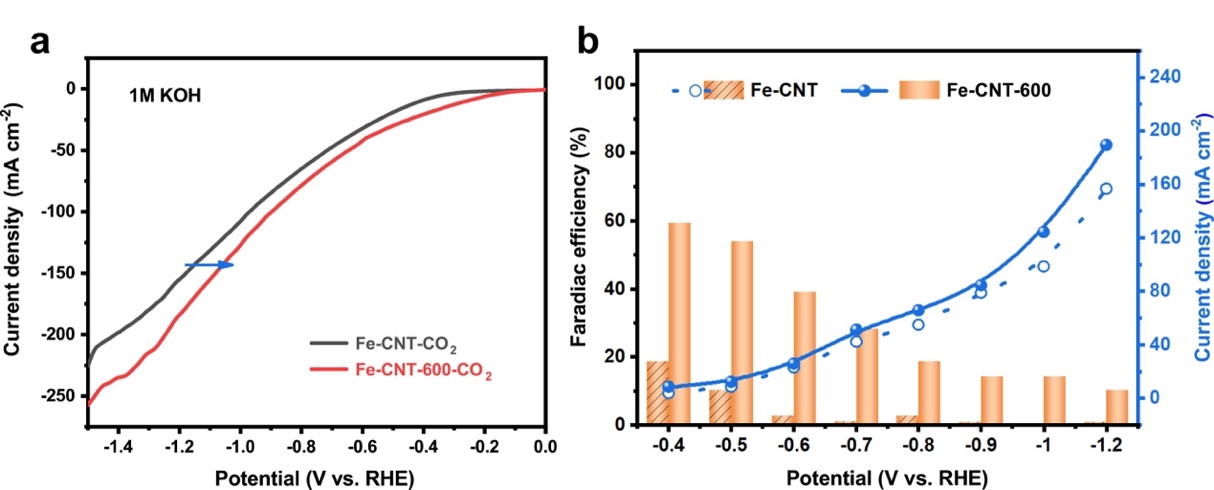


**Figure S45.** Electrochemical CO_2_ reduction performance in 1M KOH. (a) LSV curves of Fe-CNT and Fe-CNT-600; (b) Faradaic efficiency of CO and electrolysis current densities at various applied potentials for Fe-CNT and Fe-CNT-600.


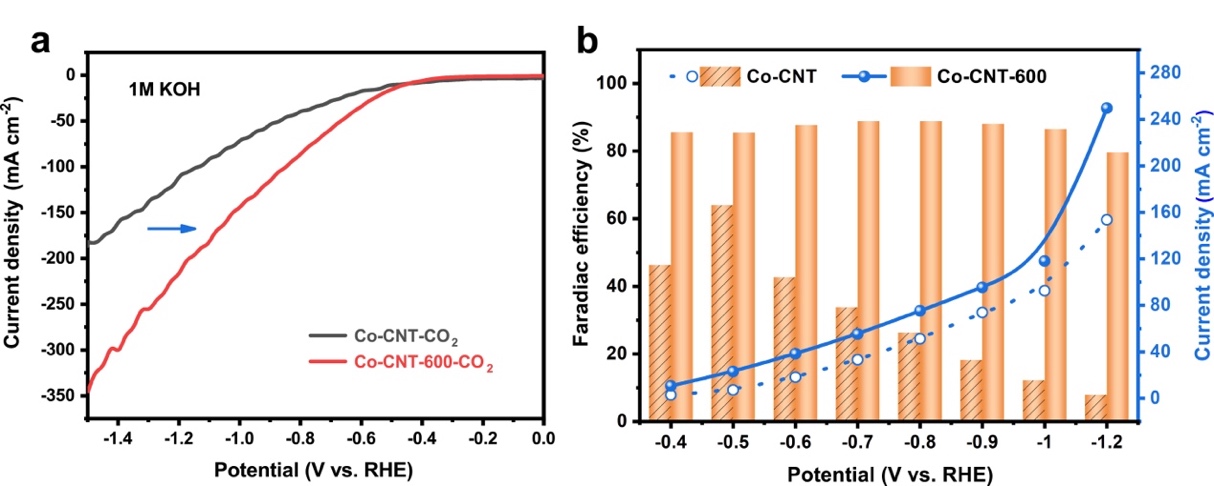


**Figure S46.** Electrochemical CO_2_ reduction performance in 1M KOH. (a) LSV curves of Co-CNT and Co-CNT-600; (b) Faradaic efficiency of CO and electrolysis current densities at various applied potentials for Co-CNT and Co-CNT-600.

**
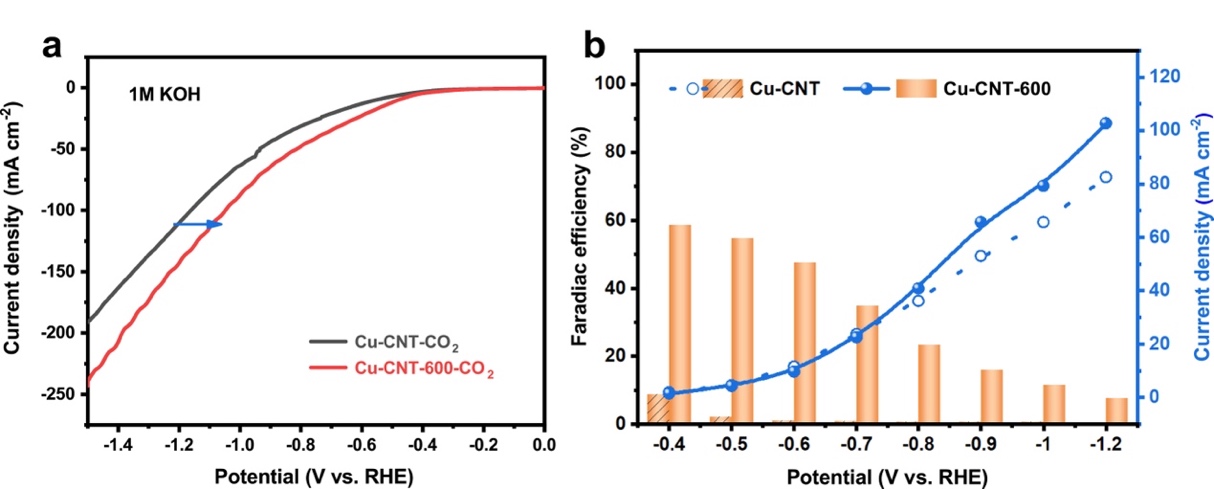
**

**Figure S47.** Electrochemical CO_2_ reduction performance in 1M KOH. (a) LSV curves of Cu-CNT and Cu-CNT-600; (b) Faradaic efficiency of CO and electrolysis current densities at various applied potentials for Cu-CNT and Cu-CNT-600.


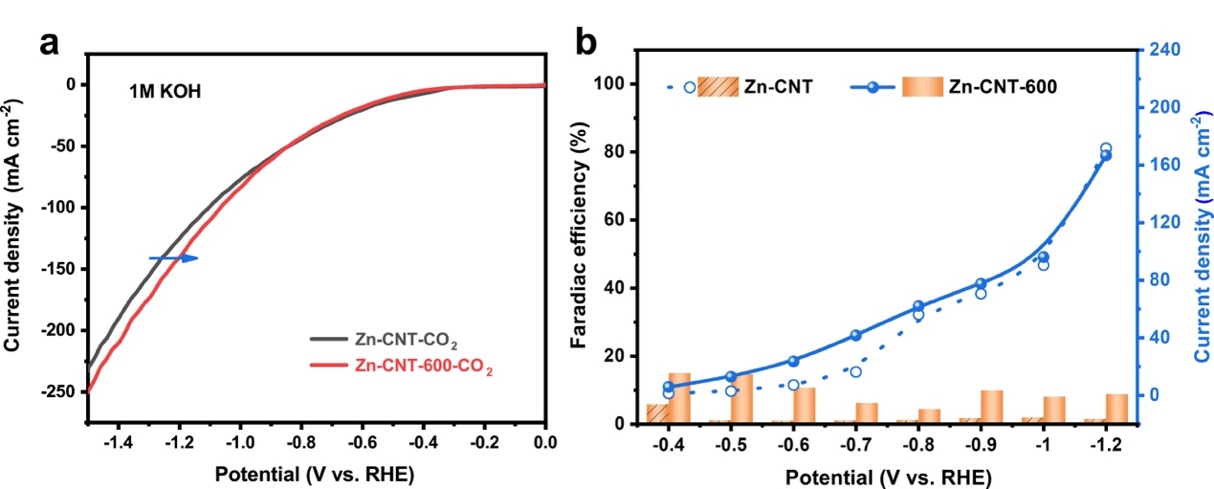


**Figure S48.** Electrochemical CO_2_ reduction performance in 1M KOH. (a) LSV curves of Zn-CNT and Zn-CNT-600; (b) Faradaic efficiency of CO and electrolysis current densities at various applied potentials for Zn-CNT and Zn-CNT-600.


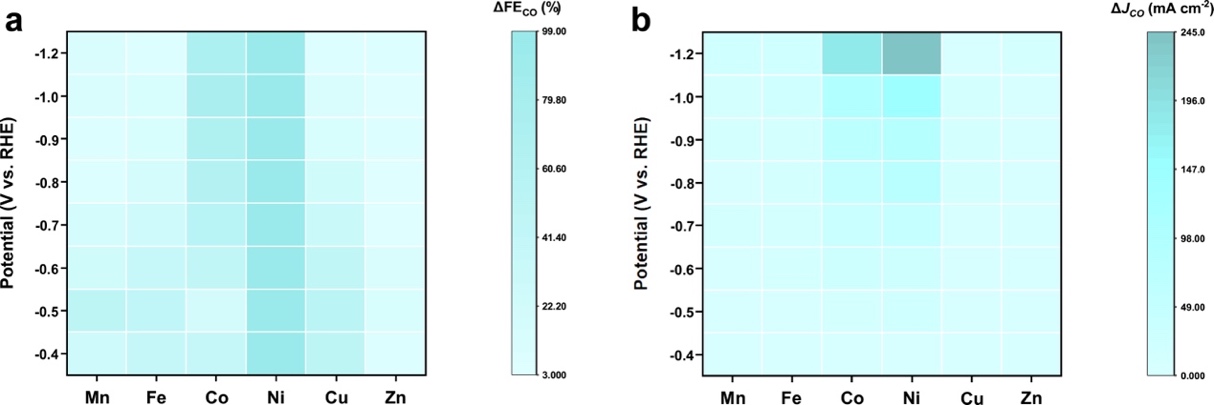


**Figure S49.** Comparison of the difference in CO Faradaic efficiency (ΔFE_CO_, a) and partial CO current density (Δ*J*_CO_, b) between M-CNT-600 and M-CNT (M = Mn, Fe, Co, Ni, Cu, Zn) under various applied potentials.

As shown in Figure S49, all calcined samples (M-CNT-600) exhibit enhanced CO_2_RR performance, validating the effectiveness of coordination asymmetry engineering. Among them, Co-CNT-600 also shows notable improvement, while Ni-CNT-600 displays the most pronounced enhancement, achieving the highest activity and product selectivity.


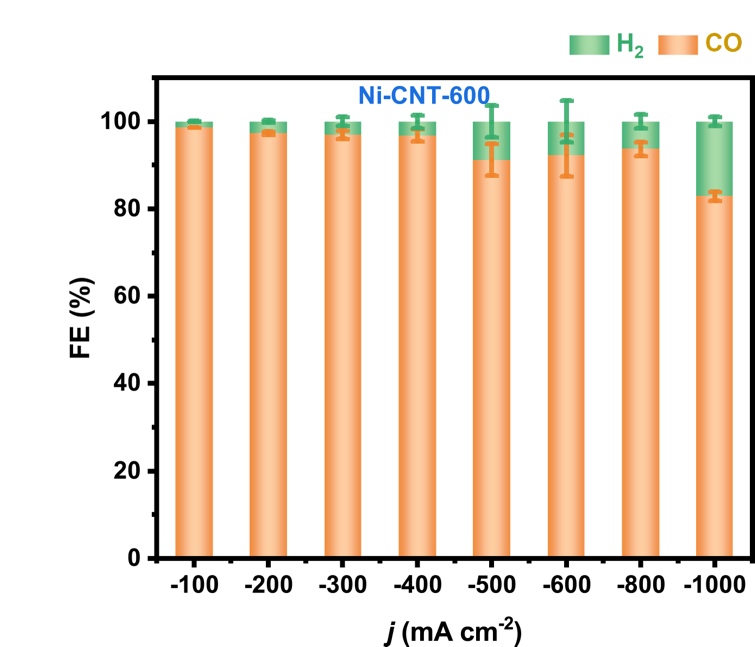


**Figure S50.** CO and H_2_ Faradaic efficiencies of Ni-CNT-600 in 1M KOH at -100 to -1000 mA cm^-2^.


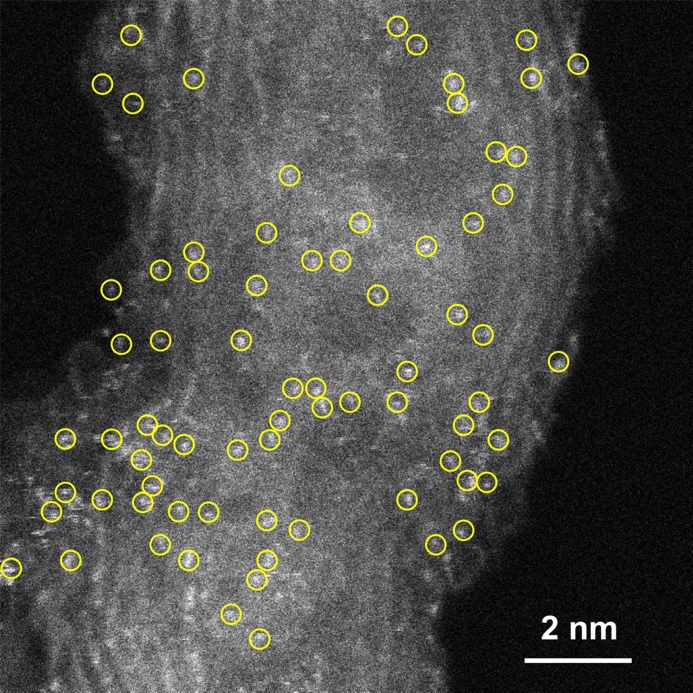


**Figure S51.** HAADF-STEM of used Ni-CNT-600.


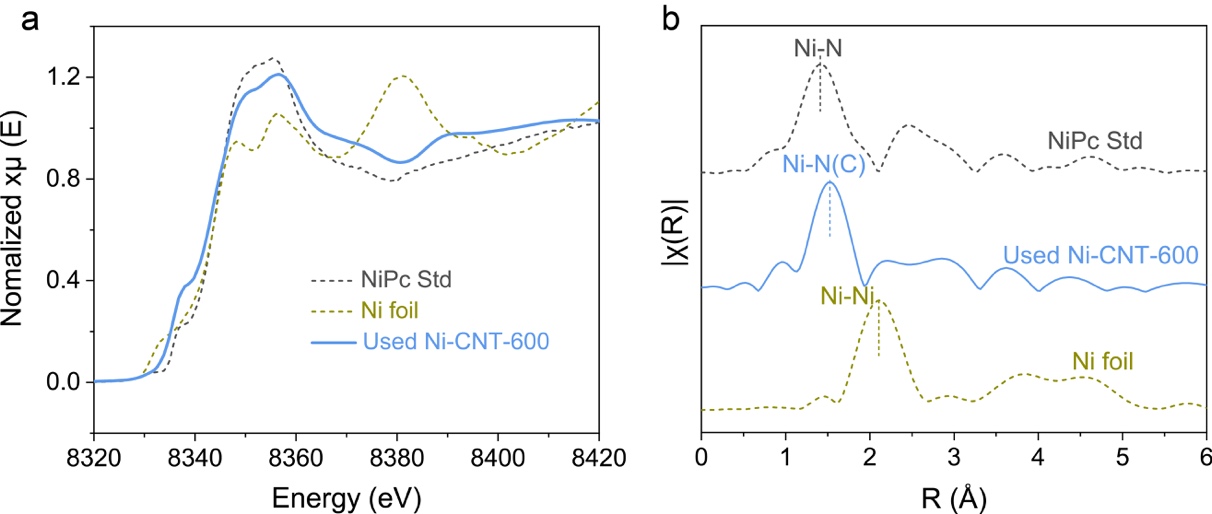


**Figure S52.** (a) Ni K-edge XANES spectra and (b) the plotted Fourier transformation of the extended X-ray absorption fine structure (FT-EXAFS) spectra of used Ni-CNT-600, NiPc and Ni foil.


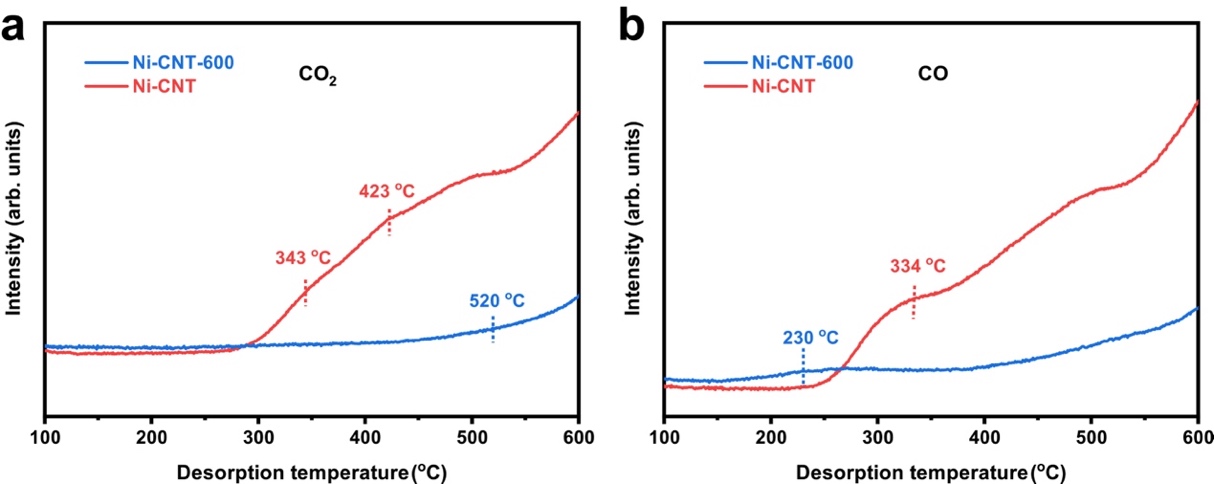


**Figure S53.** Temperature-programmed desorption (TPD) profiles of CO_2_ (a) and CO (b) for Ni-CNT and Ni-CNT-600.

As shown in Figure S53, distinct desorption features are observed for the two samples. Ni-CNT displays relatively low CO_2_ desorption peaks at 343 and 423 ^o^C together with a higher CO desorption temperature (334 ^o^C), indicating weak CO_2_ adsorption associated with symmetric NiN_4_ porphyrin sites. In contrast, Ni-CNT-600 exhibits a pronounced CO_2_ desorption peak at 520 ^o^C and a much lower CO desorption temperature (230 ^o^C), suggesting that asymmetric NiN_3_C sites strengthen CO_2_ adsorption while facilitating CO desorption, which is consistent with its superior CO_2_RR performance.


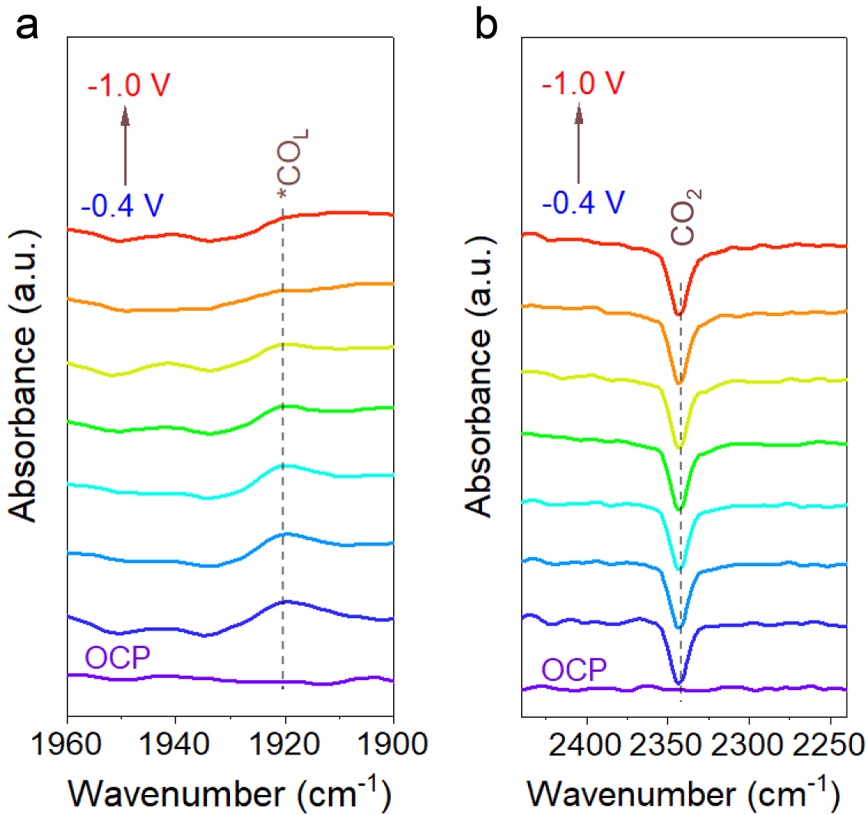


**Figure S54.** Enlarged *in situ* ATR-SEIRAS spectra of Ni-CNT-600 in the linearly adsorbed *CO region (a) and the CO_2_-related region (b), acquired at potentials ranging from OCP to -1.0 V vs. RHE.


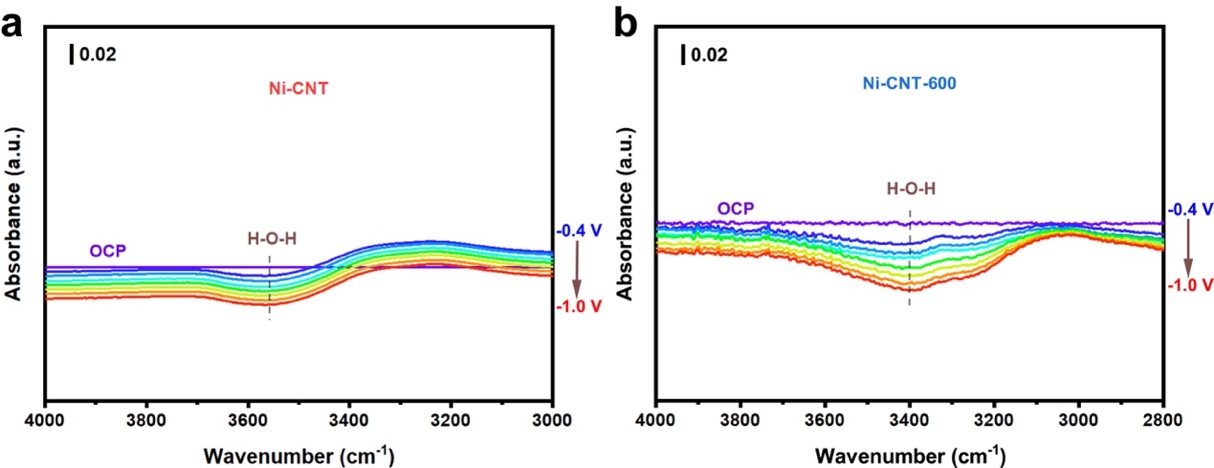


**Figure S55.** In situ ATR-SEIRAS spectra of Ni-CNT (a) and Ni-CNT-600 (b) recorded at potentials ranging from OCP to -1.0 V vs. RHE.

As shown in Figure S55, Ni-CNT exhibits almost no detectable changes in the H-O-H stretching region (~3550 cm^-1^) under applied potentials. In contrast, Ni-CNT-600 shows obvious intensity variations in the H-O-H stretching region spanning 3200–3600 cm^-1^, indicating enhanced interfacial water activation and a more favorable local environment for CO_2_ reduction.


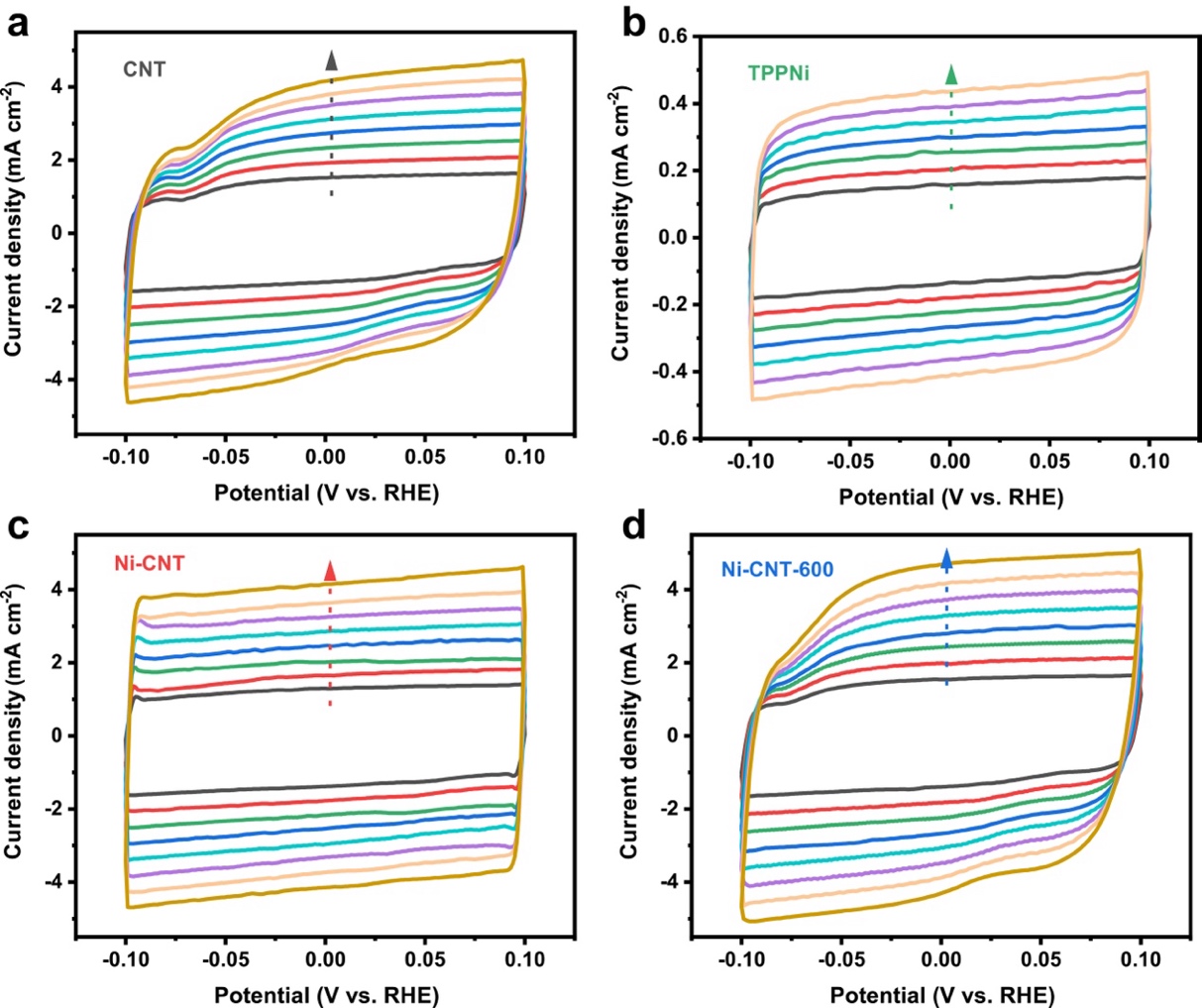


**Figure S56.** Cyclic voltammograms recorded at different scan rates (60–200 mV s^-1^) of (a) CNT, (b) TPPNi, (c) Ni-CNT and (d) Ni-CNT-600 measured in 1M KOH flow cells.


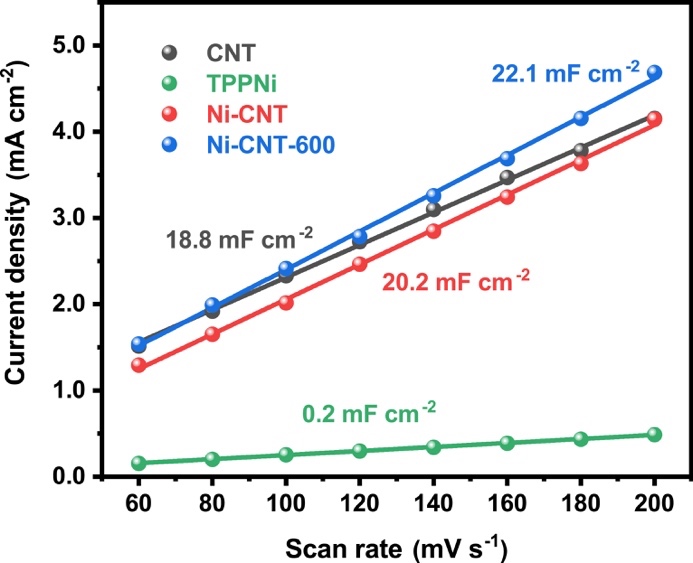


**Figure S57.** Double-layer capacitance (C_dl_) of CNT, TPPNi, Ni-CNT and Ni-CNT-600 measured in 1M KOH.


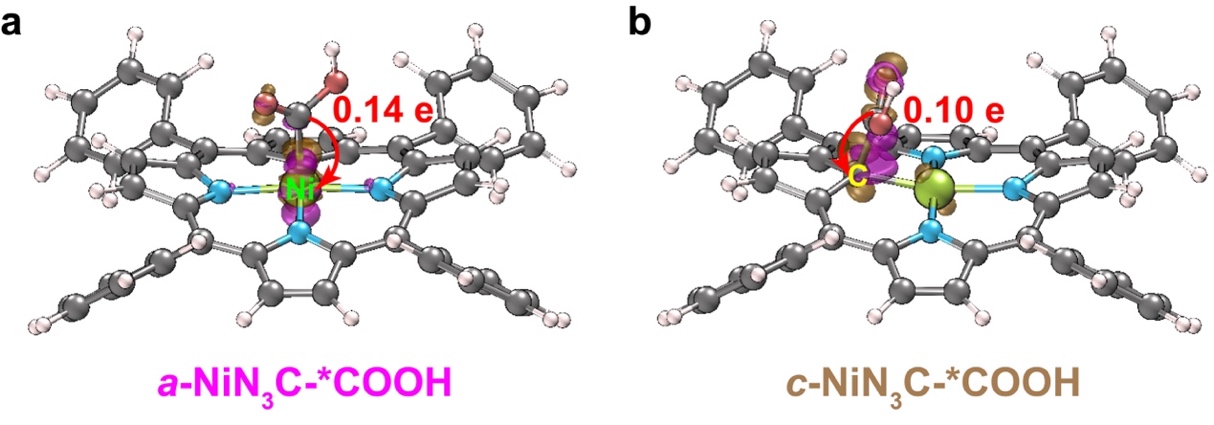


**Figure S58.** Charge density difference analysis between catalytic active sites and the *COOH intermediate for *a*-NiN_3_C (a) and *c*-NiN_3_C (b) models. Purple regions indicate electron density accumulation, while brown regions represent electron density depletion.

Charge-density analysis reveals a clear evolution in electron-transfer behavior across different coordination environments. For NiN_4_–*COOH, the largest net electron transfer (0.16 e) from COOH to the Ni center is observed, indicating excessively strong Ni-centered σ interactions, which suppresses the essential back-donation to the COOH intermediate and hinders subsequent CO formation. Upon introducing asymmetry via N-to-C substitution, the *a*-NiN_3_C–*COOH configuration exhibits a reduced transfer of 0.14 e, partially mitigating Ni-centered σ interactions, yet the Ni site remains relatively electron-rich. By contrast, *c*-NiN_3_C–*COOH shows the lowest charge transfer (0.10 e), which effectively suppresses over-donation from *COOH and enables more effective C-centered π coupling with *COOH, thereby promoting C–O bond activation and CO desorption. As a result, the *c*-NiN_3_C site achieves the most favorable electronic balance for CO_2_ reduction, followed by *a*-NiN_3_C, whereas NiN_4_ suffers from overly strong binding and limited catalytic turnover.


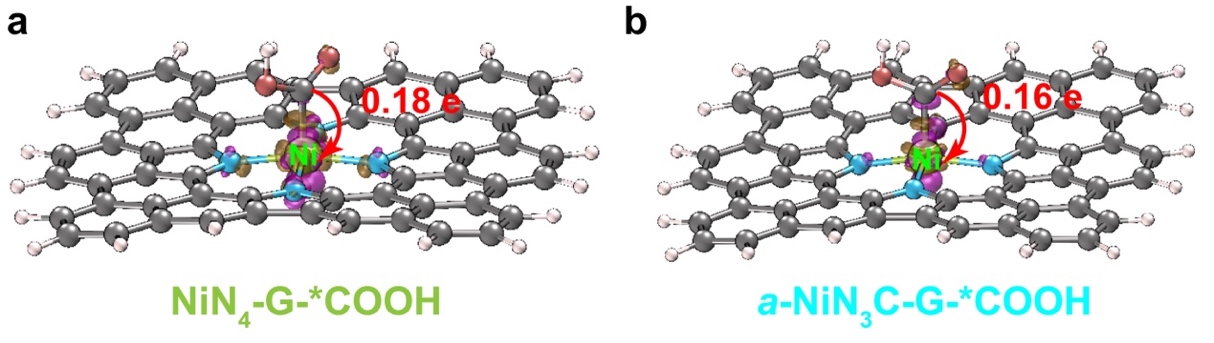


**Figure S59.** Charge density difference analysis between catalytic active sites and the *COOH intermediate for NiN_4_-G (a) and *a*-NiN_3_C-G (b) models. Purple region indicates electron density accumulation, while brown regions represent electron density depletion.

For NiN_4_-G–*COOH, the charge transfer from *COOH to Ni-site is as high as 0.18 e, indicating excessive electron donation that suppresses Ni-to-COOH back-donation and leads to poor CO_2_RR performance. In *a*-NiN_3_C-G–*COOH, the charge transfer decreased slightly to 0.16 e, partially alleviating over-binding, yet the Ni-centered σ interactions remains predominantly donation-driven. By contrast, *c*-NiN_3_C-G–*COOH exhibits nearly zero net charge transfer (0.01 e, reversed toward *COOH), which minimizes over-donation and optimizes CO formation, consistent with its superior catalytic activity.


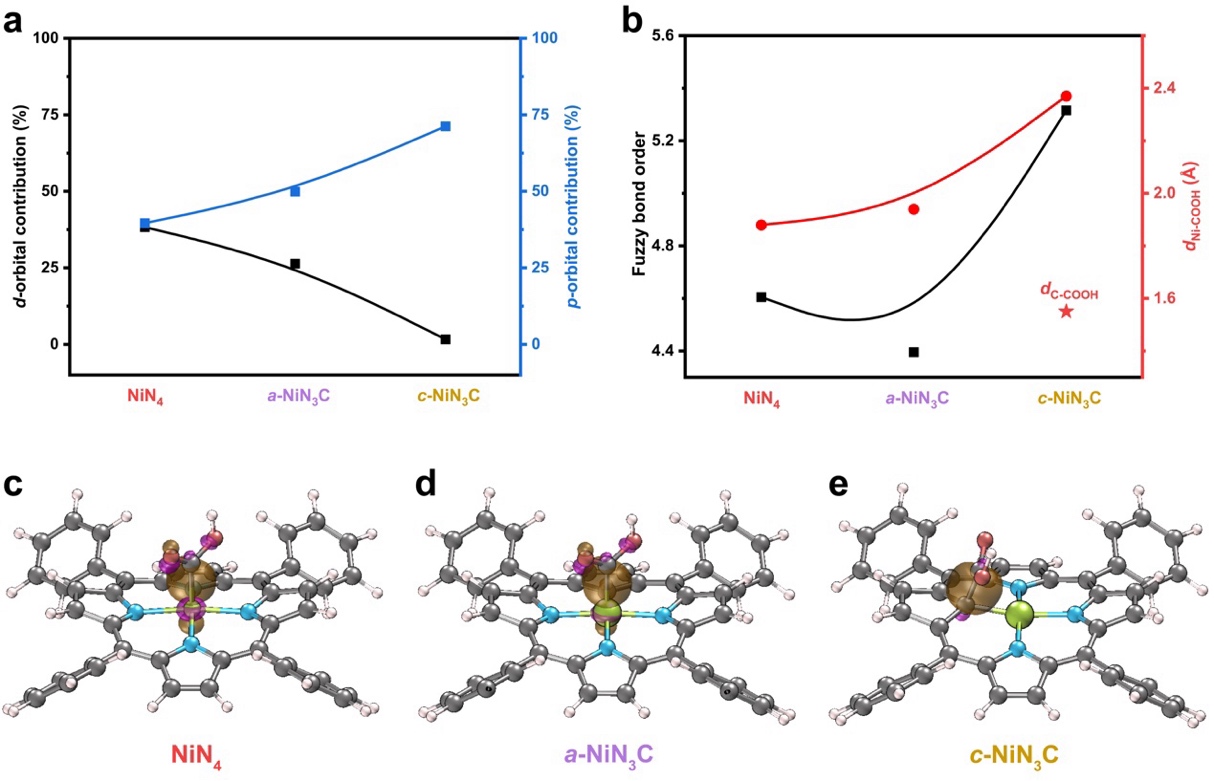


**Figure S60.** (a) Orbital-decomposed analysis illustrating the evolving contributions of Ni *d* and *COOH *p* orbitals across NiN_4_, *a*-NiN_3_C, and *c*-NiN_3_C models; (b) Comparison of fuzzy bond order and Ni–*COOH bond length, where the red star denotes the C–*COOH bond length, highlighting coordination-dependent bonding strength and intermediate stabilization; Predominating natural adaptive orbital (NAdO) visualizations for (c) NiN_4_, (d) *a*-NiN_3_C, and (e) *c*-NiN_3_C, illustrating differences in orbital overlap with the *COOH intermediate. Purple regions indicate electron density accumulation, whereas brown regions represent electron density depletion.

Fuzzy atomic space and NAdO analyses reveal that both NiN_4_ and *a*-NiN_3_C involve substantial hybridization between Ni *d* and *COOH *p* orbitals, indicative of Ni-centered COOH binding. By contrast, *c*-NiN_3_C exhibits negligible Ni *d* orbital contribution and is dominated by *COOH *p* orbital participation, suggesting that *COOH preferentially adsorbs at the adjacent C site rather that the Ni center. Consistently, the *c*-NiN_3_C–*COOH configuration shows the superior fuzzy bond order and shortest C–COOH bond length (relative to Ni–COOH), reflecting strengthened C-centered binding and optimal intermediate stabilization, which is in line with its highest catalytic activity.


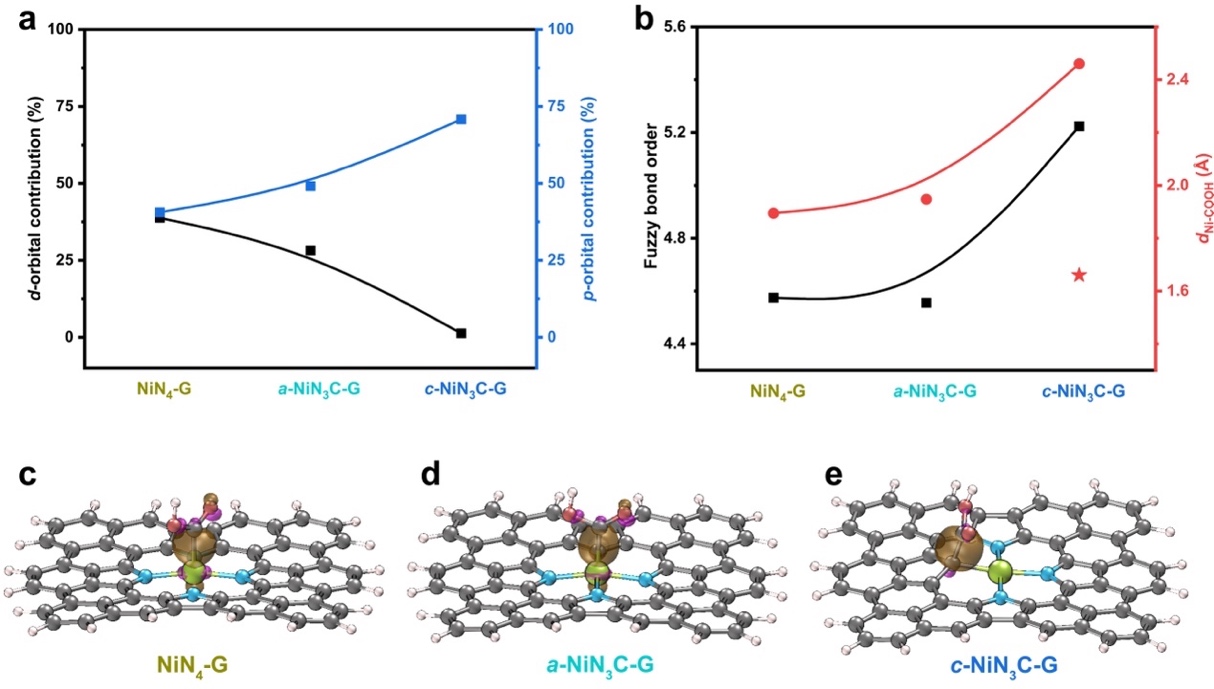


**Figure S61.** (a) Orbital-decomposed analysis illustrating the evolving contributions of Ni *d* and *COOH *p* orbitals across NiN_4_-G, *a*-NiN_3_C-G, and *c*-NiN_3_C-G models; (b) Comparison of fuzzy bond order and Ni–*COOH bond length, where the red star denotes the C–*COOH bond length, highlighting coordination-dependent bonding strength and intermediate stabilization; NAdO visualizations for (c) NiN_4_-G, (d) *a*-NiN_3_C-G, and (e) *c*-NiN_3_C-G, illustrating differences in orbital overlap with the *COOH intermediate. Purple regions indicate electron density accumulation, while brown regions represent electron density depletion.

Fuzzy atomic space and NAdO analyses demonstrate that NiN_4_-G and *a*-NiN_3_C-G involved both Ni *d* orbital and *COOH *p* orbital in bonding, whereas *c*-NiN_3_C-G shows negligible Ni *d* orbital contribution with predominant *COOH *p* orbital participation. Thus, *COOH preferentially adsorbs on the C site of *c*-NiN_3_C-G. The *c*-NiN_3_C-G–*COOH configuration exhibits a higher fuzzy bond order and a shorter C-COOH bond length, thereby achieving the highest catalytic activity.


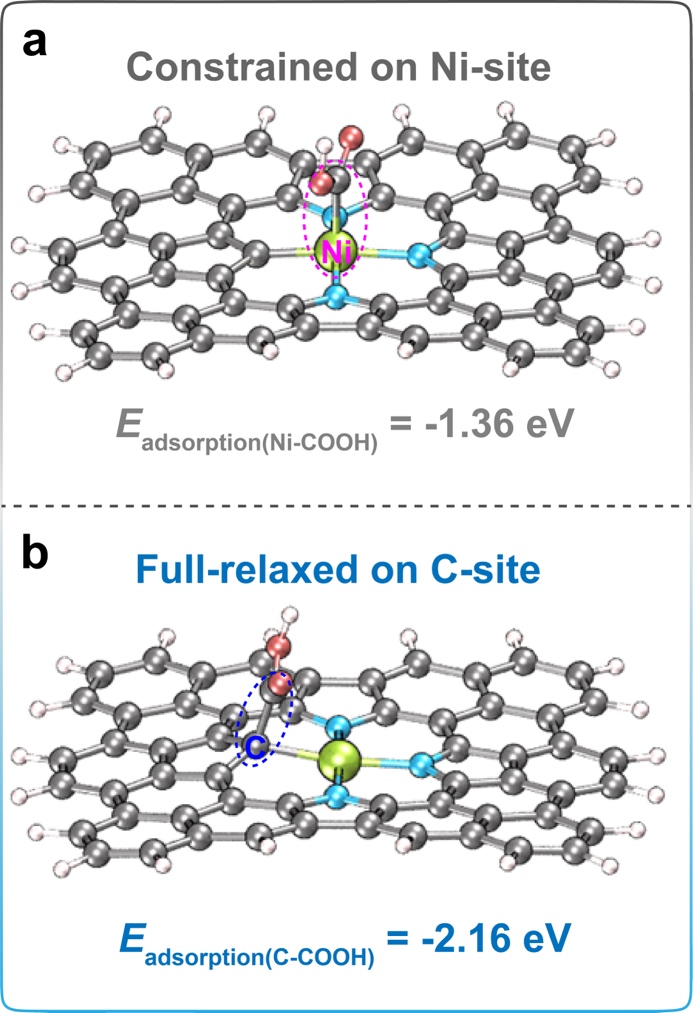


**Figure S62.** Different adsorption-site configurations of *COOH on the *c*-NiN_3_C-G model (corresponding to Ni-CNT-600 catalyst): (a) constrained on the Ni-site with an adsorption energy of -1.36 eV and (b) fully relaxed on the adjacent C-site with an adsorption energy of -2.16 eV.

The stronger binding energy on the C-site indicates that *COOH preferentially adsorbs at the adjacent carbon rather than directly on the Ni site, consistent with the unique reactivity of the asymmetric *c*-NiN_3_C-G motif.


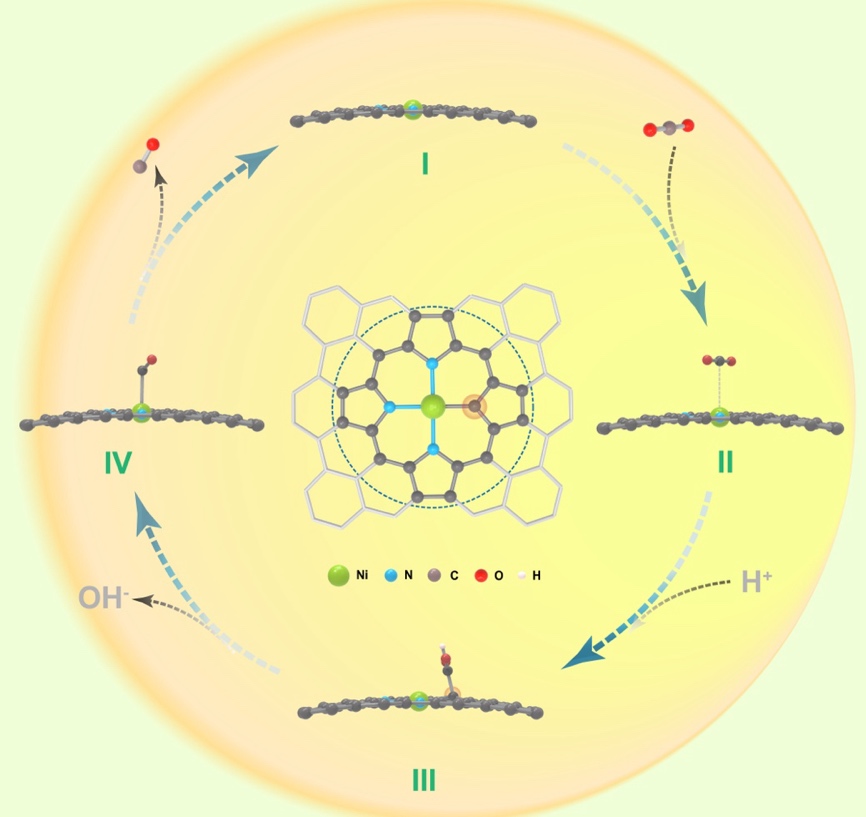


**Figure S63.** The reaction pathway of CO_2_-to-CO for Ni-CNT-600.


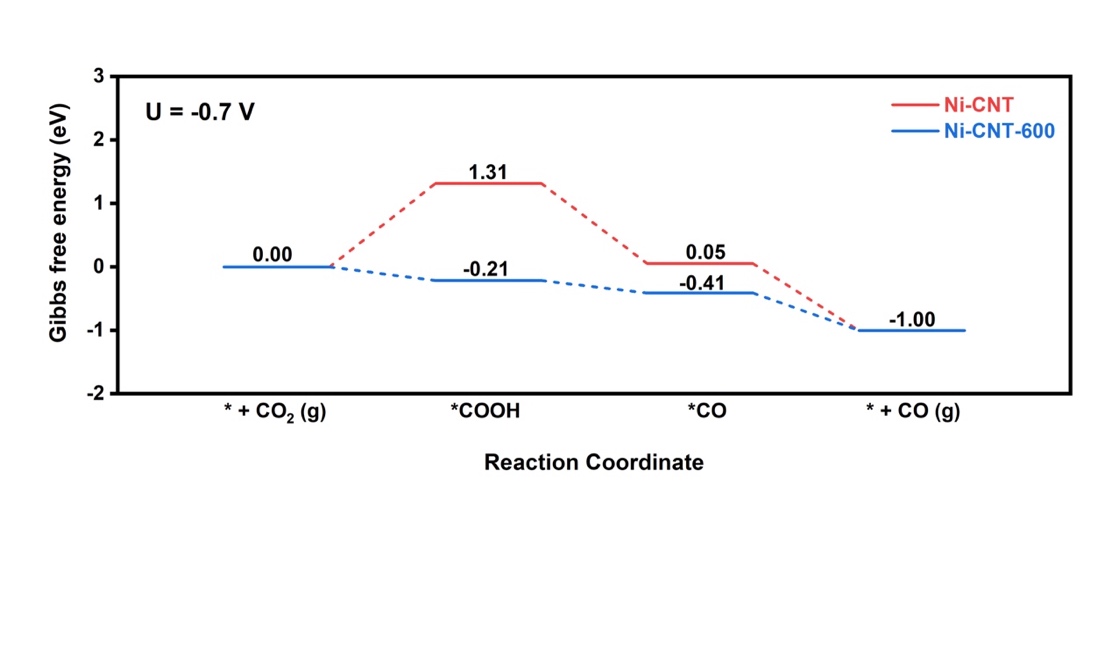


**Figure S64.** Reaction pathways of CO_2_RR over Ni-CNT and Ni-CNT-600 at U = -0.7 V.

As shown in Figure S64 (U = -0.7 V), Ni-CNT-600 exhibits a much lower energy barrier for *COOH formation and more favorable energetics for *CO stabilization and desorption compared with Ni-CNT, accounting for its superior CO_2_RR activity.


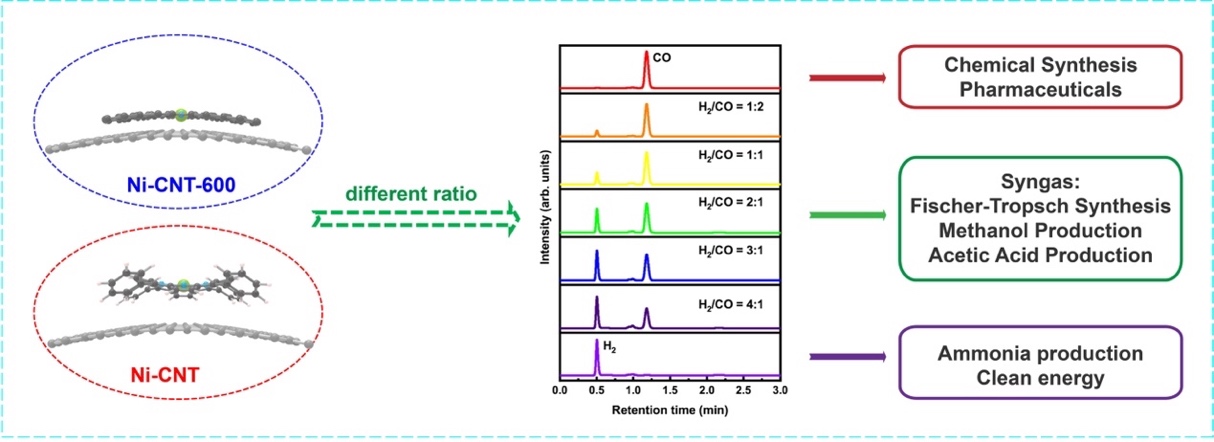


**Figure S65.** Application of the asymmetric selectivity-switching Ni-CNT/Ni-CNT-600 system.

By introducing different ratios of symmetric Ni-CNT (HER-dominant) and asymmetric Ni-CNT-600 (CO_2_RR-dominant) catalysts, the product distribution between H_2_ and CO can be continuously tuned, yielding H_2_/CO ratios ranging from 1:2 to 4:1. This controllable pathway switching enables tailored syngas compositions for diverse applications, including CO-rich chemical synthesis, balanced syngas for Fischer-Tropsch and methanol production, and H_2_-rich streams for ammonia synthesis or clean energy applications. Notably, this strategy also allows the selective output of nearly pure H_2_ or pure CO, consistent with the near-unity selectivity inversion described in the main text.

**Table S1.** ICP results of Ni-CNT and Ni-CNT-600.

| Catalysts | wt % of Ni |
| --- | --- |
| Ni-CNT | 0.39 |
| Ni-CNT-600 | 0.42 |

**Table S2.** EXAFS fitting parameters at the Ni K-edge for Ni-CNT and Ni-CNT-600.

| Catalysts | Shell | CN | R (Å) | σ^2^ | R factor |
| --- | --- | --- | --- | --- | --- |
| Ni-CNT | Ni-N | 3.9±0.1 | 1.870 | 0.004 | 0.02 |
| Ni-CNT-600 | Ni-C | 0.9±0.1 | 1.879 | 0.001 | 0.015 |
|  | Ni-N | 3.7±0.2 | 1.937 | 0.001 |  |

*^a^*CN: coordination numbers; *^b^*R: bond distance; *^c^*σ^2^: Debye-Waller factors; *^d^*R factor: goodness of fit.

**Table S3.** The calculated bond distance of Ni-N(C) according to optimized NiN_4_ and *c*-NiN_3_C-G structures.

|  | NiN_4_-model | *c*-NiN_3_C-G-model |
| --- | --- | --- |
| R_1_ (Ni-N_1_, Å) | 1.918 | 2.012 |
| R_2_ (Ni-N_2_ or Ni-C, Å) | 2.014 | 1.960 |
| R_3_ (Ni-N_3_, Å) | 2.020 | 2.021 |
| R_4_ (Ni-N_4_, Å) | 1.922 | 2.043 |
| R_average_ (Ni-N, Å) | 1.968 | 2.025 |

R: bond distance; R1 and R4 represent the nitrogen atom along the axial direction of the CNT; R2 and R3 represent the nitrogen or substituted carbon atom along the circumferential direction.

**Table S4.** Comparative analysis of single atom catalysts for CO_2_RR performance.

| Entry | Catalysts | TOF (h^-1^) | FE_CO_ (%) | Stability (h) | References |
| --- | --- | --- | --- | --- | --- |
| 1 | Ni-CNT-600 | 6.03$\times$10^5^ | 98.7*^a^* | 105 | This Work |
|  |  | 5.90$\times$10^4^ | 99.5*^b^* |  |  |
|  |  | 1.42$\times$10^5^ | 97.3*^c^* |  |  |
|  |  | *^d^*4.76$\times$10^5^ | 98.7*^a^* |  |  |
| 2 | Ni_1_-N/CNT | 3.8$\times$10^4^ | 100 | 60 | *Angew. Chem. Int. Ed.,* 2020, **59**, 21885.^1^ |
| 3 | NiPc-TFPN COF | 490 | 99.8 | 60 | *Angew. Chem. Int. Ed.,* 2021, **60**, 4864.^2^ |
| 4 | Ni^I^-NCNT@Ni_9_Cu | 1962 | 93 | 20 | *Angew. Chem. Int. Ed.*, 2020, **59**, 12055.^3^ |
| 5 | NiPc-OMe MDE | 4.32$\times$10^4^ | 99.5 | 40 | *Nat. Energy*, 2020, **5**, 684.^4^ |
| 6 | Ni-CNT-CC | 1.00$\times$10^5^ | 99 | 100 | *Angew. Chem. Int. Ed.*, 2020, **59**, 798.^5^ |
| 7 | FeTbPP/CFP | 7.20$\times$10^4^ | 100 | 2 | *Angew. Chem. Int. Ed.,* 2024, **63**, e202412188.^6^ |
| 8 | H-NiPc/CNT | 1.38$\times$10^4^ | 97 | 16 | *ACS Catal.*, 2020, **10**, 10920.^7^ |
| 9 | Ni-SAC@NFC | 9.50$\times$10^4^ | 95 | 14 | *ACS Nano*, 2024, **18**, 26751.^8^ |
| 10 | NiFe-N-bridge | 976 | 83 | 20 | *Angew. Chem. Int. Ed.,* 2024, **63**, e202411543.^9^ |
| 11 | InNi DS/NC | 7353 | 90 | 10 | *Angew. Chem. Int. Ed.,* 2023, **62**, e202216326.^10^ |
| 12 | Ni-N-C | 2.74$\times$10^5^ | 90 | 60 | *Nat. Commun.*, 2023, **14**, 3776.^11^ |
| 13 | Ni@C_3_N_4_-CN | 2.20$\times$10^4^ | 90 | 20 | *Nat. Commun.*, 2022, **13**, 6082.^12^ |
| 14 | ZIF-NC-Ni-Fe | 2210 | 97.8 | 50 | *Angew. Chem. Int. Ed.,* 2022, **61**, e202210985.^13^ |
| 15 | Co-CNTs-MW | 2.59$\times$10^4^ | 96 | 100 | *Nat. Commun.*, 2023, **14**, 1599.^14^ |
| 16 | Ni_2_NC | 77500 | 99 | 30 | *Nat. Synth.*, 2022, **1**, 719.^15^ |
| 17 | Ni-SAC-250 | 2.52$\times$10^4^ | 99 | 25 | *Nat. Commun.*, 2024, **15**, 1719.^16^ |
| 18 | Ni-N_pyridinic_-C | ~1.3$\times$10^4^ | 99 | 45 | *Angew. Chem. Int. Ed.,* 2025, **64**, e202506845.^17^ |
| 19 | NiPc-NH_2_/ CNT-SHP | 63936 | 100 | 48 | *Nat. Commun.*, 2025, **16**, 8704.^18^ |
| 20 | Ni-NBr-C | ~3.6$\times$10^4^ | 97 | 85 | *Angew. Chem. Int. Ed.,* 2025, **64**, e202414569.^19^ |
| 21 | NiTPP@CNT-450 | ~2.3$\times$10^4^ | 98.3 | 40 | *Angew. Chem. Int. Ed.,* 2025, **64**, e202506663.^20^ |
| 22 | Ni_6_@NiN_3_ | 83984.2 | 99.7 | 2h | *Angew. Chem. Int. Ed.,* 2025, **64**, e202424552.^21^ |
| 23 | Fe-S_1_N_3_ | 7804.34 | 99 | 40 | *Angew. Chem. Int. Ed.,* 2024, **63**, e202318246.^22^ |
| 24 | Al-NC | 12960 | 98.8 | 16 | *J. Am. Chem. Soc.,* 2024, **146**, 29140.^23^ |

*^a^*1M KOH; *^b^*0.1 M KHCO_3_; *^c^*0.05 M H_2_SO_4_ + 0.5 M Na_2_SO_4_; *^d^*TOF*_SAS_*.

**Reference:**

(1) Jin, S.; Ni, Y.; Hao, Z.; Zhang, K.; Lu, Y.; Yan, Z.; Wei, Y.; Lu, Y. R.; Chan, T. S.; Chen, J. A Universal Graphene Quantum Dot Tethering Design Strategy to Synthesize Single-Atom Catalysts. *Angew. Chem. Int. Ed.* **2020**, *59* (49), 21885-21889.

(2) Lu, M.; Zhang, M.; Liu, C. G.; Liu, J.; Shang, L. J.; Wang, M.; Chang, J. N.; Li, S. L.; Lan, Y. Q. Stable Dioxin-Linked Metallophthalocyanine Covalent Organic Frameworks (COFs) as Photo-Coupled Electrocatalysts for CO_2_ Reduction. *Angew. Chem. Int. Ed.* **2021**, *60* (9), 4864-4871.

(3) Zhang, T.; Han, X.; Yang, H.; Han, A.; Hu, E.; Li, Y.; Yang, X. Q.; Wang, L.; Liu, J.; Liu, B. Atomically Dispersed Nickel(I) on an Alloy-Encapsulated Nitrogen-Doped Carbon Nanotube Array for High-Performance Electrochemical CO_2_ Reduction Reaction. *Angew. Chem. Int. Ed.* **2020**, *59* (29), 12055-12061.

(4) Zhang, X.; Wang, Y.; Gu, M.; Wang, M.; Zhang, Z.; Pan, W.; Jiang, Z.; Zheng, H.; Lucero, M.; Wang, H.; et al. Molecular engineering of dispersed nickel phthalocyanines on carbon nanotubes for selective CO_2_ reduction. *Nat. Energy* **2020**, *5* (9), 684-692.

(5) Liu, S.; Yang, H. B.; Hung, S. F.; Ding, J.; Cai, W.; Liu, L.; Gao, J.; Li, X.; Ren, X.; Kuang, Z.; et al. Elucidating the Electrocatalytic CO_2_ Reduction Reaction over a Model Single-Atom Nickel Catalyst. *Angew. Chem. Int. Ed.* **2020**, *59* (2), 798-803.

(6) Luo, Z. M.; Wang, J. W.; Nicaso, M.; Gil-Sepulcre, M.; Solano, E.; Nikolaou, V.; Benet, J.; Segado-Centellas, M.; Bo, C.; Llobet, A. Supramolecular Anchoring of Fe(III) Molecular Redox Catalysts into Graphitic Surfaces Via CH-pi and pi-pi Interactions for CO_2_ Electroreduction. *Angew. Chem. Int. Ed.* **2024**, *63* (46), e202412188.

(7) Sa, Y. J.; Jung, H.; Shin, D.; Jeong, H. Y.; Ringe, S.; Kim, H.; Hwang, Y. J.; Joo, S. H. Thermal Transformation of Molecular Ni^2+^–N_4_ Sites for Enhanced CO_2_ Electroreduction Activity. *ACS Catal.* **2020**, *10* (19), 10920-10931.

(8) Wang, Y.; Zhu, P.; Wang, R.; Matthews, K. C.; Xie, M.; Wang, M.; Qiu, C.; Liu, Y.; Zhou, H.; Warner, J. H.; et al. Fluorine-Tuned Carbon-Based Nickel Single-Atom Catalysts for Scalable and Highly Efficient CO_2_ Electrocatalytic Reduction. *ACS Nano* **2024**, *18* (39), 26751-26758.

(9) Chen, Y.; Zhao, J.; Pan, X.; Li, L.; Yu, Z.; Wang, X.; Ma, T.; Lin, S.; Lin, J. Tuning the Inter-Metal Interaction between Ni and Fe Atoms in Dual-Atom Catalysts to Boost CO_2_ Electroreduction. *Angew. Chem. Int. Ed.* **2024**, *63* (44), e202411543.

(10) Fan, Z.; Luo, R.; Zhang, Y.; Zhang, B.; Zhai, P.; Zhang, Y.; Wang, C.; Gao, J.; Zhou, W.; Sun, L.; et al. Oxygen-Bridged Indium-Nickel Atomic Pair as Dual-Metal Active Sites Enabling Synergistic Electrocatalytic CO_2_ Reduction. *Angew. Chem. Int. Ed.* **2023**, *62* (7), e202216326.

(11) Zhou, Y.; Zhou, Q.; Liu, H.; Xu, W.; Wang, Z.; Qiao, S.; Ding, H.; Chen, D.; Zhu, J.; Qi, Z.; et al. Asymmetric dinitrogen-coordinated nickel single-atomic sites for efficient CO_2_ electroreduction. *Nat. Commun.* **2023**, *14* (1), 3776.

(12) Wang, Q.; Liu, K.; Hu, K.; Cai, C.; Li, H.; Li, H.; Herran, M.; Lu, Y. R.; Chan, T. S.; Ma, C.; et al. Attenuating metal-substrate conjugation in atomically dispersed nickel catalysts for electroreduction of CO_2_ to CO. *Nat. Commun.* **2022**, *13* (1), 6082.

(13) Huang, J. R.; Qiu, X. F.; Zhao, Z. H.; Zhu, H. L.; Liu, Y. C.; Shi, W.; Liao, P. Q.; Chen, X. M. Single-Product Faradaic Efficiency for Electrocatalytic of CO_2_ to CO at Current Density Larger than 1.2 A cm^-2^ in Neutral Aqueous Solution by a Single-Atom Nanozyme. *Angew. Chem. Int. Ed.* **2022**, *61* (44), e202210985.

(14) Sun, J. W.; Wu, X.; Liu, P. F.; Chen, J.; Liu, Y.; Lou, Z. X.; Zhao, J. Y.; Yuan, H. Y.; Chen, A.; Wang, X. L.; et al. Scalable synthesis of coordinatively unsaturated metal-nitrogen sites for large-scale CO_2_ electrolysis. *Nat. Commun.* **2023**, *14* (1), 1599.

(15) Hao, Q.; Zhong, H.-x.; Wang, J.-z.; Liu, K.-h.; Yan, J.-m.; Ren, Z.-h.; Zhou, N.; Zhao, X.; Zhang, H.; Liu, D.-x.; et al. Nickel dual-atom sites for electrochemical carbon dioxide reduction. *Nature Synthesis* **2022**, *1* (9), 719-728.

(16) Wang, B.; Wang, M.; Fan, Z.; Ma, C.; Xi, S.; Chang, L. Y.; Zhang, M.; Ling, N.; Mi, Z.; Chen, S.; et al. Nanocurvature-induced field effects enable control over the activity of single-atom electrocatalysts. *Nat. Commun.* **2024**, *15* (1), 1719.

(17) Chen, Z.; Liu, J.; Li, J.; Zhang, Y.; Yang, J.; Li, J.; Wang, Z.; Liu, Z.; Zang, S. Q. Modulating Spin State of Ni Single Atomic Center for High-Performance Electrocatalytic Carbon Dioxide Reduction. *Angew. Chem. Int. Ed.* **2025**, *64* (33), e202506845.

(18) Gong, S.; Zhai, Y.; Jin, C.; Xu, H.; Xia, Q.; Li, W.; Ying, Y.; Wu, J.; She, X.; Wang, Z.; et al. Interface engineering of single-molecular heterojunction catalysts for CO_2_ electroreduction in strong acid medium. *Nat. Commun.* **2025**, *16* (1), 8704.

(19) Lin, Y.; Xia, C.; Zhu, Z.; Wang, J.; Niu, H.; Gong, S.; Li, Z.; Yang, N.; Song Chen, J.; Wu, R.; et al. Carbon Nanocage Supported Asymmetrically Coordinated Nickle Single-Atom for Enhanced CO_2_ Electroreduction in Membrane Electrode Assembly. *Angew. Chem. Int. Ed.* **2025**, *64* (2), e202414569.

(20) Wang, L.; Fu, S.; Shi, R.; Zhao, Y.; Zhou, H.; Huang, H.; Yu, Z. Q.; Wu, Y. Beyond Second Coordination Shell: Long-Range pi-electrons delocalization Engineering in Single-Atom Catalysts for CO_2_ Electroreduction. *Angew. Chem. Int. Ed.* **2025**, *64*, e202506663.

(21) Zhang, W.; Mehmood, A.; Ali, G.; Liu, H.; Chai, L.; Wu, J.; Liu, M. Nickel Nanocluster-Stabilized Unsaturated Ni-N_3_ Atomic Sites for Efficient CO_2_-to-CO Electrolysis at Industrial-Level Current. *Angew. Chem. Int. Ed.* **2025**, *64* (13), e202424552.

(22) Jin, Z.; Jiao, D.; Dong, Y.; Liu, L.; Fan, J.; Gong, M.; Ma, X.; Wang, Y.; Zhang, W.; Zhang, L.; et al. Boosting Electrocatalytic Carbon Dioxide Reduction via Self-Relaxation of Asymmetric Coordination in Fe-Based Single Atom Catalyst. *Angew. Chem. Int. Ed.* **2024**, *63* (6), e202318246.

(23) Ma, Z.; Wang, B.; Yang, X.; Ma, C.; Wang, W.; Chen, C.; Liang, F.; Zhang, N.; Zhang, H.; Chu, Y.; et al. P-Block Aluminum Single-Atom Catalyst for Electrocatalytic CO_2_ Reduction with High Intrinsic Activity. *J. Am. Chem. Soc.* **2024**, *146* (42), 29140-29149.
